# Bringing the MMFF force field to the RDKit: implementation and validation

**DOI:** 10.1186/s13321-014-0037-3

**Published:** 2014-07-12

**Authors:** Paolo Tosco, Nikolaus Stiefl, Gregory Landrum

**Affiliations:** 1 Department of Drug Science and Technology, University of Turin, Via Pietro Giuria 9, Torino, 10125, Italy; 2 Novartis Institutes for Biomedical Research, Basel CH-4002, Switzerland

**Keywords:** Molecular mechanics, Force field, MMFF, RDKit

## Abstract

A general purpose force field such as MMFF94/MMFF94s, which can properly deal with a wide range of diverse structures, is very valuable in the context of a cheminformatics toolkit. Herein we present an open-source implementation of this force field within the RDKit. The new MMFF functionality can be accessed through a C++/C#/Python/Java application programming interface (API) developed along the lines of the one already available for UFF in the RDKit. Our implementation was fully validated against the official validation suite provided by the MMFF authors. All energies and gradients were correctly computed; moreover, atom type and force constants were correctly assigned for 3D molecules built from SMILES strings. To provide full flexibility, the available API provides direct access to include/exclude individual terms from the MMFF energy expression and to carry out constrained geometry optimizations. The availability of a MMFF-capable molecular mechanics engine coupled with the rest of the RDKit functionality and covered by the BSD license is appealing to researchers operating in both academia and industry.

## Background

Molecular mechanics force fields are the workhorse of computational chemists for molecular simulations, owing to their low computational demands compared to CPU-intensive quantum mechanical methods. Drug designers and cheminformaticians are typically most interested in general purpose force fields, namely those which deliver good performance over a broad spectrum of different structures, ranging from biological macromolecules to small drug-like molecules. Such force fields are ideal to optimize 3D conformations of ligands or receptor/ligand complexes in batch workflows, because they seldom fail due to missing parameters. Examples of generalized force-fields include UFF [[1]], MMFF94 [[2]-[8]], OPLS [[9],[10]], GAFF [[11]] and CGenFF [[12]].

Among these, one of the most widely used and appreciated is MMFF94, which was developed about twenty years ago by Halgren at Merck Research Laboratories and documented by a series of five papers on J. Comput. Chem. [[2]-[6]]. A couple of years later, MMFF94 was complemented by the “static” MMFF94s variant [[7]], which is characterized by a different parameterization of torsional and out-of-plane interactions geared towards geometry optimization studies (MMFF94 was recommended for molecular dynamics simulations). For the remainder of this paper, “MMFF” will collectively refer to both MMFF94 and MMFF94s variants. MMFF has shown robustness and quality in dealing with small molecules superior to the majority of generalized force fields in use at the time of its first introduction [[8],[13]], and also on par or better than the more recent OPLS2005 and GAFF force fields [[14]]. Its versatility and effectiveness warranted its implementation in all major commercial molecular modelling packages (CCG MOE, Schrödinger Maestro, MolSoft ICM, Certara SYBYL-X) and cheminformatics toolkits (ChemAxon, OpenEye) currently on the market.

Herein we present an implementation of MMFF within the open-source cheminformatics toolkit RDKit [[15]]; MMFF functionality can be accessed through C++, C#, Python and Java application programming interfaces (APIs). While other non-commercial implementations of MMFF do exist [[16]-[19]], ours is a complete implementation according to the definition given by Kearsley [[20],[21]], and, to the best of our knowledge, the only such one available under the permissive BSD 3-clause license.

## Implementation

The MMFF energy expression is constituted by seven terms: bond stretching, angle bending, stretch-bend, out-of-plane bending, torsional, van der Waals and electrostatic (Equation 1); the functional form of individual terms is reported in the original literature [[2]].

(1) $$E_{\mathrm{MMFF}}=\sum {\mathrm{EB}}_{\mathrm{ij}}+\sum {\mathrm{EA}}_{\mathrm{ijk}}+\sum {\mathrm{EBA}}_{\mathrm{ijk}}+\sum {\mathrm{EOOP}}_{\mathrm{ijk};l}+\sum {\mathrm{ET}}_{\mathrm{ijkl}}+\sum {\mathrm{EvdW}}_{\mathrm{ij}}+\sum {\mathrm{EQ}}_{\mathrm{ij}}$$

The first step in building the force field for a given molecular system is assigning correct types to each atom. MMFF identifies 216 symbolic atom types, based on the chemical nature and environment of each atom, which are encoded into 95 numeric atom types. Correct atom typing critically depends on the proper attribution of the aromaticity flags. Since the aromaticity model used by MMFF differs from the one normally used throughout the RDKit, aromaticity has to be re-perceived according to MMFF criteria starting from a kekulized representation of the molecule. Subsequently, atom types are assigned to heavy atoms followed by hydrogens.

As a second step, atom-centered partial charges are computed according to the MMFF charge model [[6]], which requires assignment of formal charges. The MMFF formal charge paradigm is based on resonant charges distributed over heteroatoms of the respective functional groups, which is different to the one implemented in the RDKit. In the API, atom types and charges are assigned upon construction of an instance of the MMFFMolProperties class. This class includes methods to choose between the MMFF94/MMFF94s variants, to access atom types and partial charges, to set the dielectric model (constant or distance-dependent) and the dielectric constant, or to exclude selected terms from the energy expression (Equation 1), respectively.

By calling the constructForceField() function, or its Python counterpart MMFFGetMoleculeForceField(), all bonded and non-bonded interactions in the molecular system under study, depending on its structure and connectivity, are loaded into the energy expression. Force constants and equilibrium values for bonded interactions are retrieved from the tables attached to the MMFF papers [[22]] by means of a binary search algorithm as recommended by Halgren. We also tried the map object implemented in the standard C++ library, which indexes database elements through hash tables, but it proved slightly slower. For interactions lacking a specific parameterization, a staged “step-down” procedure is carried out, in which increasingly generic values are sought [[2]].

Optionally, external restraining terms can be added to the MMFF energy expression, with the purpose of constraining selected internal coordinates during geometry optimizations. This feature may prove valuable in a number of instances; e.g., to relax ligand-receptor complexes without causing major alterations of their original geometry, or to perform a torsional scan on a selected dihedral while relaxing the rest of the molecule. Restraints have been implemented as flat-bottomed potentials of user-defined strength, whose functional forms for distances *r*_*ij*_, angles *ϑ*_*ijk*_, and torsions *ϕ*_*ijkl*_ are reported in Equations 2-4, respectively:

(2) $$E_{\mathrm{distance}}=\{\begin{array}{l} \frac{1}{2}k{\left(r_{\mathrm{ij}}-r_{\text{min}}\right)}^{2}r_{\mathrm{ij}}<r_{\text{min}}\\ 0r_{\text{min}}\leq r_{\mathrm{ij}}\leq r_{\text{max}}\\ \frac{1}{2}k{\left(r_{\mathrm{ij}}-r_{\text{max}}\right)}^{2}r_{\mathrm{ij}}>r_{\text{max}} \end{array}$$

(3) $$E_{\mathrm{angle}}=\{\begin{array}{l} \frac{1}{2}k{\left(\vartheta_{\mathrm{ijk}}-\vartheta_{\text{min}}\right)}^{2}\vartheta_{\mathrm{ijk}}<\vartheta_{\text{min}}\\ 0\vartheta_{\text{min}}\leq \vartheta_{\mathrm{ijk}}\leq \vartheta_{\text{max}}\\ \frac{1}{2}k{\left(\vartheta_{\mathrm{ijk}}-\vartheta_{\text{max}}\right)}^{2}\vartheta_{\mathrm{ijk}}>\vartheta_{\text{max}} \end{array}$$

(4) $$E_{\mathrm{torsion}}=\{\begin{array}{l} \frac{1}{2}k{\left(\varphi_{\mathrm{ijkl}}-\varphi_{\text{min}}\right)}^{2}\varphi_{\mathrm{ijkl}}<\varphi_{\text{min}}\\ 0\varphi_{\text{min}}\leq \varphi_{\mathrm{ijkl}}\leq \varphi_{\text{max}}\\ \frac{1}{2}k{\left(\varphi_{\mathrm{ijkl}}-\varphi_{\text{max}}\right)}^{2}\varphi_{\mathrm{ijkl}}>\varphi_{\text{max}} \end{array}$$

Flat-bottomed potentials are enforced only when the relevant internal coordinate is outside the (min, max) range; the latter can be defined in absolute terms or relative to the current geometry. Moreover, positional Cartesian restraints can be set on individual atoms (including dummy atoms, such as the center of mass of a residue) as per Equation 5:

(5) $$E_{\mathrm{positional}}=\{\begin{array}{l} 0r_{\mathrm{ij}}\leq r_{\text{max}}\\ \frac{1}{2}k{\left(r_{\mathrm{ij}}-r_{\text{max}}\right)}^{2}r_{\mathrm{ij}}>r_{\text{max}} \end{array}$$

In this case, the constraint is applied whenever the atom in question moves farther than *r*_max_ from its original position. The possibility of adding these restraining potentials to the force field expression has also been added to the existing RDKit UFF implementation.

Once all bonded and non-bonded interactions, plus optional restraints, have been loaded into the MMFF energy expression, potential energy and gradients of the system under study can be computed or minimized via RDKit’s ForceFields::ForceField::calcEnergy(), ForceFields:: ForceField::calcGrad() and ForceFields::ForceField::minimize() methods. A few examples of simple and constrained geometry optimizations and potential energy calculations are available as Additional files 1, 2 and 3; more examples can be found in the RDKit test suite (both C++ and Python).

## Results and discussion

Our MMFF implementation was validated against the MMFF94 and MMFF94s official validation suites deposited by Halgren and Kearsley in the CCL data archive [[20],[21]], which contain the kekulized 3D structures of 761 and 235 molecules, respectively. Each coordinate file is provided with two different representations of bonds to sulfur and phosphorous atoms, namely dative (i.e., single bonds with formal charge separation) and hypervalent (i.e., double bonds with no formal charge separation). Since the MOL2 format used for the 3D coordinates does not allow explicit indication of formal charges, the authors included two text files where atoms bearing formal charges on the various molecules are listed for the dative and hypervalent representations, respectively. For validation purposes, we combined this information into SD files which can be found in the Additional files 1, 2 and 3. The validation suites include a detailed log file generated by OPTIMOL, a molecular-mechanics program developed at Merck where the force field was first implemented, which lists for each molecule the MMFF atom types and charges and the overall potential energy, decomposed into the seven energy terms which appear in Equation 1. Additionally, for each energy term the list of individual bonded interactions is reported, along with equilibrium values and force constants. The detailed information reported in this log file enables a thorough, in-depth validation of all steps involved in MMFF energy calculations, in particular:

 correct assignment of atom types and charges;

 enumeration of bonded and non-bonded interactions and correct assignment/calculation of equilibrium values and force constants;

 correct calculation of the energy contribution for each interaction.

Our implementation passes all validation tests for both MMFF94 and MMFF94s suites; hence, it can be labelled as a complete MMFF implementation according to the cited criteria given by Kearsley [[20],[21]]. The validation C++ code is included as part of the standard RDKit test suite, and is also available as Additional files 1, 2 and 3. On top of the official validation, we performed three additional robustness tests. Firstly, since the OPTIMOL log file does not include per-atom energy gradients, we checked them against those computed by the MMFF implementation available in TINKER [[17]], and they proved to be identical. Secondly, we converted the 3D structures provided in the MMFF validation suites into SMILES strings using the MolToSmiles() RDKit function, and then rebuilt 3D coordinates with DGeomHelpers::EmbedMolecule(); the raw 3D geometries were finally optimized with our MMFF implementation (see Additional files 1, 2 and 3). Obviously, since now the rebuilt conformations were different from those in the validation suite, energies could not be compared anymore. Still, all atom types, charges and force constants were correctly assigned. This proves the robustness of our assignment algorithms against variations of bond orders due to degenerate kekulization of condensed aromatic systems. Finally, upon suggestion of a reviewer, we challenged the atom typing code against 100 random shuffles of the atom order in the input coordinate files, obtaining correct results in all cases.

## Conclusions

We presented a complete and validated implementation of the MMFF94/MMFF94s force fields within the open-source cheminformatics toolkit RDKit. The integration with the RDKit, which is licensed under a 3-clause BSD license, makes this implementation appealing to both academic and industrial users. The MMFF-related C++, C#, Python and Java APIs have the same architecture as those previously implemented for UFF; hence, it is straightforward to switch existing programs and scripts which relied upon the RDKit UFF force field implementation to MMFF. A comprehensive application to computer-aided drug design of the MMFF implementation described herein will be the subject of a forthcoming paper.

## Availability and requirements

The MMFF implementation object of this work is included in the BSD-licensed open source toolkit RDKit [[15]] since Release_2013.09.1, and can be accessed from C++, C#, Python and Java applications.

## Competing interests

The authors declare that they have no competing interests.

## Authors’ contributions

PT did the C++/Python implementation of MMFF within the RDKit and drafted the manuscript. NS and GL conceived the original project, supervised and contributed to the implementation and validation of MMFF within the RDKit, and helped draft the manuscript. All authors contributed to the discussion of results, read and approved the final manuscript.

## Additional files

## Supplementary Material

Additional file 1: **Source code.** The file sources.zip contains: ● the C++ sources of the MMFF-related code implemented within the RDKit; ● the Python script mol2ToSdfAndSmi.py, which converts the MOL2 and formal charge files included in the original MMFF validation suite into SDF and SMILES representations; ● the Python script shuffleSdf.py, which shuffles the order of the atoms of molecules in a SDF file; ● the Bash script shuffleValidation.sh, that we used to test the robustness of the atom typing code against 100 random shuffles of the atom order in the input coordinate files; ● the commented Python scripts optFromSmiles.py, optLigandInProtein.py and torsionalScan.py which serve as examples of simple and constrained MMFF minimizations. Click here for file

Additional file 2: **Structures.** The file structures.zip contains: ● the SDF 3D coordinate files MMFF94_dative.sdf, MMFF94_hypervalent.sdf, MMFF94s_dative.sdf, MMFF94s_hypervalent.sdf, and the respective SMILES representations MMFF94_dative.smi, MMFF94_hypervalent.smi, MMFF94s_dative.smi, MMFF94s_hypervalent.smi, as generated by the mol2ToSdfAndSmi.py Python script; ● the SDF 3D coordinate files MMFF94_dative_min_from_SMILES.sdf, MMFF94_hypervalent_min_from_SMILES.sdf, MMFF94s_dative_min_from_SMILES.sdf, MMFF94s_hypervalent_min_from_SMILES.sdf, as rebuilt by the program testMMFFForceField out of the corresponding SMILES representations. Click here for file

Additional file 3: **Documentation.** The file docs.zip expands to an HTML tree which documents the MMFF-related C++ and Python RDKit APIs; the documentation can be browsed opening the docs.html file in any HTML browser. The full RDKit documentation can be found at http://www.rdkit.org. Click here for file
