# Supplementary material for: Bringing the MMFF force field to the RDKit: implementation and validation
Source: J Cheminform. 2014 Jul 12;6:37. doi: 10.1186/s13321-014-0037-3 (PMC4116604; doi:10.1186/s13321-014-0037-3)
Supplement: Additional file 3: — Documentation. The file docs.zip expands to an HTML tree which documents the MMFF-related C++ and Python RDKit APIs; the documentation can be browsed opening the docs.html file in any HTML browser. The full RDKit documentation can be found at http://www.rdkit.org. [file s13321-014-0037-3-S3.zip › docs/cpp/AngleBend_8h.html]

RDKit-MMFF: AngleBend.h File Reference


- Main Page
- Namespaces
- Classes
- Files
- Directories

- File List
- File Members

ForceField » MMFF

# AngleBend.h File Reference

`#include <ForceField/ForceField.h>`  
`#include <ForceField/Contrib.h>`  

Go to the source code of this file.

|  |  |
| --- | --- |
| Classes | |
| class | ForceFields::MMFF::AngleBendContrib |
|  | The angle-bend term for MMFF. More... |
| Namespaces | |
| namespace | ForceFields |
| namespace | ForceFields::MMFF |
| namespace | ForceFields::MMFF::Utils |
| Functions | |
| double | ForceFields::MMFF::Utils::calcAngleRestValue (const MMFFAngle \*mmffAngleParams) |
|  | returns the MMFF rest value for an angle |
| double | ForceFields::MMFF::Utils::calcAngleForceConstant (const MMFFAngle \*mmffAngleParams) |
|  | returns the MMFF force constant for an angle |
| double | ForceFields::MMFF::Utils::calcCosTheta (RDGeom::Point3D p1, RDGeom::Point3D p2, RDGeom::Point3D p3, double dist1, double dist2) |
|  | calculates and returns the cosine of the angle between points p1, p2, p3 |
| double | ForceFields::MMFF::Utils::calcAngleBendEnergy (const double theta0, const double ka, bool isLinear, const double cosTheta) |
|  | calculates and returns the angle bending MMFF energy |
| void | ForceFields::MMFF::Utils::calcAngleBendGrad (RDGeom::Point3D \*r, double \*dist, double \*\*g, double &dE\_dTheta, double &cosTheta, double &sinTheta) |

---

Generated on 16 Feb 2014 for RDKit-MMFF by 
 1.6.1 
