# Supplementary material for: Bringing the MMFF force field to the RDKit: implementation and validation
Source: J Cheminform. 2014 Jul 12;6:37. doi: 10.1186/s13321-014-0037-3 (PMC4116604; doi:10.1186/s13321-014-0037-3)
Supplement: Additional file 3: — Documentation. The file docs.zip expands to an HTML tree which documents the MMFF-related C++ and Python RDKit APIs; the documentation can be browsed opening the docs.html file in any HTML browser. The full RDKit documentation can be found at http://www.rdkit.org. [file s13321-014-0037-3-S3.zip › docs/cpp/AngleBend_8h_source.html]

RDKit-MMFF: AngleBend.h Source File


- Main Page
- Namespaces
- Classes
- Files
- Directories

- File List
- File Members

ForceField » MMFF

# AngleBend.h

Go to the documentation of this file.

```
00001 //
00002 //  Copyright (C) 2013 Paolo Tosco
00003 //
00004 //  Copyright (C) 2004-2006 Rational Discovery LLC
00005 //
00006 //   @@ All Rights Reserved @@
00007 //  This file is part of the RDKit.
00008 //  The contents are covered by the terms of the BSD license
00009 //  which is included in the file license.txt, found at the root
00010 //  of the RDKit source tree.
00011 //
00012 #ifndef __RD_MMFFANGLEBEND_H__
00013 #define __RD_MMFFANGLEBEND_H__
00014 
00015 #include <ForceField/ForceField.h>
00016 #include <ForceField/Contrib.h>
00017 
00018 namespace ForceFields {
00019   namespace MMFF {
00020     class MMFFBond;
00021     class MMFFAngle;
00022     class MMFFProp;
00023 
00024     //! The angle-bend term for MMFF
00025     class AngleBendContrib : public ForceFieldContrib {
00026     public:
00027       AngleBendContrib() : d_at1Idx(-1), d_at2Idx(-1), d_at3Idx(-1) {};
00028       //! Constructor
00029       /*!
00030         The angle is between atom1 - atom2 - atom3
00031         
00032         \param owner       pointer to the owning ForceField
00033         \param idx1        index of atom1 in the ForceField's positions
00034         \param idx2        index of atom2 in the ForceField's positions
00035         \param idx3        index of atom3 in the ForceField's positions
00036         \param angleType   MMFF type of the angle (as an unsigned int)
00037         
00038       */
00039       AngleBendContrib(ForceField *owner,
00040         unsigned int idx1, unsigned int idx2, unsigned int idx3,
00041         const MMFFAngle *mmffAngleParams, const MMFFProp *mmffPropParamsCentralAtom);
00042       double getEnergy(double *pos) const;
00043       void getGrad(double *pos,double *grad) const;
00044     
00045     private:
00046       bool d_isLinear;
00047       int d_at1Idx, d_at2Idx, d_at3Idx;
00048       double d_ka, d_theta0;
00049     };
00050     namespace Utils {
00051       //! returns the MMFF rest value for an angle
00052       double calcAngleRestValue(const MMFFAngle *mmffAngleParams);
00053       //! returns the MMFF force constant for an angle 
00054       double calcAngleForceConstant(const MMFFAngle *mmffAngleParams);
00055       //! calculates and returns the cosine of the angle between points p1, p2, p3
00056       double calcCosTheta(RDGeom::Point3D p1, RDGeom::Point3D p2,
00057         RDGeom::Point3D p3, double dist1, double dist2);
00058       //! calculates and returns the angle bending MMFF energy
00059       double calcAngleBendEnergy(const double theta0,
00060         const double ka, bool isLinear, const double cosTheta);
00061       void calcAngleBendGrad(RDGeom::Point3D *r, double *dist,
00062         double **g, double &dE_dTheta, double &cosTheta, double &sinTheta);
00063     }
00064   }
00065 }
00066 #endif
```

---

Generated on 16 Feb 2014 for RDKit-MMFF by 
 1.6.1 
