# Supplementary material for: Bringing the MMFF force field to the RDKit: implementation and validation
Source: J Cheminform. 2014 Jul 12;6:37. doi: 10.1186/s13321-014-0037-3 (PMC4116604; doi:10.1186/s13321-014-0037-3)
Supplement: Additional file 3: — Documentation. The file docs.zip expands to an HTML tree which documents the MMFF-related C++ and Python RDKit APIs; the documentation can be browsed opening the docs.html file in any HTML browser. The full RDKit documentation can be found at http://www.rdkit.org. [file s13321-014-0037-3-S3.zip › docs/cpp/AngleConstraint_8h_source.html]

RDKit-MMFF: AngleConstraint.h Source File


- Main Page
- Namespaces
- Classes
- Files
- Directories

- File List
- File Members

ForceField » MMFF

# AngleConstraint.h

Go to the documentation of this file.

```
00001 //
00002 //  Copyright (C) 2013 Paolo Tosco
00003 //
00004 //  Copyright (C) 2004-2006 Rational Discovery LLC
00005 //
00006 //   @@ All Rights Reserved @@
00007 //  This file is part of the RDKit.
00008 //  The contents are covered by the terms of the BSD license
00009 //  which is included in the file license.txt, found at the root
00010 //  of the RDKit source tree.
00011 //
00012 #ifndef __RD_MMFFANGLECONSTRAINT_H__
00013 #define __RD_MMFFANGLECONSTRAINT_H__
00014 #include <iostream>
00015 #include <ForceField/Contrib.h>
00016 
00017 namespace ForceFields {
00018   namespace MMFF {
00019 
00020     //! An angle range constraint modelled after a AngleBendContrib
00021     class AngleConstraintContrib : public ForceFieldContrib {
00022     public:
00023       AngleConstraintContrib() : d_at1Idx(-1), d_at2Idx(-1), d_at3Idx(-1) {};
00024       //! Constructor
00025       /*!
00026       \param owner       pointer to the owning ForceField
00027       \param idx1        index of atom1 in the ForceField's positions
00028       \param idx2        index of atom2 in the ForceField's positions
00029       \param idx3        index of atom3 in the ForceField's positions
00030       \param minAngle    minimum angle
00031       \param maxAngle    maximum angle
00032       \param forceConst  force Constant
00033         
00034       */
00035       AngleConstraintContrib(ForceField *owner, unsigned int idx1, unsigned int idx2,
00036                                 unsigned int idx3, double minAngleDeg, double maxAngleDeg, double forceConst);
00037       AngleConstraintContrib(ForceField *owner, unsigned int idx1, unsigned int idx2,
00038                                 unsigned int idx3, bool relative, double minAngleDeg, double maxAngleDeg,
00039         double forceConst);
00040 
00041       ~AngleConstraintContrib() {
00042       }
00043       double getEnergy(double *pos) const;
00044 
00045       void getGrad(double *pos, double *grad) const;
00046     private:
00047       int d_at1Idx, d_at2Idx, d_at3Idx; //!< indices of atoms forming the angle
00048       double d_minAngleDeg, d_maxAngleDeg;        //!< rest amplitudes of the angle
00049       double d_forceConstant;  //!< force constant of the angle constraint
00050 
00051     };
00052   }
00053 }
00054 #endif
```

---

Generated on 16 Feb 2014 for RDKit-MMFF by 
 1.6.1 
