# Supplementary material for: Bringing the MMFF force field to the RDKit: implementation and validation
Source: J Cheminform. 2014 Jul 12;6:37. doi: 10.1186/s13321-014-0037-3 (PMC4116604; doi:10.1186/s13321-014-0037-3)
Supplement: Additional file 3: — Documentation. The file docs.zip expands to an HTML tree which documents the MMFF-related C++ and Python RDKit APIs; the documentation can be browsed opening the docs.html file in any HTML browser. The full RDKit documentation can be found at http://www.rdkit.org. [file s13321-014-0037-3-S3.zip › docs/cpp/annotated.html]

RDKit-MMFF: Class List


- Main Page
- Namespaces
- Classes
- Files
- Directories

- Class List
- Class Members

# Class List

Here are the classes, structs, unions and interfaces with brief descriptions:

|  |  |
| --- | --- |
| ForceFields::MMFF::AngleBendContrib | The angle-bend term for MMFF |
| ForceFields::MMFF::AngleConstraintContrib | An angle range constraint modelled after a AngleBendContrib |
| ForceFields::MMFF::BondStretchContrib | The bond-stretch term for MMFF |
| ForceFields::MMFF::DistanceConstraintContrib | A distance range constraint modelled after a BondStretchContrib |
| ForceFields::MMFF::EleContrib | Electrostatic term for MMFF |
| ForceFields::MMFF::MMFFAngle | Class to store MMFF parameters for angle bending |
| ForceFields::MMFF::MMFFAngleCollection |  |
| ForceFields::MMFF::MMFFAromCollection |  |
| RDKit::MMFF::MMFFAtomProperties |  |
| ForceFields::MMFF::MMFFBndkCollection |  |
| ForceFields::MMFF::MMFFBond | Class to store MMFF parameters for bond stretching |
| ForceFields::MMFF::MMFFBondCollection |  |
| ForceFields::MMFF::MMFFChg |  |
| ForceFields::MMFF::MMFFChgCollection |  |
| ForceFields::MMFF::MMFFCovRadPauEle |  |
| ForceFields::MMFF::MMFFCovRadPauEleCollection |  |
| ForceFields::MMFF::MMFFDef | Class to store MMFF atom type equivalence levels |
| ForceFields::MMFF::MMFFDefCollection |  |
| ForceFields::MMFF::MMFFDfsbCollection |  |
| RDKit::MMFF::MMFFMolProperties |  |
| ForceFields::MMFF::MMFFOop | Class to store MMFF parameters for out-of-plane bending |
| ForceFields::MMFF::MMFFOopCollection |  |
| ForceFields::MMFF::MMFFPBCI | Class to store MMFF Partial Bond Charge Increments |
| ForceFields::MMFF::MMFFPBCICollection |  |
| ForceFields::MMFF::MMFFProp | Class to store MMFF Properties |
| ForceFields::MMFF::MMFFPropCollection |  |
| ForceFields::MMFF::MMFFStbn | Class to store MMFF parameters for stretch-bending |
| ForceFields::MMFF::MMFFStbnCollection |  |
| ForceFields::MMFF::MMFFTor | Class to store MMFF parameters for torsions |
| ForceFields::MMFF::MMFFTorCollection |  |
| ForceFields::MMFF::MMFFVdW | Class to store MMFF parameters for non-bonded Van der Waals |
| ForceFields::MMFF::MMFFVdWCollection |  |
| ForceFields::MMFF::OopBendContrib | Out-of-plane term for MMFF |
| ForceFields::MMFF::PositionConstraintContrib | A position constraint of the type 0.5k \* deltaX^2 |
| ForceFields::MMFF::StretchBendContrib | The angle-bend term for MMFF |
| ForceFields::MMFF::TorsionAngleContrib | Torsion term for MMFF |
| ForceFields::MMFF::TorsionConstraintContrib | A dihedral angle range constraint modelled after a TorsionContrib |
| ForceFields::MMFF::VdWContrib | Van der Waals term for MMFF |

---

Generated on 16 Feb 2014 for RDKit-MMFF by 
 1.6.1 
