# Supplementary material for: Bringing the MMFF force field to the RDKit: implementation and validation
Source: J Cheminform. 2014 Jul 12;6:37. doi: 10.1186/s13321-014-0037-3 (PMC4116604; doi:10.1186/s13321-014-0037-3)
Supplement: Additional file 3: — Documentation. The file docs.zip expands to an HTML tree which documents the MMFF-related C++ and Python RDKit APIs; the documentation can be browsed opening the docs.html file in any HTML browser. The full RDKit documentation can be found at http://www.rdkit.org. [file s13321-014-0037-3-S3.zip › docs/cpp/AtomTyper_8h.html]

RDKit-MMFF: AtomTyper.h File Reference


- Main Page
- Namespaces
- Classes
- Files
- Directories

- File List
- File Members

GraphMol » ForceFieldHelpers » MMFF

# AtomTyper.h File Reference

`#include <vector>`  
`#include <string>`  
`#include <iostream>`  
`#include <ForceField/MMFF/Params.h>`  
`#include <boost/cstdint.hpp>`  

Go to the source code of this file.

|  |  |
| --- | --- |
| Classes | |
| class | RDKit::MMFF::MMFFAtomProperties |
| class | RDKit::MMFF::MMFFMolProperties |
| Namespaces | |
| namespace | RDKit |
| namespace | RDKit::MMFF |
| Typedefs | |
| typedef boost::shared\_ptr  < MMFFAtomProperties > | RDKit::MMFF::MMFFAtomPropertiesPtr |
| Enumerations | |
| enum | { RDKit::MMFF::CONSTANT = 1, RDKit::MMFF::DISTANCE = 2 } |
| enum | { RDKit::MMFF::MMFF\_VERBOSITY\_NONE = 0, RDKit::MMFF::MMFF\_VERBOSITY\_LOW = 1, RDKit::MMFF::MMFF\_VERBOSITY\_HIGH = 2 } |
| Functions | |
| unsigned int | RDKit::MMFF::isAngleInRingOfSize3or4 (const ROMol &mol, const unsigned int idx1, const unsigned int idx2, const unsigned int idx3) |
| unsigned int | RDKit::MMFF::isTorsionInRingOfSize4or5 (const ROMol &mol, const unsigned int idx1, const unsigned int idx2, const unsigned int idx3, const unsigned int idx4) |
| bool | RDKit::MMFF::isAtomInAromaticRingOfSize (const Atom \*atom, const unsigned int ringSize) |
| bool | RDKit::MMFF::isAtomNOxide (const Atom \*atom) |
| bool | RDKit::MMFF::areAtomsInSameAromaticRing (const ROMol &mol, const unsigned int idx1, const unsigned int idx2) |
| bool | RDKit::MMFF::areAtomsInSameRingOfSize (const ROMol &mol, const unsigned int ringSize, const unsigned int numAtoms,...) |
| unsigned int | RDKit::MMFF::sanitizeMMFFMol (RWMol &mol) |
| void | RDKit::MMFF::setMMFFAromaticity (RWMol &mol) |
| const unsigned int | RDKit::MMFF::getMMFFStretchBendType (const unsigned int angleType, const unsigned int bondType1, const unsigned int bondType2) |
| const unsigned int | RDKit::MMFF::getPeriodicTableRow (const int atomicNum) |
| const   ForceFields::MMFF::MMFFAngle \* | RDKit::MMFF::getMMFFAngleBendEmpiricalRuleParams (const ROMol &mol, const ForceFields::MMFF::MMFFAngle \*oldMMFFAngleParams, const ForceFields::MMFF::MMFFProp \*mmffPropParamsCentralAtom, const ForceFields::MMFF::MMFFBond \*mmffBondParams1, const ForceFields::MMFF::MMFFBond \*mmffBondParams2, unsigned int idx1, unsigned int idx2, unsigned int idx3) |

---

Generated on 16 Feb 2014 for RDKit-MMFF by 
 1.6.1 
