# Supplementary material for: Bringing the MMFF force field to the RDKit: implementation and validation
Source: J Cheminform. 2014 Jul 12;6:37. doi: 10.1186/s13321-014-0037-3 (PMC4116604; doi:10.1186/s13321-014-0037-3)
Supplement: Additional file 3: — Documentation. The file docs.zip expands to an HTML tree which documents the MMFF-related C++ and Python RDKit APIs; the documentation can be browsed opening the docs.html file in any HTML browser. The full RDKit documentation can be found at http://www.rdkit.org. [file s13321-014-0037-3-S3.zip › docs/cpp/AtomTyper_8h_source.html]

RDKit-MMFF: AtomTyper.h Source File


- Main Page
- Namespaces
- Classes
- Files
- Directories

- File List
- File Members

GraphMol » ForceFieldHelpers » MMFF

# AtomTyper.h

Go to the documentation of this file.

```
00001 //
00002 //  Copyright (C) 2013 Paolo Tosco
00003 //
00004 //  Copyright (C) 2004-2006 Rational Discovery LLC
00005 //
00006 //   @@ All Rights Reserved @@
00007 //  This file is part of the RDKit.
00008 //  The contents are covered by the terms of the BSD license
00009 //  which is included in the file license.txt, found at the root
00010 //  of the RDKit source tree.
00011 //
00012 #ifndef _RD_MMFFATOMTYPER_H__
00013 #define _RD_MMFFATOMTYPER_H__
00014 
00015 #include <vector>
00016 #include <string>
00017 #include <iostream>
00018 #include <ForceField/MMFF/Params.h>
00019 #include <boost/cstdint.hpp>
00020 
00021 
00022 namespace RDKit {
00023   class ROMol;
00024   class RWMol;
00025   class Atom;
00026   class Bond;
00027 
00028   namespace MMFF {
00029     class MMFFAtomProperties {
00030     public:
00031       MMFFAtomProperties() :
00032         mmffAtomType(0),
00033         mmffFormalCharge(0.0),
00034         mmffPartialCharge(0.0) {};
00035       ~MMFFAtomProperties() {};
00036       boost::uint8_t mmffAtomType;
00037       double mmffFormalCharge;
00038       double mmffPartialCharge;
00039     };
00040     
00041     typedef boost::shared_ptr<MMFFAtomProperties> MMFFAtomPropertiesPtr;
00042     enum {
00043       CONSTANT = 1,
00044       DISTANCE = 2
00045     };
00046     enum {
00047       MMFF_VERBOSITY_NONE = 0,
00048       MMFF_VERBOSITY_LOW = 1,
00049       MMFF_VERBOSITY_HIGH = 2
00050     };
00051     class MMFFMolProperties {
00052     public:
00053       MMFFMolProperties(ROMol &mol, std::string mmffVariant = "MMFF94", 
00054         boost::uint8_t verbosity = MMFF_VERBOSITY_NONE,
00055         std::ostream &oStream = std::cout);
00056       ~MMFFMolProperties() {};
00057       const unsigned int getMMFFBondType(const Bond *bond);
00058       const unsigned int getMMFFAngleType(const ROMol &mol,
00059         const unsigned int idx1, const unsigned int idx2,
00060         const unsigned int idx3);
00061       const std::pair<unsigned int, unsigned int> getMMFFTorsionType
00062         (const ROMol &mol, const unsigned int idx1, const unsigned int idx2,
00063         const unsigned int idx3, const unsigned int idx4);
00064       void computeMMFFCharges(const ROMol &mol);
00065       const ForceFields::MMFF::MMFFTor *getMMFFTorsionEmpiricalRuleParams
00066         (const ROMol &mol, unsigned int idx2, unsigned int idx3);
00067       const ForceFields::MMFF::MMFFBond *getMMFFBondStretchEmpiricalRuleParams
00068         (const ROMol &mol, const Bond *bond);
00069       const boost::uint8_t getMMFFAtomType(const unsigned int idx)
00070       {
00071         RANGE_CHECK(0, idx, this->d_MMFFAtomPropertiesPtrVect.size() - 1);
00072         
00073         return this->d_MMFFAtomPropertiesPtrVect[idx]->mmffAtomType;
00074       };
00075       const double getMMFFFormalCharge(const unsigned int idx)
00076       {
00077         RANGE_CHECK(0, idx, this->d_MMFFAtomPropertiesPtrVect.size() - 1);
00078         
00079         return this->d_MMFFAtomPropertiesPtrVect[idx]->mmffFormalCharge;
00080       };
00081       const double getMMFFPartialCharge(const unsigned int idx)
00082       {
00083         RANGE_CHECK(0, idx, this->d_MMFFAtomPropertiesPtrVect.size() - 1);
00084         
00085         return this->d_MMFFAtomPropertiesPtrVect[idx]->mmffPartialCharge;
00086       };
00087       void setMMFFBondTerm(const bool state)
00088       {
00089         this->d_bondTerm = state;
00090       };
00091       const bool getMMFFBondTerm()
00092       {
00093         return this->d_bondTerm;
00094       };
00095       void setMMFFAngleTerm(const bool state)
00096       {
00097         this->d_angleTerm = state;
00098       };
00099       const bool getMMFFAngleTerm()
00100       {
00101         return this->d_angleTerm;
00102       };
00103       void setMMFFStretchBendTerm(const bool state)
00104       {
00105         this->d_stretchBendTerm = state;
00106       };
00107       const bool getMMFFStretchBendTerm()
00108       {
00109         return this->d_stretchBendTerm;
00110       };
00111       void setMMFFOopTerm(const bool state)
00112       {
00113         this->d_oopTerm = state;
00114       };
00115       const bool getMMFFOopTerm()
00116       {
00117         return this->d_oopTerm;
00118       };
00119       void setMMFFTorsionTerm(const bool state)
00120       {
00121         this->d_torsionTerm = state;
00122       };
00123       const bool getMMFFTorsionTerm()
00124       {
00125         return this->d_torsionTerm;
00126       };
00127       void setMMFFVdWTerm(const bool state)
00128       {
00129         this->d_vdWTerm = state;
00130       };
00131       const bool getMMFFVdWTerm()
00132       {
00133         return this->d_vdWTerm;
00134       };
00135       void setMMFFEleTerm(const bool state)
00136       {
00137         this->d_eleTerm = state;
00138       };
00139       const bool getMMFFEleTerm()
00140       {
00141         return this->d_eleTerm;
00142       };
00143       void setMMFFVariant(const std::string mmffVariant)
00144       {
00145         PRECONDITION((mmffVariant == "MMFF94")
00146           || (mmffVariant == "MMFF94s"), "bad MMFF variant");
00147         
00148         this->d_mmffs = ((mmffVariant == "MMFF94s") ? true : false);
00149       };
00150       const std::string getMMFFVariant()
00151       {
00152         return (this->d_mmffs ? "MMFF94s" : "MMFF94");
00153       };
00154       void setMMFFDielectricConstant(const double dielConst)
00155       {
00156         PRECONDITION(dielConst > 0.0, "bad dielectric constant");
00157         
00158         this->d_dielConst = dielConst;
00159       };
00160       double getMMFFDielectricConstant()
00161       {
00162         return this->d_dielConst;
00163       };
00164       void setMMFFDielectricModel(boost::uint8_t dielModel)
00165       {
00166         this->d_dielModel = dielModel;
00167       };
00168       boost::uint8_t getMMFFDielectricModel()
00169       {
00170         return this->d_dielModel;
00171       };
00172       void setMMFFVerbosity(boost::uint8_t verbosity)
00173       {
00174         this->d_verbosity = verbosity;
00175       };
00176       boost::uint8_t getMMFFVerbosity()
00177       {
00178         return this->d_verbosity;
00179       };
00180       void setMMFFOStream(std::ostream *oStream)
00181       {
00182         this->d_oStream = oStream;
00183       };
00184       std::ostream& getMMFFOStream()
00185       {
00186         return *(this->d_oStream);
00187       };
00188       bool isValid()
00189       {
00190         return d_valid;
00191       };
00192     private:
00193       void setMMFFHeavyAtomType(const Atom *atom);
00194       void setMMFFHydrogenType(const Atom *atom);
00195       void setMMFFFormalCharge(const unsigned int idx, const double fChg)
00196       {
00197         RANGE_CHECK(0, idx, this->d_MMFFAtomPropertiesPtrVect.size() - 1);
00198         
00199         this->d_MMFFAtomPropertiesPtrVect[idx]->mmffFormalCharge = fChg;
00200       };
00201       void setMMFFPartialCharge(const unsigned int idx, const double pChg)
00202       {
00203         RANGE_CHECK(0, idx, this->d_MMFFAtomPropertiesPtrVect.size() - 1);
00204         
00205         this->d_MMFFAtomPropertiesPtrVect[idx]->mmffPartialCharge = pChg;
00206       };
00207       bool d_valid;
00208       bool d_mmffs;
00209       bool d_bondTerm;
00210       bool d_angleTerm;
00211       bool d_stretchBendTerm;
00212       bool d_oopTerm;
00213       bool d_torsionTerm;
00214       bool d_vdWTerm;
00215       bool d_eleTerm;
00216       double d_dielConst;       //!< the dielectric constant
00217       boost::uint8_t d_dielModel; //!< the dielectric model (1 = constant, 2 = distance-dependent)
00218       boost::uint8_t d_verbosity;
00219       std::ostream *d_oStream;
00220       std::vector<MMFFAtomPropertiesPtr> d_MMFFAtomPropertiesPtrVect;
00221     };
00222     unsigned int isAngleInRingOfSize3or4(const ROMol &mol, const unsigned int idx1,
00223       const unsigned int idx2, const unsigned int idx3);
00224     unsigned int isTorsionInRingOfSize4or5(const ROMol &mol, const unsigned int idx1,
00225       const unsigned int idx2, const unsigned int idx3, const unsigned int idx4);
00226     bool isAtomInAromaticRingOfSize(const Atom *atom, const unsigned int ringSize);
00227     bool isAtomNOxide(const Atom *atom);
00228     bool areAtomsInSameAromaticRing(const ROMol &mol,
00229       const unsigned int idx1, const unsigned int idx2);
00230     bool areAtomsInSameRingOfSize(const ROMol &mol,
00231       const unsigned int ringSize, const unsigned int numAtoms, ...);
00232     unsigned int sanitizeMMFFMol(RWMol &mol);
00233     void setMMFFAromaticity(RWMol &mol);
00234     const unsigned int getMMFFStretchBendType(const unsigned int angleType,
00235       const unsigned int bondType1, const unsigned int bondType2);
00236     const unsigned int getPeriodicTableRow(const int atomicNum);
00237     const ForceFields::MMFF::MMFFAngle *getMMFFAngleBendEmpiricalRuleParams
00238       (const ROMol &mol, const ForceFields::MMFF::MMFFAngle *oldMMFFAngleParams,
00239       const ForceFields::MMFF::MMFFProp *mmffPropParamsCentralAtom,
00240       const ForceFields::MMFF::MMFFBond *mmffBondParams1,
00241       const ForceFields::MMFF::MMFFBond *mmffBondParams2,
00242       unsigned int idx1, unsigned int idx2, unsigned int idx3);
00243   }
00244 }
00245 
00246 
00247 #endif
```

---

Generated on 16 Feb 2014 for RDKit-MMFF by 
 1.6.1 
