# Supplementary material for: Bringing the MMFF force field to the RDKit: implementation and validation
Source: J Cheminform. 2014 Jul 12;6:37. doi: 10.1186/s13321-014-0037-3 (PMC4116604; doi:10.1186/s13321-014-0037-3)
Supplement: Additional file 3: — Documentation. The file docs.zip expands to an HTML tree which documents the MMFF-related C++ and Python RDKit APIs; the documentation can be browsed opening the docs.html file in any HTML browser. The full RDKit documentation can be found at http://www.rdkit.org. [file s13321-014-0037-3-S3.zip › docs/cpp/BondStretch_8h_source.html]

RDKit-MMFF: BondStretch.h Source File


- Main Page
- Namespaces
- Classes
- Files
- Directories

- File List
- File Members

ForceField » MMFF

# BondStretch.h

Go to the documentation of this file.

```
00001 //
00002 //  Copyright (C) 2013 Paolo Tosco
00003 //
00004 //  Copyright (C) 2004-2006 Rational Discovery LLC
00005 //
00006 //   @@ All Rights Reserved @@
00007 //  This file is part of the RDKit.
00008 //  The contents are covered by the terms of the BSD license
00009 //  which is included in the file license.txt, found at the root
00010 //  of the RDKit source tree.
00011 //
00012 #ifndef __RD_MMFFBONDSTRETCH_H__
00013 #define __RD_MMFFBONDSTRETCH_H__
00014 #include <ForceField/Contrib.h>
00015 
00016 namespace ForceFields {
00017   namespace MMFF {
00018     class MMFFBond;
00019     class MMFFBondStretchEmpiricalRule;
00020 
00021     //! The bond-stretch term for MMFF
00022     class BondStretchContrib : public ForceFieldContrib {
00023     public:
00024       BondStretchContrib() : d_at1Idx(-1), d_at2Idx(-1) {};
00025       //! Constructor
00026       /*!
00027         \param owner       pointer to the owning ForceField
00028         \param idx1        index of end1 in the ForceField's positions
00029         \param idx2        index of end2 in the ForceField's positions
00030         \param bondType    MMFF94 type of the bond (as an unsigned int)
00031         \param end1Params  pointer to the parameters for end1
00032         \param end2Params  pointer to the parameters for end2
00033         
00034       */
00035       BondStretchContrib(ForceField *owner,
00036         const unsigned int idx1, const unsigned int idx2,
00037         const MMFFBond *mmffBondParams);
00038 
00039       double getEnergy(double *pos) const;
00040 
00041       void getGrad(double *pos,double *grad) const;
00042     
00043     private:
00044       int d_at1Idx, d_at2Idx; //!< indices of end points
00045       double d_r0;        //!< rest length of the bond
00046       double d_kb;  //!< force constant of the bond
00047 
00048     };
00049   
00050     namespace Utils {
00051       //! returns the MMFF rest length for a bond 
00052       double calcBondRestLength(const MMFFBond *mmffBondParams);
00053       //! returns the MMFF force constant for a bond 
00054       double calcBondForceConstant(const MMFFBond *mmffBondParams);
00055       //! calculates and returns the bond stretching MMFF energy
00056       double calcBondStretchEnergy(const double r0, const double kb, const double distance);
00057     }  
00058   }
00059 }
00060 #endif
```

---

Generated on 16 Feb 2014 for RDKit-MMFF by 
 1.6.1 
