# Supplementary material for: Bringing the MMFF force field to the RDKit: implementation and validation
Source: J Cheminform. 2014 Jul 12;6:37. doi: 10.1186/s13321-014-0037-3 (PMC4116604; doi:10.1186/s13321-014-0037-3)
Supplement: Additional file 3: — Documentation. The file docs.zip expands to an HTML tree which documents the MMFF-related C++ and Python RDKit APIs; the documentation can be browsed opening the docs.html file in any HTML browser. The full RDKit documentation can be found at http://www.rdkit.org. [file s13321-014-0037-3-S3.zip › docs/cpp/Builder_8h.html]

RDKit-MMFF: Builder.h File Reference


- Main Page
- Namespaces
- Classes
- Files
- Directories

- File List
- File Members

GraphMol » ForceFieldHelpers » MMFF

# Builder.h File Reference

`#include <vector>`  
`#include <string>`  
`#include <boost/shared_array.hpp>`  
`#include <boost/tuple/tuple.hpp>`  
`#include <boost/cstdint.hpp>`  

Go to the source code of this file.

|  |  |
| --- | --- |
| Namespaces | |
| namespace | ForceFields |
| namespace | RDKit |
| namespace | RDKit::MMFF |
| namespace | RDKit::MMFF::Tools |
| Enumerations | |
| enum | { RDKit::MMFF::Tools::RELATION\_1\_2 = 0, RDKit::MMFF::Tools::RELATION\_1\_3 = 1, RDKit::MMFF::Tools::RELATION\_1\_4 = 2, RDKit::MMFF::Tools::RELATION\_1\_X = 3 } |
| Functions | |
| ForceFields::ForceField \* | RDKit::MMFF::constructForceField (ROMol &mol, double nonBondedThresh=100.0, int confId=-1, bool ignoreInterfragInteractions=true) |
|  | Builds and returns a MMFF force field for a molecule. |
| ForceFields::ForceField \* | RDKit::MMFF::constructForceField (ROMol &mol, MMFFMolProperties \*mmffMolProperties, double nonBondedThresh=100.0, int confId=-1, bool ignoreInterfragInteractions=true) |
|  | Builds and returns a MMFF force field for a molecule. |
| void | RDKit::MMFF::Tools::setTwoBitCell (boost::shared\_array< boost::uint8\_t > &res, unsigned int pos, boost::uint8\_t value) |
| boost::uint8\_t | RDKit::MMFF::Tools::getTwoBitCell (boost::shared\_array< boost::uint8\_t > &res, unsigned int pos) |
| boost::shared\_array  < boost::uint8\_t > | RDKit::MMFF::Tools::buildNeighborMatrix (const ROMol &mol) |
| void | RDKit::MMFF::Tools::addBonds (const ROMol &mol, MMFFMolProperties \*mmffMolProperties, ForceFields::ForceField \*field) |
| void | RDKit::MMFF::Tools::addAngles (const ROMol &mol, MMFFMolProperties \*mmffMolProperties, ForceFields::ForceField \*field) |
| void | RDKit::MMFF::Tools::addStretchBend (const ROMol &mol, MMFFMolProperties \*mmffMolProperties, ForceFields::ForceField \*field) |
| void | RDKit::MMFF::Tools::addOop (const ROMol &mol, MMFFMolProperties \*mmffMolProperties, ForceFields::ForceField \*field) |
| void | RDKit::MMFF::Tools::addTorsions (const ROMol &mol, MMFFMolProperties \*mmffMolProperties, ForceFields::ForceField \*field, std::string torsionBondSmarts="[!$(\*#\*)&!D1]~[!$(\*#\*)&!D1]") |
| void | RDKit::MMFF::Tools::addVdW (const ROMol &mol, int confId, MMFFMolProperties \*mmffMolProperties, ForceFields::ForceField \*field, boost::shared\_array< boost::uint8\_t > neighborMatrix, double nonBondedThresh=100.0, bool ignoreInterfragInteractions=true) |
| void | RDKit::MMFF::Tools::addEle (const ROMol &mol, int confId, MMFFMolProperties \*mmffMolProperties, ForceFields::ForceField \*field, boost::shared\_array< boost::uint8\_t > neighborMatrix, double nonBondedThresh=100.0, bool ignoreInterfragInteractions=true) |

---

Generated on 16 Feb 2014 for RDKit-MMFF by 
 1.6.1 
