# Supplementary material for: Bringing the MMFF force field to the RDKit: implementation and validation
Source: J Cheminform. 2014 Jul 12;6:37. doi: 10.1186/s13321-014-0037-3 (PMC4116604; doi:10.1186/s13321-014-0037-3)
Supplement: Additional file 3: — Documentation. The file docs.zip expands to an HTML tree which documents the MMFF-related C++ and Python RDKit APIs; the documentation can be browsed opening the docs.html file in any HTML browser. The full RDKit documentation can be found at http://www.rdkit.org. [file s13321-014-0037-3-S3.zip › docs/cpp/Builder_8h_source.html]

RDKit-MMFF: Builder.h Source File


- Main Page
- Namespaces
- Classes
- Files
- Directories

- File List
- File Members

GraphMol » ForceFieldHelpers » MMFF

# Builder.h

Go to the documentation of this file.

```
00001 //
00002 //  Copyright (C) 2013 Paolo Tosco
00003 //
00004 //  Copyright (C) 2004-2006 Rational Discovery LLC
00005 //
00006 //   @@ All Rights Reserved @@
00007 //  This file is part of the RDKit.
00008 //  The contents are covered by the terms of the BSD license
00009 //  which is included in the file license.txt, found at the root
00010 //  of the RDKit source tree.
00011 //
00012 #ifndef _RD_MMFFBUILDER_H_
00013 #define _RD_MMFFBUILDER_H_
00014 
00015 #include <vector>
00016 #include <string>
00017 #include <boost/shared_array.hpp>
00018 #include <boost/tuple/tuple.hpp>
00019 #include <boost/cstdint.hpp>
00020 
00021 
00022 namespace ForceFields {
00023   class ForceField;
00024 }
00025 
00026 namespace RDKit {
00027   class ROMol;
00028   namespace MMFF {
00029 
00030     //! Builds and returns a MMFF force field for a molecule
00031     /*!
00032       
00033       \param mol              the molecule to use
00034       \param nonBondedThresh  the threshold to be used in adding non-bonded terms
00035                               to the force field. Any non-bonded contact whose current
00036                         distance is greater than \c nonBondedThresh * the minimum value
00037                         for that contact will not be included.
00038       \param confId     the optional conformer id, if this isn't provided, the molecule's
00039                         default confId will be used.
00040       \param ignoreInterfragInteractions if true, nonbonded terms will not be added between
00041                                          fragments
00042 
00043       \return the new force field. The client is responsible for free'ing this.
00044     */
00045     ForceFields::ForceField *constructForceField(ROMol &mol,
00046       double nonBondedThresh = 100.0, int confId = -1, bool ignoreInterfragInteractions = true);
00047 
00048 
00049     //! Builds and returns a MMFF force field for a molecule
00050     /*!
00051       
00052       \param mol        the molecule to use
00053       \param mmffMolProperties        pointer to a MMFFMolProperties (as obtained by a
00054                         call to setupMMFFForceField())
00055       \param nonBondedThresh  the threshold to be used in adding non-bonded terms
00056                         to the force field. Any non-bonded contact whose current
00057                         distance is greater than \c nonBondedThresh * the minimum value
00058                         for that contact will not be included.
00059       \param confId     the optional conformer id, if this isn't provided, the molecule's
00060                         default confId will be used.
00061       \param ignoreInterfragInteractions if true, nonbonded terms will not be added between
00062                                          fragments
00063     
00064       \return the new force field. The client is responsible for free'ing this.
00065     */
00066     ForceFields::ForceField *constructForceField(ROMol &mol, MMFFMolProperties *mmffMolProperties,
00067       double nonBondedThresh = 100.0, int confId = -1, bool ignoreInterfragInteractions = true);
00068 
00069     namespace Tools {
00070       enum {
00071         RELATION_1_2 = 0,
00072         RELATION_1_3 = 1,
00073         RELATION_1_4 = 2,
00074         RELATION_1_X = 3
00075       };
00076       // these functions are primarily exposed so they can be tested.
00077       void setTwoBitCell(boost::shared_array<boost::uint8_t> &res,
00078         unsigned int pos, boost::uint8_t value);
00079       boost::uint8_t getTwoBitCell
00080         (boost::shared_array<boost::uint8_t> &res, unsigned int pos);
00081       boost::shared_array<boost::uint8_t> buildNeighborMatrix(const ROMol &mol);
00082       void addBonds(const ROMol &mol,
00083         MMFFMolProperties *mmffMolProperties, ForceFields::ForceField *field);
00084       void addAngles(const ROMol &mol,
00085         MMFFMolProperties *mmffMolProperties, ForceFields::ForceField *field);
00086       void addStretchBend(const ROMol &mol,
00087         MMFFMolProperties *mmffMolProperties, ForceFields::ForceField *field);
00088       void addOop(const ROMol &mol,
00089         MMFFMolProperties *mmffMolProperties, ForceFields::ForceField *field);
00090       void addTorsions(const ROMol &mol,
00091         MMFFMolProperties *mmffMolProperties, ForceFields::ForceField *field,
00092         std::string torsionBondSmarts = "[!$(*#*)&!D1]~[!$(*#*)&!D1]");
00093       void addVdW(const ROMol &mol, int confId, MMFFMolProperties *mmffMolProperties,
00094         ForceFields::ForceField *field, boost::shared_array<boost::uint8_t> neighborMatrix,
00095         double nonBondedThresh = 100.0, bool ignoreInterfragInteractions = true);
00096       void addEle(const ROMol &mol, int confId, MMFFMolProperties *mmffMolProperties,
00097         ForceFields::ForceField *field, boost::shared_array<boost::uint8_t> neighborMatrix,
00098         double nonBondedThresh = 100.0, bool ignoreInterfragInteractions = true);
00099     }
00100   }
00101 }
00102 
00103 
00104 #endif
```

---

Generated on 16 Feb 2014 for RDKit-MMFF by 
 1.6.1 
