# Supplementary material for: Bringing the MMFF force field to the RDKit: implementation and validation
Source: J Cheminform. 2014 Jul 12;6:37. doi: 10.1186/s13321-014-0037-3 (PMC4116604; doi:10.1186/s13321-014-0037-3)
Supplement: Additional file 3: — Documentation. The file docs.zip expands to an HTML tree which documents the MMFF-related C++ and Python RDKit APIs; the documentation can be browsed opening the docs.html file in any HTML browser. The full RDKit documentation can be found at http://www.rdkit.org. [file s13321-014-0037-3-S3.zip › docs/cpp/classes.html]

RDKit-MMFF: Alphabetical List


- Main Page
- Namespaces
- Classes
- Files
- Directories

- Class List
- Class Members

# Class Index

A | B | D | E | M | O | P | S | T | V

|  |  |  |  |  |  |  |
| --- | --- | --- | --- | --- | --- | --- |
| |  | | --- | | A | | MMFFAngle (ForceFields::MMFF) | MMFFCovRadPauEleCollection (ForceFields::MMFF) | MMFFPropCollection (ForceFields::MMFF) | |  | | --- | | P | |
| AngleBendContrib (ForceFields::MMFF) | MMFFAngleCollection (ForceFields::MMFF) | MMFFDef (ForceFields::MMFF) | MMFFStbn (ForceFields::MMFF) | PositionConstraintContrib (ForceFields::MMFF) |
| AngleConstraintContrib (ForceFields::MMFF) | MMFFAromCollection (ForceFields::MMFF) | MMFFDefCollection (ForceFields::MMFF) | MMFFStbnCollection (ForceFields::MMFF) | |  | | --- | | S | |
| |  | | --- | | B | | MMFFAtomProperties (RDKit::MMFF) | MMFFDfsbCollection (ForceFields::MMFF) | MMFFTor (ForceFields::MMFF) | StretchBendContrib (ForceFields::MMFF) |
| BondStretchContrib (ForceFields::MMFF) | MMFFBndkCollection (ForceFields::MMFF) | MMFFMolProperties (RDKit::MMFF) | MMFFTorCollection (ForceFields::MMFF) | |  | | --- | | T | |
| |  | | --- | | D | | MMFFBond (ForceFields::MMFF) | MMFFOop (ForceFields::MMFF) | MMFFVdW (ForceFields::MMFF) | TorsionAngleContrib (ForceFields::MMFF) |
| DistanceConstraintContrib (ForceFields::MMFF) | MMFFBondCollection (ForceFields::MMFF) | MMFFOopCollection (ForceFields::MMFF) | MMFFVdWCollection (ForceFields::MMFF) | TorsionConstraintContrib (ForceFields::MMFF) |
| |  | | --- | | E | | MMFFChg (ForceFields::MMFF) | MMFFPBCI (ForceFields::MMFF) | |  | | --- | | O | | |  | | --- | | V | |
| EleContrib (ForceFields::MMFF) | MMFFChgCollection (ForceFields::MMFF) | MMFFPBCICollection (ForceFields::MMFF) | OopBendContrib (ForceFields::MMFF) | VdWContrib (ForceFields::MMFF) |
| |  | | --- | | M | | MMFFCovRadPauEle (ForceFields::MMFF) | MMFFProp (ForceFields::MMFF) |

A | B | D | E | M | O | P | S | T | V

---

Generated on 16 Feb 2014 for RDKit-MMFF by 
 1.6.1 
