# Supplementary material for: Bringing the MMFF force field to the RDKit: implementation and validation
Source: J Cheminform. 2014 Jul 12;6:37. doi: 10.1186/s13321-014-0037-3 (PMC4116604; doi:10.1186/s13321-014-0037-3)
Supplement: Additional file 3: — Documentation. The file docs.zip expands to an HTML tree which documents the MMFF-related C++ and Python RDKit APIs; the documentation can be browsed opening the docs.html file in any HTML browser. The full RDKit documentation can be found at http://www.rdkit.org. [file s13321-014-0037-3-S3.zip › docs/cpp/classForceFields_1_1MMFF_1_1MMFFDefCollection.html]

RDKit-MMFF: ForceFields::MMFF::MMFFDefCollection Class Reference


- Main Page
- Namespaces
- Classes
- Files
- Directories

- Class List
- Class Members

ForceFields::MMFF::MMFFDefCollection

# ForceFields::MMFF::MMFFDefCollection Class Reference

`#include <Params.h>`

List of all members.

|  |  |
| --- | --- |
| Public Member Functions | |
| const MMFFDef \* | operator() (const unsigned int atomType) const |
|  | Looks up the parameters for a particular key and returns them. |
| Static Public Member Functions | |
| static MMFFDefCollection \* | getMMFFDef (const std::string &mmffDef="") |
|  | gets a pointer to the singleton MMFFDefCollection |

---

## Detailed Description

Definition at line 166 of file Params.h.

---

## Member Function Documentation

|  |  |  |  |  |  |
| --- | --- | --- | --- | --- | --- |
| static MMFFDefCollection\* ForceFields::MMFF::MMFFDefCollection::getMMFFDef | ( | const std::string & | *mmffDef* = `""` | ) | `[static]` |

gets a pointer to the singleton MMFFDefCollection

**Parameters:**
:   |  |  |  |
    | --- | --- | --- |
    |  | *mmffDef* | (optional) a string with parameter data. See below for more information about this argument |

**Returns:**
:   a pointer to the singleton MMFFDefCollection

**Notes:**

- do **not** delete the pointer returned here
- if the singleton MMFFDefCollection has already been instantiated and `mmffDef` is empty, the singleton will be returned.
- if `mmffDef` is empty and the singleton MMFFDefCollection has not yet been instantiated, the default MMFFDef parameters (from Params.cpp) will be used.
- if `mmffDef` is supplied, a new singleton will be instantiated. The current instantiation (if there is one) will be deleted.

Referenced by ForceFields::MMFF::MMFFTorCollection::getMMFFTorParams(), ForceFields::MMFF::MMFFOopCollection::operator()(), and ForceFields::MMFF::MMFFAngleCollection::operator()().

|  |  |  |  |  |  |
| --- | --- | --- | --- | --- | --- |
| const MMFFDef\* ForceFields::MMFF::MMFFDefCollection::operator() | ( | const unsigned int | *atomType* | ) | const `[inline]` |

Looks up the parameters for a particular key and returns them.

**Returns:**
:   a pointer to the MMFFDef object, NULL on failure.

Definition at line 190 of file Params.h.

---

The documentation for this class was generated from the following file:

- Params.h

---

Generated on 16 Feb 2014 for RDKit-MMFF by 
 1.6.1 
