# Supplementary material for: Bringing the MMFF force field to the RDKit: implementation and validation
Source: J Cheminform. 2014 Jul 12;6:37. doi: 10.1186/s13321-014-0037-3 (PMC4116604; doi:10.1186/s13321-014-0037-3)
Supplement: Additional file 3: — Documentation. The file docs.zip expands to an HTML tree which documents the MMFF-related C++ and Python RDKit APIs; the documentation can be browsed opening the docs.html file in any HTML browser. The full RDKit documentation can be found at http://www.rdkit.org. [file s13321-014-0037-3-S3.zip › docs/cpp/classForceFields_1_1MMFF_1_1MMFFProp.html]

RDKit-MMFF: ForceFields::MMFF::MMFFProp Class Reference


- Main Page
- Namespaces
- Classes
- Files
- Directories

- Class List
- Class Members

ForceFields::MMFF::MMFFProp

# ForceFields::MMFF::MMFFProp Class Reference

class to store MMFF Properties
More...

`#include <Params.h>`

List of all members.

|  |  |
| --- | --- |
| Public Attributes | |
| boost::uint8\_t | atno |
| boost::uint8\_t | crd |
| boost::uint8\_t | val |
| boost::uint8\_t | pilp |
| boost::uint8\_t | mltb |
| boost::uint8\_t | arom |
| boost::uint8\_t | linh |
| boost::uint8\_t | sbmb |

---

## Detailed Description

class to store MMFF Properties

Definition at line 51 of file Params.h.

---

## Member Data Documentation

|  |
| --- |
| boost::uint8\_t ForceFields::MMFF::MMFFProp::arom |

Definition at line 58 of file Params.h.

|  |
| --- |
| boost::uint8\_t ForceFields::MMFF::MMFFProp::atno |

Definition at line 53 of file Params.h.

|  |
| --- |
| boost::uint8\_t ForceFields::MMFF::MMFFProp::crd |

Definition at line 54 of file Params.h.

|  |
| --- |
| boost::uint8\_t ForceFields::MMFF::MMFFProp::linh |

Definition at line 59 of file Params.h.

|  |
| --- |
| boost::uint8\_t ForceFields::MMFF::MMFFProp::mltb |

Definition at line 57 of file Params.h.

|  |
| --- |
| boost::uint8\_t ForceFields::MMFF::MMFFProp::pilp |

Definition at line 56 of file Params.h.

|  |
| --- |
| boost::uint8\_t ForceFields::MMFF::MMFFProp::sbmb |

Definition at line 60 of file Params.h.

|  |
| --- |
| boost::uint8\_t ForceFields::MMFF::MMFFProp::val |

Definition at line 55 of file Params.h.

---

The documentation for this class was generated from the following file:

- Params.h

---

Generated on 16 Feb 2014 for RDKit-MMFF by 
 1.6.1 
