# Supplementary material for: Bringing the MMFF force field to the RDKit: implementation and validation
Source: J Cheminform. 2014 Jul 12;6:37. doi: 10.1186/s13321-014-0037-3 (PMC4116604; doi:10.1186/s13321-014-0037-3)
Supplement: Additional file 3: — Documentation. The file docs.zip expands to an HTML tree which documents the MMFF-related C++ and Python RDKit APIs; the documentation can be browsed opening the docs.html file in any HTML browser. The full RDKit documentation can be found at http://www.rdkit.org. [file s13321-014-0037-3-S3.zip › docs/cpp/classForceFields_1_1MMFF_1_1MMFFTor.html]

RDKit-MMFF: ForceFields::MMFF::MMFFTor Class Reference


- Main Page
- Namespaces
- Classes
- Files
- Directories

- Class List
- Class Members

ForceFields::MMFF::MMFFTor

# ForceFields::MMFF::MMFFTor Class Reference

class to store MMFF parameters for torsions
More...

`#include <Params.h>`

List of all members.

|  |  |
| --- | --- |
| Public Attributes | |
| double | V1 |
| double | V2 |
| double | V3 |

---

## Detailed Description

class to store MMFF parameters for torsions

Definition at line 113 of file Params.h.

---

## Member Data Documentation

|  |
| --- |
| double ForceFields::MMFF::MMFFTor::V1 |

Definition at line 115 of file Params.h.

|  |
| --- |
| double ForceFields::MMFF::MMFFTor::V2 |

Definition at line 116 of file Params.h.

|  |
| --- |
| double ForceFields::MMFF::MMFFTor::V3 |

Definition at line 117 of file Params.h.

---

The documentation for this class was generated from the following file:

- Params.h

---

Generated on 16 Feb 2014 for RDKit-MMFF by 
 1.6.1 
