# Supplementary material for: Bringing the MMFF force field to the RDKit: implementation and validation
Source: J Cheminform. 2014 Jul 12;6:37. doi: 10.1186/s13321-014-0037-3 (PMC4116604; doi:10.1186/s13321-014-0037-3)
Supplement: Additional file 3: — Documentation. The file docs.zip expands to an HTML tree which documents the MMFF-related C++ and Python RDKit APIs; the documentation can be browsed opening the docs.html file in any HTML browser. The full RDKit documentation can be found at http://www.rdkit.org. [file s13321-014-0037-3-S3.zip › docs/cpp/classForceFields_1_1MMFF_1_1MMFFVdW-members.html]

RDKit-MMFF: Member List


- Main Page
- Namespaces
- Classes
- Files
- Directories

- Class List
- Class Members

# ForceFields::MMFF::MMFFVdW Member List

This is the complete list of members for ForceFields::MMFF::MMFFVdW, including all inherited members.

|  |  |  |
| --- | --- | --- |
| A\_i | ForceFields::MMFF::MMFFVdW |  |
| alpha\_i | ForceFields::MMFF::MMFFVdW |  |
| DA | ForceFields::MMFF::MMFFVdW |  |
| G\_i | ForceFields::MMFF::MMFFVdW |  |
| N\_i | ForceFields::MMFF::MMFFVdW |  |
| R\_star | ForceFields::MMFF::MMFFVdW |  |

---

Generated on 16 Feb 2014 for RDKit-MMFF by 
 1.6.1 
