# Supplementary material for: Bringing the MMFF force field to the RDKit: implementation and validation
Source: J Cheminform. 2014 Jul 12;6:37. doi: 10.1186/s13321-014-0037-3 (PMC4116604; doi:10.1186/s13321-014-0037-3)
Supplement: Additional file 3: — Documentation. The file docs.zip expands to an HTML tree which documents the MMFF-related C++ and Python RDKit APIs; the documentation can be browsed opening the docs.html file in any HTML browser. The full RDKit documentation can be found at http://www.rdkit.org. [file s13321-014-0037-3-S3.zip › docs/cpp/classForceFields_1_1MMFF_1_1StretchBendContrib.html]

RDKit-MMFF: ForceFields::MMFF::StretchBendContrib Class Reference


- Main Page
- Namespaces
- Classes
- Files
- Directories

- Class List
- Class Members

ForceFields::MMFF::StretchBendContrib

# ForceFields::MMFF::StretchBendContrib Class Reference

The angle-bend term for MMFF.
More...

`#include <StretchBend.h>`

List of all members.

|  |  |
| --- | --- |
| Public Member Functions | |
|  | StretchBendContrib () |
|  | StretchBendContrib (ForceField \*owner, const unsigned int idx1, const unsigned int idx2, const unsigned int idx3, const std::pair< bool, const MMFFStbn \* > mmffStbnParams, const MMFFAngle \*mmffAngleParams, const MMFFBond \*mmffBondParams1, const MMFFBond \*mmffBondParams2) |
|  | Constructor. |
| double | getEnergy (double \*pos) const |
| void | getGrad (double \*pos, double \*grad) const |

---

## Detailed Description

The angle-bend term for MMFF.

Definition at line 26 of file StretchBend.h.

---

## Constructor & Destructor Documentation

|  |  |  |  |  |
| --- | --- | --- | --- | --- |
| ForceFields::MMFF::StretchBendContrib::StretchBendContrib | ( |  | ) | `[inline]` |

Definition at line 28 of file StretchBend.h.

|  |  |  |  |
| --- | --- | --- | --- |
| ForceFields::MMFF::StretchBendContrib::StretchBendContrib | ( | ForceField \* | *owner*, |
|  |  | const unsigned int | *idx1*, |
|  |  | const unsigned int | *idx2*, |
|  |  | const unsigned int | *idx3*, |
|  |  | const std::pair< bool, const MMFFStbn \* > | *mmffStbnParams*, |
|  |  | const MMFFAngle \* | *mmffAngleParams*, |
|  |  | const MMFFBond \* | *mmffBondParams1*, |
|  |  | const MMFFBond \* | *mmffBondParams2* |  |
|  | ) |  |  |  |

Constructor.

The angle is between atom1 - atom2 - atom3

**Parameters:**
:   |  |  |  |
    | --- | --- | --- |
    |  | *owner* | pointer to the owning ForceField |
    |  | *idx1* | index of atom1 in the ForceField's positions |
    |  | *idx2* | index of atom2 in the ForceField's positions |
    |  | *idx3* | index of atom3 in the ForceField's positions |
    |  | *angleType* | MMFF type of the angle (as an unsigned int) |

---

## Member Function Documentation

|  |  |  |  |  |  |
| --- | --- | --- | --- | --- | --- |
| double ForceFields::MMFF::StretchBendContrib::getEnergy | ( | double \* | *pos* | ) | const |

|  |  |  |  |
| --- | --- | --- | --- |
| void ForceFields::MMFF::StretchBendContrib::getGrad | ( | double \* | *pos*, |
|  |  | double \* | *grad* |  |
|  | ) |  |  | const |

---

The documentation for this class was generated from the following file:

- StretchBend.h

---

Generated on 16 Feb 2014 for RDKit-MMFF by 
 1.6.1 
