# Supplementary material for: Bringing the MMFF force field to the RDKit: implementation and validation
Source: J Cheminform. 2014 Jul 12;6:37. doi: 10.1186/s13321-014-0037-3 (PMC4116604; doi:10.1186/s13321-014-0037-3)
Supplement: Additional file 3: — Documentation. The file docs.zip expands to an HTML tree which documents the MMFF-related C++ and Python RDKit APIs; the documentation can be browsed opening the docs.html file in any HTML browser. The full RDKit documentation can be found at http://www.rdkit.org. [file s13321-014-0037-3-S3.zip › docs/cpp/classForceFields_1_1MMFF_1_1VdWContrib.html]

RDKit-MMFF: ForceFields::MMFF::VdWContrib Class Reference


- Main Page
- Namespaces
- Classes
- Files
- Directories

- Class List
- Class Members

ForceFields::MMFF::VdWContrib

# ForceFields::MMFF::VdWContrib Class Reference

the van der Waals term for MMFF
More...

`#include <Nonbonded.h>`

List of all members.

|  |  |
| --- | --- |
| Public Member Functions | |
|  | VdWContrib () |
|  | VdWContrib (ForceField \*owner, unsigned int idx1, unsigned int idx2, MMFFVdWCollection \*mmffVdW, const MMFFVdW \*mmffVdWParamsAtom1, const MMFFVdW \*mmffVdWParamsAtom2) |
|  | Constructor. |
| double | getEnergy (double \*pos) const |
| void | getGrad (double \*pos, double \*grad) const |

---

## Detailed Description

the van der Waals term for MMFF

Definition at line 23 of file Nonbonded.h.

---

## Constructor & Destructor Documentation

|  |  |  |  |  |
| --- | --- | --- | --- | --- |
| ForceFields::MMFF::VdWContrib::VdWContrib | ( |  | ) | `[inline]` |

Definition at line 25 of file Nonbonded.h.

|  |  |  |  |
| --- | --- | --- | --- |
| ForceFields::MMFF::VdWContrib::VdWContrib | ( | ForceField \* | *owner*, |
|  |  | unsigned int | *idx1*, |
|  |  | unsigned int | *idx2*, |
|  |  | MMFFVdWCollection \* | *mmffVdW*, |
|  |  | const MMFFVdW \* | *mmffVdWParamsAtom1*, |
|  |  | const MMFFVdW \* | *mmffVdWParamsAtom2* |  |
|  | ) |  |  |  |

Constructor.

**Parameters:**
:   |  |  |  |
    | --- | --- | --- |
    |  | *owner* | pointer to the owning ForceField |
    |  | *idx1* | index of end1 in the ForceField's positions |
    |  | *idx2* | index of end2 in the ForceField's positions |

---

## Member Function Documentation

|  |  |  |  |  |  |
| --- | --- | --- | --- | --- | --- |
| double ForceFields::MMFF::VdWContrib::getEnergy | ( | double \* | *pos* | ) | const |

|  |  |  |  |
| --- | --- | --- | --- |
| void ForceFields::MMFF::VdWContrib::getGrad | ( | double \* | *pos*, |
|  |  | double \* | *grad* |  |
|  | ) |  |  | const |

---

The documentation for this class was generated from the following file:

- Nonbonded.h

---

Generated on 16 Feb 2014 for RDKit-MMFF by 
 1.6.1 
