# Supplementary material for: Bringing the MMFF force field to the RDKit: implementation and validation
Source: J Cheminform. 2014 Jul 12;6:37. doi: 10.1186/s13321-014-0037-3 (PMC4116604; doi:10.1186/s13321-014-0037-3)
Supplement: Additional file 3: — Documentation. The file docs.zip expands to an HTML tree which documents the MMFF-related C++ and Python RDKit APIs; the documentation can be browsed opening the docs.html file in any HTML browser. The full RDKit documentation can be found at http://www.rdkit.org. [file s13321-014-0037-3-S3.zip › docs/cpp/classRDKit_1_1MMFF_1_1MMFFAtomProperties.html]

RDKit-MMFF: RDKit::MMFF::MMFFAtomProperties Class Reference


- Main Page
- Namespaces
- Classes
- Files
- Directories

- Class List
- Class Members

RDKit::MMFF::MMFFAtomProperties

# RDKit::MMFF::MMFFAtomProperties Class Reference

`#include <AtomTyper.h>`

List of all members.

|  |  |
| --- | --- |
| Public Member Functions | |
|  | MMFFAtomProperties () |
|  | ~MMFFAtomProperties () |
| Public Attributes | |
| boost::uint8\_t | mmffAtomType |
| double | mmffFormalCharge |
| double | mmffPartialCharge |

---

## Detailed Description

Definition at line 29 of file AtomTyper.h.

---

## Constructor & Destructor Documentation

|  |  |  |  |  |
| --- | --- | --- | --- | --- |
| RDKit::MMFF::MMFFAtomProperties::MMFFAtomProperties | ( |  | ) | `[inline]` |

Definition at line 31 of file AtomTyper.h.

|  |  |  |  |  |
| --- | --- | --- | --- | --- |
| RDKit::MMFF::MMFFAtomProperties::~MMFFAtomProperties | ( |  | ) | `[inline]` |

Definition at line 35 of file AtomTyper.h.

---

## Member Data Documentation

|  |
| --- |
| boost::uint8\_t RDKit::MMFF::MMFFAtomProperties::mmffAtomType |

Definition at line 35 of file AtomTyper.h.

|  |
| --- |
| double RDKit::MMFF::MMFFAtomProperties::mmffFormalCharge |

Definition at line 37 of file AtomTyper.h.

|  |
| --- |
| double RDKit::MMFF::MMFFAtomProperties::mmffPartialCharge |

Definition at line 38 of file AtomTyper.h.

---

The documentation for this class was generated from the following file:

- AtomTyper.h

---

Generated on 16 Feb 2014 for RDKit-MMFF by 
 1.6.1 
