# Supplementary material for: Bringing the MMFF force field to the RDKit: implementation and validation
Source: J Cheminform. 2014 Jul 12;6:37. doi: 10.1186/s13321-014-0037-3 (PMC4116604; doi:10.1186/s13321-014-0037-3)
Supplement: Additional file 3: — Documentation. The file docs.zip expands to an HTML tree which documents the MMFF-related C++ and Python RDKit APIs; the documentation can be browsed opening the docs.html file in any HTML browser. The full RDKit documentation can be found at http://www.rdkit.org. [file s13321-014-0037-3-S3.zip › docs/cpp/classRDKit_1_1MMFF_1_1MMFFMolProperties.html]

RDKit-MMFF: RDKit::MMFF::MMFFMolProperties Class Reference


- Main Page
- Namespaces
- Classes
- Files
- Directories

- Class List
- Class Members

RDKit::MMFF::MMFFMolProperties

# RDKit::MMFF::MMFFMolProperties Class Reference

`#include <AtomTyper.h>`

List of all members.

|  |  |
| --- | --- |
| Public Member Functions | |
|  | MMFFMolProperties (ROMol &mol, std::string mmffVariant="MMFF94", boost::uint8\_t verbosity=MMFF\_VERBOSITY\_NONE, std::ostream &oStream=std::cout) |
|  | ~MMFFMolProperties () |
| const unsigned int | getMMFFBondType (const Bond \*bond) |
| const unsigned int | getMMFFAngleType (const ROMol &mol, const unsigned int idx1, const unsigned int idx2, const unsigned int idx3) |
| const std::pair< unsigned int,   unsigned int > | getMMFFTorsionType (const ROMol &mol, const unsigned int idx1, const unsigned int idx2, const unsigned int idx3, const unsigned int idx4) |
| void | computeMMFFCharges (const ROMol &mol) |
| const ForceFields::MMFF::MMFFTor \* | getMMFFTorsionEmpiricalRuleParams (const ROMol &mol, unsigned int idx2, unsigned int idx3) |
| const ForceFields::MMFF::MMFFBond \* | getMMFFBondStretchEmpiricalRuleParams (const ROMol &mol, const Bond \*bond) |
| const boost::uint8\_t | getMMFFAtomType (const unsigned int idx) |
| const double | getMMFFFormalCharge (const unsigned int idx) |
| const double | getMMFFPartialCharge (const unsigned int idx) |
| void | setMMFFBondTerm (const bool state) |
| const bool | getMMFFBondTerm () |
| void | setMMFFAngleTerm (const bool state) |
| const bool | getMMFFAngleTerm () |
| void | setMMFFStretchBendTerm (const bool state) |
| const bool | getMMFFStretchBendTerm () |
| void | setMMFFOopTerm (const bool state) |
| const bool | getMMFFOopTerm () |
| void | setMMFFTorsionTerm (const bool state) |
| const bool | getMMFFTorsionTerm () |
| void | setMMFFVdWTerm (const bool state) |
| const bool | getMMFFVdWTerm () |
| void | setMMFFEleTerm (const bool state) |
| const bool | getMMFFEleTerm () |
| void | setMMFFVariant (const std::string mmffVariant) |
| const std::string | getMMFFVariant () |
| void | setMMFFDielectricConstant (const double dielConst) |
| double | getMMFFDielectricConstant () |
| void | setMMFFDielectricModel (boost::uint8\_t dielModel) |
| boost::uint8\_t | getMMFFDielectricModel () |
| void | setMMFFVerbosity (boost::uint8\_t verbosity) |
| boost::uint8\_t | getMMFFVerbosity () |
| void | setMMFFOStream (std::ostream \*oStream) |
| std::ostream & | getMMFFOStream () |
| bool | isValid () |

---

## Detailed Description

Definition at line 51 of file AtomTyper.h.

---

## Constructor & Destructor Documentation

|  |  |  |  |
| --- | --- | --- | --- |
| RDKit::MMFF::MMFFMolProperties::MMFFMolProperties | ( | ROMol & | *mol*, |
|  |  | std::string | *mmffVariant* = `"MMFF94"`, |
|  |  | boost::uint8\_t | *verbosity* = `MMFF_VERBOSITY_NONE`, |
|  |  | std::ostream & | *oStream* = `std::cout` |  |
|  | ) |  |  |  |

|  |  |  |  |  |
| --- | --- | --- | --- | --- |
| RDKit::MMFF::MMFFMolProperties::~MMFFMolProperties | ( |  | ) | `[inline]` |

Definition at line 56 of file AtomTyper.h.

---

## Member Function Documentation

|  |  |  |  |  |  |
| --- | --- | --- | --- | --- | --- |
| void RDKit::MMFF::MMFFMolProperties::computeMMFFCharges | ( | const ROMol & | *mol* | ) |  |

|  |  |  |  |  |
| --- | --- | --- | --- | --- |
| const bool RDKit::MMFF::MMFFMolProperties::getMMFFAngleTerm | ( |  | ) | `[inline]` |

Definition at line 99 of file AtomTyper.h.

|  |  |  |  |
| --- | --- | --- | --- |
| const unsigned int RDKit::MMFF::MMFFMolProperties::getMMFFAngleType | ( | const ROMol & | *mol*, |
|  |  | const unsigned int | *idx1*, |
|  |  | const unsigned int | *idx2*, |
|  |  | const unsigned int | *idx3* |  |
|  | ) |  |  |  |

|  |  |  |  |  |  |
| --- | --- | --- | --- | --- | --- |
| const boost::uint8\_t RDKit::MMFF::MMFFMolProperties::getMMFFAtomType | ( | const unsigned int | *idx* | ) | `[inline]` |

Definition at line 69 of file AtomTyper.h.

|  |  |  |  |
| --- | --- | --- | --- |
| const ForceFields::MMFF::MMFFBond\* RDKit::MMFF::MMFFMolProperties::getMMFFBondStretchEmpiricalRuleParams | ( | const ROMol & | *mol*, |
|  |  | const Bond \* | *bond* |  |
|  | ) |  |  |  |

|  |  |  |  |  |
| --- | --- | --- | --- | --- |
| const bool RDKit::MMFF::MMFFMolProperties::getMMFFBondTerm | ( |  | ) | `[inline]` |

Definition at line 91 of file AtomTyper.h.

|  |  |  |  |  |  |
| --- | --- | --- | --- | --- | --- |
| const unsigned int RDKit::MMFF::MMFFMolProperties::getMMFFBondType | ( | const Bond \* | *bond* | ) |  |

|  |  |  |  |  |
| --- | --- | --- | --- | --- |
| double RDKit::MMFF::MMFFMolProperties::getMMFFDielectricConstant | ( |  | ) | `[inline]` |

Definition at line 160 of file AtomTyper.h.

|  |  |  |  |  |
| --- | --- | --- | --- | --- |
| boost::uint8\_t RDKit::MMFF::MMFFMolProperties::getMMFFDielectricModel | ( |  | ) | `[inline]` |

Definition at line 168 of file AtomTyper.h.

|  |  |  |  |  |
| --- | --- | --- | --- | --- |
| const bool RDKit::MMFF::MMFFMolProperties::getMMFFEleTerm | ( |  | ) | `[inline]` |

Definition at line 139 of file AtomTyper.h.

|  |  |  |  |  |  |
| --- | --- | --- | --- | --- | --- |
| const double RDKit::MMFF::MMFFMolProperties::getMMFFFormalCharge | ( | const unsigned int | *idx* | ) | `[inline]` |

Definition at line 75 of file AtomTyper.h.

|  |  |  |  |  |
| --- | --- | --- | --- | --- |
| const bool RDKit::MMFF::MMFFMolProperties::getMMFFOopTerm | ( |  | ) | `[inline]` |

Definition at line 115 of file AtomTyper.h.

|  |  |  |  |  |
| --- | --- | --- | --- | --- |
| std::ostream& RDKit::MMFF::MMFFMolProperties::getMMFFOStream | ( |  | ) | `[inline]` |

Definition at line 184 of file AtomTyper.h.

|  |  |  |  |  |  |
| --- | --- | --- | --- | --- | --- |
| const double RDKit::MMFF::MMFFMolProperties::getMMFFPartialCharge | ( | const unsigned int | *idx* | ) | `[inline]` |

Definition at line 81 of file AtomTyper.h.

|  |  |  |  |  |
| --- | --- | --- | --- | --- |
| const bool RDKit::MMFF::MMFFMolProperties::getMMFFStretchBendTerm | ( |  | ) | `[inline]` |

Definition at line 107 of file AtomTyper.h.

|  |  |  |  |
| --- | --- | --- | --- |
| const ForceFields::MMFF::MMFFTor\* RDKit::MMFF::MMFFMolProperties::getMMFFTorsionEmpiricalRuleParams | ( | const ROMol & | *mol*, |
|  |  | unsigned int | *idx2*, |
|  |  | unsigned int | *idx3* |  |
|  | ) |  |  |  |

|  |  |  |  |  |
| --- | --- | --- | --- | --- |
| const bool RDKit::MMFF::MMFFMolProperties::getMMFFTorsionTerm | ( |  | ) | `[inline]` |

Definition at line 123 of file AtomTyper.h.

|  |  |  |  |
| --- | --- | --- | --- |
| const std::pair<unsigned int, unsigned int> RDKit::MMFF::MMFFMolProperties::getMMFFTorsionType | ( | const ROMol & | *mol*, |
|  |  | const unsigned int | *idx1*, |
|  |  | const unsigned int | *idx2*, |
|  |  | const unsigned int | *idx3*, |
|  |  | const unsigned int | *idx4* |  |
|  | ) |  |  |  |

|  |  |  |  |  |
| --- | --- | --- | --- | --- |
| const std::string RDKit::MMFF::MMFFMolProperties::getMMFFVariant | ( |  | ) | `[inline]` |

Definition at line 150 of file AtomTyper.h.

|  |  |  |  |  |
| --- | --- | --- | --- | --- |
| const bool RDKit::MMFF::MMFFMolProperties::getMMFFVdWTerm | ( |  | ) | `[inline]` |

Definition at line 131 of file AtomTyper.h.

|  |  |  |  |  |
| --- | --- | --- | --- | --- |
| boost::uint8\_t RDKit::MMFF::MMFFMolProperties::getMMFFVerbosity | ( |  | ) | `[inline]` |

Definition at line 176 of file AtomTyper.h.

|  |  |  |  |  |
| --- | --- | --- | --- | --- |
| bool RDKit::MMFF::MMFFMolProperties::isValid | ( |  | ) | `[inline]` |

Definition at line 188 of file AtomTyper.h.

|  |  |  |  |  |  |
| --- | --- | --- | --- | --- | --- |
| void RDKit::MMFF::MMFFMolProperties::setMMFFAngleTerm | ( | const bool | *state* | ) | `[inline]` |

Definition at line 95 of file AtomTyper.h.

|  |  |  |  |  |  |
| --- | --- | --- | --- | --- | --- |
| void RDKit::MMFF::MMFFMolProperties::setMMFFBondTerm | ( | const bool | *state* | ) | `[inline]` |

Definition at line 87 of file AtomTyper.h.

|  |  |  |  |  |  |
| --- | --- | --- | --- | --- | --- |
| void RDKit::MMFF::MMFFMolProperties::setMMFFDielectricConstant | ( | const double | *dielConst* | ) | `[inline]` |

Definition at line 154 of file AtomTyper.h.

|  |  |  |  |  |  |
| --- | --- | --- | --- | --- | --- |
| void RDKit::MMFF::MMFFMolProperties::setMMFFDielectricModel | ( | boost::uint8\_t | *dielModel* | ) | `[inline]` |

Definition at line 164 of file AtomTyper.h.

|  |  |  |  |  |  |
| --- | --- | --- | --- | --- | --- |
| void RDKit::MMFF::MMFFMolProperties::setMMFFEleTerm | ( | const bool | *state* | ) | `[inline]` |

Definition at line 135 of file AtomTyper.h.

|  |  |  |  |  |  |
| --- | --- | --- | --- | --- | --- |
| void RDKit::MMFF::MMFFMolProperties::setMMFFOopTerm | ( | const bool | *state* | ) | `[inline]` |

Definition at line 111 of file AtomTyper.h.

|  |  |  |  |  |  |
| --- | --- | --- | --- | --- | --- |
| void RDKit::MMFF::MMFFMolProperties::setMMFFOStream | ( | std::ostream \* | *oStream* | ) | `[inline]` |

Definition at line 180 of file AtomTyper.h.

|  |  |  |  |  |  |
| --- | --- | --- | --- | --- | --- |
| void RDKit::MMFF::MMFFMolProperties::setMMFFStretchBendTerm | ( | const bool | *state* | ) | `[inline]` |

Definition at line 103 of file AtomTyper.h.

|  |  |  |  |  |  |
| --- | --- | --- | --- | --- | --- |
| void RDKit::MMFF::MMFFMolProperties::setMMFFTorsionTerm | ( | const bool | *state* | ) | `[inline]` |

Definition at line 119 of file AtomTyper.h.

|  |  |  |  |  |  |
| --- | --- | --- | --- | --- | --- |
| void RDKit::MMFF::MMFFMolProperties::setMMFFVariant | ( | const std::string | *mmffVariant* | ) | `[inline]` |

Definition at line 143 of file AtomTyper.h.

|  |  |  |  |  |  |
| --- | --- | --- | --- | --- | --- |
| void RDKit::MMFF::MMFFMolProperties::setMMFFVdWTerm | ( | const bool | *state* | ) | `[inline]` |

Definition at line 127 of file AtomTyper.h.

|  |  |  |  |  |  |
| --- | --- | --- | --- | --- | --- |
| void RDKit::MMFF::MMFFMolProperties::setMMFFVerbosity | ( | boost::uint8\_t | *verbosity* | ) | `[inline]` |

Definition at line 172 of file AtomTyper.h.

---

The documentation for this class was generated from the following file:

- AtomTyper.h

---

Generated on 16 Feb 2014 for RDKit-MMFF by 
 1.6.1 
