# Supplementary material for: Bringing the MMFF force field to the RDKit: implementation and validation
Source: J Cheminform. 2014 Jul 12;6:37. doi: 10.1186/s13321-014-0037-3 (PMC4116604; doi:10.1186/s13321-014-0037-3)
Supplement: Additional file 3: — Documentation. The file docs.zip expands to an HTML tree which documents the MMFF-related C++ and Python RDKit APIs; the documentation can be browsed opening the docs.html file in any HTML browser. The full RDKit documentation can be found at http://www.rdkit.org. [file s13321-014-0037-3-S3.zip › docs/cpp/classRDKit_1_1MMFF_1_1MMFFMolProperties-members.html]

RDKit-MMFF: Member List


- Main Page
- Namespaces
- Classes
- Files
- Directories

- Class List
- Class Members

# RDKit::MMFF::MMFFMolProperties Member List

This is the complete list of members for RDKit::MMFF::MMFFMolProperties, including all inherited members.

|  |  |  |
| --- | --- | --- |
| computeMMFFCharges(const ROMol &mol) | RDKit::MMFF::MMFFMolProperties |  |
| getMMFFAngleTerm() | RDKit::MMFF::MMFFMolProperties | `[inline]` |
| getMMFFAngleType(const ROMol &mol, const unsigned int idx1, const unsigned int idx2, const unsigned int idx3) | RDKit::MMFF::MMFFMolProperties |  |
| getMMFFAtomType(const unsigned int idx) | RDKit::MMFF::MMFFMolProperties | `[inline]` |
| getMMFFBondStretchEmpiricalRuleParams(const ROMol &mol, const Bond \*bond) | RDKit::MMFF::MMFFMolProperties |  |
| getMMFFBondTerm() | RDKit::MMFF::MMFFMolProperties | `[inline]` |
| getMMFFBondType(const Bond \*bond) | RDKit::MMFF::MMFFMolProperties |  |
| getMMFFDielectricConstant() | RDKit::MMFF::MMFFMolProperties | `[inline]` |
| getMMFFDielectricModel() | RDKit::MMFF::MMFFMolProperties | `[inline]` |
| getMMFFEleTerm() | RDKit::MMFF::MMFFMolProperties | `[inline]` |
| getMMFFFormalCharge(const unsigned int idx) | RDKit::MMFF::MMFFMolProperties | `[inline]` |
| getMMFFOopTerm() | RDKit::MMFF::MMFFMolProperties | `[inline]` |
| getMMFFOStream() | RDKit::MMFF::MMFFMolProperties | `[inline]` |
| getMMFFPartialCharge(const unsigned int idx) | RDKit::MMFF::MMFFMolProperties | `[inline]` |
| getMMFFStretchBendTerm() | RDKit::MMFF::MMFFMolProperties | `[inline]` |
| getMMFFTorsionEmpiricalRuleParams(const ROMol &mol, unsigned int idx2, unsigned int idx3) | RDKit::MMFF::MMFFMolProperties |  |
| getMMFFTorsionTerm() | RDKit::MMFF::MMFFMolProperties | `[inline]` |
| getMMFFTorsionType(const ROMol &mol, const unsigned int idx1, const unsigned int idx2, const unsigned int idx3, const unsigned int idx4) | RDKit::MMFF::MMFFMolProperties |  |
| getMMFFVariant() | RDKit::MMFF::MMFFMolProperties | `[inline]` |
| getMMFFVdWTerm() | RDKit::MMFF::MMFFMolProperties | `[inline]` |
| getMMFFVerbosity() | RDKit::MMFF::MMFFMolProperties | `[inline]` |
| isValid() | RDKit::MMFF::MMFFMolProperties | `[inline]` |
| MMFFMolProperties(ROMol &mol, std::string mmffVariant="MMFF94", boost::uint8\_t verbosity=MMFF\_VERBOSITY\_NONE, std::ostream &oStream=std::cout) | RDKit::MMFF::MMFFMolProperties |  |
| setMMFFAngleTerm(const bool state) | RDKit::MMFF::MMFFMolProperties | `[inline]` |
| setMMFFBondTerm(const bool state) | RDKit::MMFF::MMFFMolProperties | `[inline]` |
| setMMFFDielectricConstant(const double dielConst) | RDKit::MMFF::MMFFMolProperties | `[inline]` |
| setMMFFDielectricModel(boost::uint8\_t dielModel) | RDKit::MMFF::MMFFMolProperties | `[inline]` |
| setMMFFEleTerm(const bool state) | RDKit::MMFF::MMFFMolProperties | `[inline]` |
| setMMFFOopTerm(const bool state) | RDKit::MMFF::MMFFMolProperties | `[inline]` |
| setMMFFOStream(std::ostream \*oStream) | RDKit::MMFF::MMFFMolProperties | `[inline]` |
| setMMFFStretchBendTerm(const bool state) | RDKit::MMFF::MMFFMolProperties | `[inline]` |
| setMMFFTorsionTerm(const bool state) | RDKit::MMFF::MMFFMolProperties | `[inline]` |
| setMMFFVariant(const std::string mmffVariant) | RDKit::MMFF::MMFFMolProperties | `[inline]` |
| setMMFFVdWTerm(const bool state) | RDKit::MMFF::MMFFMolProperties | `[inline]` |
| setMMFFVerbosity(boost::uint8\_t verbosity) | RDKit::MMFF::MMFFMolProperties | `[inline]` |
| ~MMFFMolProperties() | RDKit::MMFF::MMFFMolProperties | `[inline]` |

---

Generated on 16 Feb 2014 for RDKit-MMFF by 
 1.6.1 
