# Supplementary material for: Bringing the MMFF force field to the RDKit: implementation and validation
Source: J Cheminform. 2014 Jul 12;6:37. doi: 10.1186/s13321-014-0037-3 (PMC4116604; doi:10.1186/s13321-014-0037-3)
Supplement: Additional file 3: — Documentation. The file docs.zip expands to an HTML tree which documents the MMFF-related C++ and Python RDKit APIs; the documentation can be browsed opening the docs.html file in any HTML browser. The full RDKit documentation can be found at http://www.rdkit.org. [file s13321-014-0037-3-S3.zip › docs/cpp/Contribs_8h.html]

RDKit-MMFF: Contribs.h File Reference


- Main Page
- Namespaces
- Classes
- Files
- Directories

- File List
- File Members

ForceField » MMFF

# Contribs.h File Reference

This convenience include pulls in all the other MMFF include files.
More...

`#include "BondStretch.h"`  
`#include "AngleBend.h"`  
`#include "StretchBend.h"`  
`#include "OopBend.h"`  
`#include "TorsionAngle.h"`  
`#include "Nonbonded.h"`  

Go to the source code of this file.


---

## Detailed Description

This convenience include pulls in all the other MMFF include files.

Definition in file Contribs.h.

---

Generated on 16 Feb 2014 for RDKit-MMFF by 
 1.6.1 
