# Supplementary material for: Bringing the MMFF force field to the RDKit: implementation and validation
Source: J Cheminform. 2014 Jul 12;6:37. doi: 10.1186/s13321-014-0037-3 (PMC4116604; doi:10.1186/s13321-014-0037-3)
Supplement: Additional file 3: — Documentation. The file docs.zip expands to an HTML tree which documents the MMFF-related C++ and Python RDKit APIs; the documentation can be browsed opening the docs.html file in any HTML browser. The full RDKit documentation can be found at http://www.rdkit.org. [file s13321-014-0037-3-S3.zip › docs/cpp/Contribs_8h_source.html]

RDKit-MMFF: Contribs.h Source File


- Main Page
- Namespaces
- Classes
- Files
- Directories

- File List
- File Members

ForceField » MMFF

# Contribs.h

Go to the documentation of this file.

```
00001 //
00002 //  Copyright (C) 2013 Paolo Tosco
00003 //
00004 //  Copyright (C) 2004-2006 Rational Discovery LLC
00005 //
00006 //   @@ All Rights Reserved @@
00007 //  This file is part of the RDKit.
00008 //  The contents are covered by the terms of the BSD license
00009 //  which is included in the file license.txt, found at the root
00010 //  of the RDKit source tree.
00011 //
00012 #ifndef __RD_MMFFCONTRIBS_H__
00013 #define __RD_MMFFCONTRIBS_H__
00014 /*! \file Contribs.h
00015   \brief This convenience include pulls in all the other MMFF include files
00016 */
00017 
00018 #include "BondStretch.h"
00019 #include "AngleBend.h"
00020 #include "StretchBend.h"
00021 #include "OopBend.h"
00022 #include "TorsionAngle.h"
00023 #include "Nonbonded.h"
00024 
00025 #endif
```

---

Generated on 16 Feb 2014 for RDKit-MMFF by 
 1.6.1 
