# Supplementary material for: Bringing the MMFF force field to the RDKit: implementation and validation
Source: J Cheminform. 2014 Jul 12;6:37. doi: 10.1186/s13321-014-0037-3 (PMC4116604; doi:10.1186/s13321-014-0037-3)
Supplement: Additional file 3: — Documentation. The file docs.zip expands to an HTML tree which documents the MMFF-related C++ and Python RDKit APIs; the documentation can be browsed opening the docs.html file in any HTML browser. The full RDKit documentation can be found at http://www.rdkit.org. [file s13321-014-0037-3-S3.zip › docs/cpp/DistanceConstraint_8h_source.html]

RDKit-MMFF: DistanceConstraint.h Source File


- Main Page
- Namespaces
- Classes
- Files
- Directories

- File List
- File Members

ForceField » MMFF

# DistanceConstraint.h

Go to the documentation of this file.

```
00001 //
00002 //  Copyright (C) 2013 Paolo Tosco
00003 //
00004 //  Copyright (C) 2004-2006 Rational Discovery LLC
00005 //
00006 //   @@ All Rights Reserved @@
00007 //  This file is part of the RDKit.
00008 //  The contents are covered by the terms of the BSD license
00009 //  which is included in the file license.txt, found at the root
00010 //  of the RDKit source tree.
00011 //
00012 #ifndef __RD_MMFFDISTANCECONSTRAINT_H__
00013 #define __RD_MMFFDISTANCECONSTRAINT_H__
00014 #include <iostream>
00015 #include <ForceField/Contrib.h>
00016 
00017 namespace ForceFields {
00018   namespace MMFF {
00019 
00020     //! A distance range constraint modelled after a BondStretchContrib
00021     class DistanceConstraintContrib : public ForceFieldContrib {
00022     public:
00023       DistanceConstraintContrib() : d_end1Idx(-1), d_end2Idx(-1) {};
00024       //! Constructor
00025       /*!
00026         \param owner       pointer to the owning ForceField
00027         \param idx1        index of end1 in the ForceField's positions
00028         \param idx2        index of end2 in the ForceField's positions
00029         \param minLen      minimum distance
00030         \param maxLen      maximum distance
00031         \param forceConst  force Constant
00032         
00033       */
00034       DistanceConstraintContrib(ForceField *owner, unsigned int idx1, unsigned int idx2,
00035                                 double minLen, double maxLen, double forceConst);
00036       DistanceConstraintContrib(ForceField *owner, unsigned int idx1, unsigned int idx2,
00037                                 bool relative, double minLen, double maxLen, double forceConst);
00038 
00039       ~DistanceConstraintContrib() {
00040         //std::cerr << " ==== Destroy constraint " << d_end1Idx << " " << d_end2Idx << std::endl;
00041       }
00042       double getEnergy(double *pos) const;
00043 
00044       void getGrad(double *pos, double *grad) const;
00045     private:
00046       int d_end1Idx, d_end2Idx; //!< indices of end points
00047       double d_minLen, d_maxLen;        //!< rest length of the bond
00048       double d_forceConstant;  //!< force constant of the bond
00049 
00050     };
00051   }
00052 }
00053 #endif
```

---

Generated on 16 Feb 2014 for RDKit-MMFF by 
 1.6.1 
