# Supplementary material for: Bringing the MMFF force field to the RDKit: implementation and validation
Source: J Cheminform. 2014 Jul 12;6:37. doi: 10.1186/s13321-014-0037-3 (PMC4116604; doi:10.1186/s13321-014-0037-3)
Supplement: Additional file 3: — Documentation. The file docs.zip expands to an HTML tree which documents the MMFF-related C++ and Python RDKit APIs; the documentation can be browsed opening the docs.html file in any HTML browser. The full RDKit documentation can be found at http://www.rdkit.org. [file s13321-014-0037-3-S3.zip › docs/cpp/functions_func.html]

RDKit-MMFF: Class Members - Functions


- Main Page
- Namespaces
- Classes
- Files
- Directories

- Class List
- Class Members

- All
- Functions
- Variables

- a
- b
- c
- d
- e
- g
- i
- m
- o
- p
- s
- t
- v
- ~

### - a -

- AngleBendContrib()
  : ForceFields::MMFF::AngleBendContrib
- AngleConstraintContrib()
  : ForceFields::MMFF::AngleConstraintContrib

### - b -

- BondStretchContrib()
  : ForceFields::MMFF::BondStretchContrib

### - c -

- computeMMFFCharges()
  : RDKit::MMFF::MMFFMolProperties

### - d -

- DistanceConstraintContrib()
  : ForceFields::MMFF::DistanceConstraintContrib

### - e -

- EleContrib()
  : ForceFields::MMFF::EleContrib

### - g -

- getEnergy()
  : ForceFields::MMFF::AngleBendContrib
  , ForceFields::MMFF::AngleConstraintContrib
  , ForceFields::MMFF::DistanceConstraintContrib
  , ForceFields::MMFF::PositionConstraintContrib
  , ForceFields::MMFF::StretchBendContrib
  , ForceFields::MMFF::VdWContrib
  , ForceFields::MMFF::TorsionAngleContrib
  , ForceFields::MMFF::TorsionConstraintContrib
  , ForceFields::MMFF::BondStretchContrib
  , ForceFields::MMFF::EleContrib
  , ForceFields::MMFF::OopBendContrib
- getGrad()
  : ForceFields::MMFF::VdWContrib
  , ForceFields::MMFF::EleContrib
  , ForceFields::MMFF::OopBendContrib
  , ForceFields::MMFF::PositionConstraintContrib
  , ForceFields::MMFF::StretchBendContrib
  , ForceFields::MMFF::TorsionAngleContrib
  , ForceFields::MMFF::TorsionConstraintContrib
  , ForceFields::MMFF::AngleBendContrib
  , ForceFields::MMFF::AngleConstraintContrib
  , ForceFields::MMFF::BondStretchContrib
  , ForceFields::MMFF::DistanceConstraintContrib
- getMMFFAngle()
  : ForceFields::MMFF::MMFFAngleCollection
- getMMFFAngleTerm()
  : RDKit::MMFF::MMFFMolProperties
- getMMFFAngleType()
  : RDKit::MMFF::MMFFMolProperties
- getMMFFArom()
  : ForceFields::MMFF::MMFFAromCollection
- getMMFFAtomType()
  : RDKit::MMFF::MMFFMolProperties
- getMMFFBndk()
  : ForceFields::MMFF::MMFFBndkCollection
- getMMFFBond()
  : ForceFields::MMFF::MMFFBondCollection
- getMMFFBondStretchEmpiricalRuleParams()
  : RDKit::MMFF::MMFFMolProperties
- getMMFFBondTerm()
  : RDKit::MMFF::MMFFMolProperties
- getMMFFBondType()
  : RDKit::MMFF::MMFFMolProperties
- getMMFFChg()
  : ForceFields::MMFF::MMFFChgCollection
- getMMFFChgParams()
  : ForceFields::MMFF::MMFFChgCollection
- getMMFFCovRadPauEle()
  : ForceFields::MMFF::MMFFCovRadPauEleCollection
- getMMFFDef()
  : ForceFields::MMFF::MMFFDefCollection
- getMMFFDfsb()
  : ForceFields::MMFF::MMFFDfsbCollection
- getMMFFDfsbParams()
  : ForceFields::MMFF::MMFFDfsbCollection
- getMMFFDielectricConstant()
  : RDKit::MMFF::MMFFMolProperties
- getMMFFDielectricModel()
  : RDKit::MMFF::MMFFMolProperties
- getMMFFEleTerm()
  : RDKit::MMFF::MMFFMolProperties
- getMMFFFormalCharge()
  : RDKit::MMFF::MMFFMolProperties
- getMMFFOop()
  : ForceFields::MMFF::MMFFOopCollection
- getMMFFOopTerm()
  : RDKit::MMFF::MMFFMolProperties
- getMMFFOStream()
  : RDKit::MMFF::MMFFMolProperties
- getMMFFPartialCharge()
  : RDKit::MMFF::MMFFMolProperties
- getMMFFPBCI()
  : ForceFields::MMFF::MMFFPBCICollection
- getMMFFProp()
  : ForceFields::MMFF::MMFFPropCollection
- getMMFFStbn()
  : ForceFields::MMFF::MMFFStbnCollection
- getMMFFStbnParams()
  : ForceFields::MMFF::MMFFStbnCollection
- getMMFFStretchBendTerm()
  : RDKit::MMFF::MMFFMolProperties
- getMMFFTor()
  : ForceFields::MMFF::MMFFTorCollection
- getMMFFTorParams()
  : ForceFields::MMFF::MMFFTorCollection
- getMMFFTorsionEmpiricalRuleParams()
  : RDKit::MMFF::MMFFMolProperties
- getMMFFTorsionTerm()
  : RDKit::MMFF::MMFFMolProperties
- getMMFFTorsionType()
  : RDKit::MMFF::MMFFMolProperties
- getMMFFVariant()
  : RDKit::MMFF::MMFFMolProperties
- getMMFFVdW()
  : ForceFields::MMFF::MMFFVdWCollection
- getMMFFVdWTerm()
  : RDKit::MMFF::MMFFMolProperties
- getMMFFVerbosity()
  : RDKit::MMFF::MMFFMolProperties

### - i -

- isMMFFAromatic()
  : ForceFields::MMFF::MMFFAromCollection
- isValid()
  : RDKit::MMFF::MMFFMolProperties

### - m -

- MMFFAtomProperties()
  : RDKit::MMFF::MMFFAtomProperties
- MMFFMolProperties()
  : RDKit::MMFF::MMFFMolProperties

### - o -

- OopBendContrib()
  : ForceFields::MMFF::OopBendContrib
- operator()()
  : ForceFields::MMFF::MMFFOopCollection
  , ForceFields::MMFF::MMFFAngleCollection
  , ForceFields::MMFF::MMFFCovRadPauEleCollection
  , ForceFields::MMFF::MMFFBndkCollection
  , ForceFields::MMFF::MMFFBondCollection
  , ForceFields::MMFF::MMFFPBCICollection
  , ForceFields::MMFF::MMFFPropCollection
  , ForceFields::MMFF::MMFFDefCollection
  , ForceFields::MMFF::MMFFVdWCollection

### - p -

- PositionConstraintContrib()
  : ForceFields::MMFF::PositionConstraintContrib

### - s -

- setMMFFAngleTerm()
  : RDKit::MMFF::MMFFMolProperties
- setMMFFBondTerm()
  : RDKit::MMFF::MMFFMolProperties
- setMMFFDielectricConstant()
  : RDKit::MMFF::MMFFMolProperties
- setMMFFDielectricModel()
  : RDKit::MMFF::MMFFMolProperties
- setMMFFEleTerm()
  : RDKit::MMFF::MMFFMolProperties
- setMMFFOopTerm()
  : RDKit::MMFF::MMFFMolProperties
- setMMFFOStream()
  : RDKit::MMFF::MMFFMolProperties
- setMMFFStretchBendTerm()
  : RDKit::MMFF::MMFFMolProperties
- setMMFFTorsionTerm()
  : RDKit::MMFF::MMFFMolProperties
- setMMFFVariant()
  : RDKit::MMFF::MMFFMolProperties
- setMMFFVdWTerm()
  : RDKit::MMFF::MMFFMolProperties
- setMMFFVerbosity()
  : RDKit::MMFF::MMFFMolProperties
- StretchBendContrib()
  : ForceFields::MMFF::StretchBendContrib

### - t -

- TorsionAngleContrib()
  : ForceFields::MMFF::TorsionAngleContrib
- TorsionConstraintContrib()
  : ForceFields::MMFF::TorsionConstraintContrib

### - v -

- VdWContrib()
  : ForceFields::MMFF::VdWContrib

### - ~ -

- ~AngleConstraintContrib()
  : ForceFields::MMFF::AngleConstraintContrib
- ~DistanceConstraintContrib()
  : ForceFields::MMFF::DistanceConstraintContrib
- ~MMFFAtomProperties()
  : RDKit::MMFF::MMFFAtomProperties
- ~MMFFMolProperties()
  : RDKit::MMFF::MMFFMolProperties
- ~PositionConstraintContrib()
  : ForceFields::MMFF::PositionConstraintContrib
- ~TorsionConstraintContrib()
  : ForceFields::MMFF::TorsionConstraintContrib

---

Generated on 16 Feb 2014 for RDKit-MMFF by 
 1.6.1 
