# Supplementary material for: Bringing the MMFF force field to the RDKit: implementation and validation
Source: J Cheminform. 2014 Jul 12;6:37. doi: 10.1186/s13321-014-0037-3 (PMC4116604; doi:10.1186/s13321-014-0037-3)
Supplement: Additional file 3: — Documentation. The file docs.zip expands to an HTML tree which documents the MMFF-related C++ and Python RDKit APIs; the documentation can be browsed opening the docs.html file in any HTML browser. The full RDKit documentation can be found at http://www.rdkit.org. [file s13321-014-0037-3-S3.zip › docs/cpp/functions_vars.html]

RDKit-MMFF: Class Members - Variables


- Main Page
- Namespaces
- Classes
- Files
- Directories

- Class List
- Class Members

- All
- Functions
- Variables

- a
- b
- c
- d
- e
- f
- g
- k
- l
- m
- n
- p
- r
- s
- t
- v

### - a -

- A\_i
  : ForceFields::MMFF::MMFFVdW
- alpha\_i
  : ForceFields::MMFF::MMFFVdW
- arom
  : ForceFields::MMFF::MMFFProp
- atno
  : ForceFields::MMFF::MMFFProp

### - b -

- B
  : ForceFields::MMFF::MMFFVdWCollection
- bci
  : ForceFields::MMFF::MMFFChg
- Beta
  : ForceFields::MMFF::MMFFVdWCollection

### - c -

- chi
  : ForceFields::MMFF::MMFFCovRadPauEle
- crd
  : ForceFields::MMFF::MMFFProp

### - d -

- DA
  : ForceFields::MMFF::MMFFVdW
- DAEPS
  : ForceFields::MMFF::MMFFVdWCollection
- DARAD
  : ForceFields::MMFF::MMFFVdWCollection

### - e -

- eqLevel
  : ForceFields::MMFF::MMFFDef

### - f -

- fcadj
  : ForceFields::MMFF::MMFFPBCI

### - g -

- G\_i
  : ForceFields::MMFF::MMFFVdW

### - k -

- ka
  : ForceFields::MMFF::MMFFAngle
- kb
  : ForceFields::MMFF::MMFFBond
- kbaIJK
  : ForceFields::MMFF::MMFFStbn
- kbaKJI
  : ForceFields::MMFF::MMFFStbn
- koop
  : ForceFields::MMFF::MMFFOop

### - l -

- linh
  : ForceFields::MMFF::MMFFProp

### - m -

- mltb
  : ForceFields::MMFF::MMFFProp
- mmffAtomType
  : RDKit::MMFF::MMFFAtomProperties
- mmffFormalCharge
  : RDKit::MMFF::MMFFAtomProperties
- mmffPartialCharge
  : RDKit::MMFF::MMFFAtomProperties

### - n -

- N\_i
  : ForceFields::MMFF::MMFFVdW

### - p -

- pbci
  : ForceFields::MMFF::MMFFPBCI
- pilp
  : ForceFields::MMFF::MMFFProp
- power
  : ForceFields::MMFF::MMFFVdWCollection

### - r -

- r0
  : ForceFields::MMFF::MMFFBond
  , ForceFields::MMFF::MMFFCovRadPauEle
- R\_star
  : ForceFields::MMFF::MMFFVdW

### - s -

- sbmb
  : ForceFields::MMFF::MMFFProp

### - t -

- theta0
  : ForceFields::MMFF::MMFFAngle

### - v -

- V1
  : ForceFields::MMFF::MMFFTor
- V2
  : ForceFields::MMFF::MMFFTor
- V3
  : ForceFields::MMFF::MMFFTor
- val
  : ForceFields::MMFF::MMFFProp

---

Generated on 16 Feb 2014 for RDKit-MMFF by 
 1.6.1 
