# Supplementary material for: Bringing the MMFF force field to the RDKit: implementation and validation
Source: J Cheminform. 2014 Jul 12;6:37. doi: 10.1186/s13321-014-0037-3 (PMC4116604; doi:10.1186/s13321-014-0037-3)
Supplement: Additional file 3: — Documentation. The file docs.zip expands to an HTML tree which documents the MMFF-related C++ and Python RDKit APIs; the documentation can be browsed opening the docs.html file in any HTML browser. The full RDKit documentation can be found at http://www.rdkit.org. [file s13321-014-0037-3-S3.zip › docs/cpp/namespaceForceFields_1_1MMFF.html]

RDKit-MMFF: ForceFields::MMFF Namespace Reference


- Main Page
- Namespaces
- Classes
- Files
- Directories

- Namespace List
- Namespace Members

ForceFields::MMFF

# ForceFields::MMFF Namespace Reference

|  |  |
| --- | --- |
| Namespaces | |
| namespace | Utils |
| Classes | |
| class | AngleBendContrib |
|  | The angle-bend term for MMFF. More... |
| class | AngleConstraintContrib |
|  | An angle range constraint modelled after a AngleBendContrib. More... |
| class | BondStretchContrib |
|  | The bond-stretch term for MMFF. More... |
| class | DistanceConstraintContrib |
|  | A distance range constraint modelled after a BondStretchContrib. More... |
| class | VdWContrib |
|  | the van der Waals term for MMFF More... |
| class | EleContrib |
|  | the electrostatic term for MMFF More... |
| class | OopBendContrib |
|  | the out-of-plane term for MMFF More... |
| class | MMFFDef |
|  | class to store MMFF atom type equivalence levels More... |
| class | MMFFProp |
|  | class to store MMFF Properties More... |
| class | MMFFPBCI |
|  | class to store MMFF Partial Bond Charge Increments More... |
| class | MMFFChg |
| class | MMFFBond |
|  | class to store MMFF parameters for bond stretching More... |
| class | MMFFCovRadPauEle |
| class | MMFFAngle |
|  | class to store MMFF parameters for angle bending More... |
| class | MMFFStbn |
|  | class to store MMFF parameters for stretch-bending More... |
| class | MMFFOop |
|  | class to store MMFF parameters for out-of-plane bending More... |
| class | MMFFTor |
|  | class to store MMFF parameters for torsions More... |
| class | MMFFVdW |
|  | class to store MMFF parameters for non-bonded Van der Waals More... |
| class | MMFFAromCollection |
| class | MMFFDefCollection |
| class | MMFFPropCollection |
| class | MMFFPBCICollection |
| class | MMFFChgCollection |
| class | MMFFBondCollection |
| class | MMFFBndkCollection |
| class | MMFFCovRadPauEleCollection |
| class | MMFFAngleCollection |
| class | MMFFStbnCollection |
| class | MMFFDfsbCollection |
| class | MMFFOopCollection |
| class | MMFFTorCollection |
| class | MMFFVdWCollection |
| class | PositionConstraintContrib |
|  | A position constraint of the type 0.5k \* deltaX^2. More... |
| class | StretchBendContrib |
|  | The angle-bend term for MMFF. More... |
| class | TorsionAngleContrib |
|  | the torsion term for MMFF More... |
| class | TorsionConstraintContrib |
|  | A dihedral angle range constraint modelled after a TorsionContrib. More... |
| Functions | |
| const bool | isDoubleZero (const double x) |
| Variables | |
| const double | DEG2RAD = M\_PI / 180.0 |
| const double | RAD2DEG = 180.0 / M\_PI |

---

## Function Documentation

|  |  |  |  |  |  |
| --- | --- | --- | --- | --- | --- |
| const bool ForceFields::MMFF::isDoubleZero | ( | const double | *x* | ) | `[inline]` |

Definition at line 40 of file Params.h.

---

## Variable Documentation

|  |
| --- |
| const double ForceFields::MMFF::DEG2RAD = M\_PI / 180.0 |

Definition at line 38 of file Params.h.

|  |
| --- |
| const double ForceFields::MMFF::RAD2DEG = 180.0 / M\_PI |

Definition at line 39 of file Params.h.

---

Generated on 16 Feb 2014 for RDKit-MMFF by 
 1.6.1 
