# Supplementary material for: Bringing the MMFF force field to the RDKit: implementation and validation
Source: J Cheminform. 2014 Jul 12;6:37. doi: 10.1186/s13321-014-0037-3 (PMC4116604; doi:10.1186/s13321-014-0037-3)
Supplement: Additional file 3: — Documentation. The file docs.zip expands to an HTML tree which documents the MMFF-related C++ and Python RDKit APIs; the documentation can be browsed opening the docs.html file in any HTML browser. The full RDKit documentation can be found at http://www.rdkit.org. [file s13321-014-0037-3-S3.zip › docs/cpp/namespaceForceFields_1_1MMFF_1_1Utils.html]

RDKit-MMFF: ForceFields::MMFF::Utils Namespace Reference


- Main Page
- Namespaces
- Classes
- Files
- Directories

- Namespace List
- Namespace Members

ForceFields::MMFF::Utils

# ForceFields::MMFF::Utils Namespace Reference

|  |  |
| --- | --- |
| Functions | |
| double | calcAngleRestValue (const MMFFAngle \*mmffAngleParams) |
|  | returns the MMFF rest value for an angle |
| double | calcAngleForceConstant (const MMFFAngle \*mmffAngleParams) |
|  | returns the MMFF force constant for an angle |
| double | calcCosTheta (RDGeom::Point3D p1, RDGeom::Point3D p2, RDGeom::Point3D p3, double dist1, double dist2) |
|  | calculates and returns the cosine of the angle between points p1, p2, p3 |
| double | calcAngleBendEnergy (const double theta0, const double ka, bool isLinear, const double cosTheta) |
|  | calculates and returns the angle bending MMFF energy |
| void | calcAngleBendGrad (RDGeom::Point3D \*r, double \*dist, double \*\*g, double &dE\_dTheta, double &cosTheta, double &sinTheta) |
| double | calcBondRestLength (const MMFFBond \*mmffBondParams) |
|  | returns the MMFF rest length for a bond |
| double | calcBondForceConstant (const MMFFBond \*mmffBondParams) |
|  | returns the MMFF force constant for a bond |
| double | calcBondStretchEnergy (const double r0, const double kb, const double distance) |
|  | calculates and returns the bond stretching MMFF energy |
| double | calcUnscaledVdWMinimum (MMFFVdWCollection \*mmffVdW, const MMFFVdW \*mmffVdWParamsAtom1, const MMFFVdW \*mmffVdWParamsAtom2) |
|  | calculates and returns the unscaled minimum distance (R\*ij) for a MMFF VdW contact |
| double | calcUnscaledVdWWellDepth (double R\_star\_ij, const MMFFVdW \*mmffVdWParamsIAtom, const MMFFVdW \*mmffVdWParamsJAtom) |
|  | calculates and returns the unscaled well depth (epsilon) for a MMFF VdW contact |
| void | scaleVdWParams (double &R\_star\_ij, double &wellDepth, MMFFVdWCollection \*mmffVdW, const MMFFVdW \*mmffVdWParamsIAtom, const MMFFVdW \*mmffVdWParamsJAtom) |
|  | scales the VdW parameters |
| double | calcVdWEnergy (const double dist, const double R\_star\_ij, const double wellDepth) |
|  | calculates and returns the Van der Waals MMFF energy |
| double | calcEleEnergy (unsigned int idx1, unsigned int idx2, double dist, double chargeTerm, boost::uint8\_t dielModel, bool is1\_4) |
|  | calculates and returns the electrostatic MMFF energy |
| double | calcOopChi (const RDGeom::Point3D &iPoint, const RDGeom::Point3D &jPoint, const RDGeom::Point3D &kPoint, const RDGeom::Point3D &lPoint) |
|  | calculates and returns the Wilson angle (in degrees) |
| double | calcOopBendForceConstant (const MMFFOop \*mmffOopParams) |
|  | returns the out-of-plane force constant koop |
| double | calcOopBendEnergy (const double chi, const double koop) |
|  | calculates and returns the out-of-plane MMFF energy |
| std::pair< double, double > | calcStbnForceConstants (const std::pair< bool, const MMFFStbn \* > mmffStbnParams) |
|  | returns the std::pair of stretch-bend force constants for an angle |
| std::pair< double, double > | calcStretchBendEnergy (const double deltaDist1, const double deltaDist2, const double deltaTheta, const std::pair< double, double > forceConstants) |
|  | calculates and returns the stretch-bending MMFF energy |
| double | calcTorsionCosPhi (const RDGeom::Point3D &iPoint, const RDGeom::Point3D &jPoint, const RDGeom::Point3D &kPoint, const RDGeom::Point3D &lPoint) |
|  | calculates and returns the cosine of a torsion angle |
| boost::tuple< double, double,   double > | calcTorsionForceConstant (const MMFFTor \*mmffTorParams) |
|  | returns the 3-tuple of a torsion angle force constants |
| double | calcTorsionEnergy (const double V1, const double V2, const double V3, const double cosPhi) |
|  | calculates and returns the torsional MMFF energy |
| void | calcTorsionGrad (RDGeom::Point3D \*r, RDGeom::Point3D \*t, double \*d, double \*\*g, double &sinTerm, double &cosPhi) |

---

## Function Documentation

|  |  |  |  |
| --- | --- | --- | --- |
| double ForceFields::MMFF::Utils::calcAngleBendEnergy | ( | const double | *theta0*, |
|  |  | const double | *ka*, |
|  |  | bool | *isLinear*, |
|  |  | const double | *cosTheta* |  |
|  | ) |  |  |  |

calculates and returns the angle bending MMFF energy

|  |  |  |  |
| --- | --- | --- | --- |
| void ForceFields::MMFF::Utils::calcAngleBendGrad | ( | RDGeom::Point3D \* | *r*, |
|  |  | double \* | *dist*, |
|  |  | double \*\* | *g*, |
|  |  | double & | *dE\_dTheta*, |
|  |  | double & | *cosTheta*, |
|  |  | double & | *sinTheta* |  |
|  | ) |  |  |  |

|  |  |  |  |  |  |
| --- | --- | --- | --- | --- | --- |
| double ForceFields::MMFF::Utils::calcAngleForceConstant | ( | const MMFFAngle \* | *mmffAngleParams* | ) |  |

returns the MMFF force constant for an angle

|  |  |  |  |  |  |
| --- | --- | --- | --- | --- | --- |
| double ForceFields::MMFF::Utils::calcAngleRestValue | ( | const MMFFAngle \* | *mmffAngleParams* | ) |  |

returns the MMFF rest value for an angle

|  |  |  |  |  |  |
| --- | --- | --- | --- | --- | --- |
| double ForceFields::MMFF::Utils::calcBondForceConstant | ( | const MMFFBond \* | *mmffBondParams* | ) |  |

returns the MMFF force constant for a bond

|  |  |  |  |  |  |
| --- | --- | --- | --- | --- | --- |
| double ForceFields::MMFF::Utils::calcBondRestLength | ( | const MMFFBond \* | *mmffBondParams* | ) |  |

returns the MMFF rest length for a bond

|  |  |  |  |
| --- | --- | --- | --- |
| double ForceFields::MMFF::Utils::calcBondStretchEnergy | ( | const double | *r0*, |
|  |  | const double | *kb*, |
|  |  | const double | *distance* |  |
|  | ) |  |  |  |

calculates and returns the bond stretching MMFF energy

|  |  |  |  |
| --- | --- | --- | --- |
| double ForceFields::MMFF::Utils::calcCosTheta | ( | RDGeom::Point3D | *p1*, |
|  |  | RDGeom::Point3D | *p2*, |
|  |  | RDGeom::Point3D | *p3*, |
|  |  | double | *dist1*, |
|  |  | double | *dist2* |  |
|  | ) |  |  |  |

calculates and returns the cosine of the angle between points p1, p2, p3

|  |  |  |  |
| --- | --- | --- | --- |
| double ForceFields::MMFF::Utils::calcEleEnergy | ( | unsigned int | *idx1*, |
|  |  | unsigned int | *idx2*, |
|  |  | double | *dist*, |
|  |  | double | *chargeTerm*, |
|  |  | boost::uint8\_t | *dielModel*, |
|  |  | bool | *is1\_4* |  |
|  | ) |  |  |  |

calculates and returns the electrostatic MMFF energy

|  |  |  |  |
| --- | --- | --- | --- |
| double ForceFields::MMFF::Utils::calcOopBendEnergy | ( | const double | *chi*, |
|  |  | const double | *koop* |  |
|  | ) |  |  |  |

calculates and returns the out-of-plane MMFF energy

|  |  |  |  |  |  |
| --- | --- | --- | --- | --- | --- |
| double ForceFields::MMFF::Utils::calcOopBendForceConstant | ( | const MMFFOop \* | *mmffOopParams* | ) |  |

returns the out-of-plane force constant koop

|  |  |  |  |
| --- | --- | --- | --- |
| double ForceFields::MMFF::Utils::calcOopChi | ( | const RDGeom::Point3D & | *iPoint*, |
|  |  | const RDGeom::Point3D & | *jPoint*, |
|  |  | const RDGeom::Point3D & | *kPoint*, |
|  |  | const RDGeom::Point3D & | *lPoint* |  |
|  | ) |  |  |  |

calculates and returns the Wilson angle (in degrees)

|  |  |  |  |  |  |
| --- | --- | --- | --- | --- | --- |
| std::pair<double, double> ForceFields::MMFF::Utils::calcStbnForceConstants | ( | const std::pair< bool, const MMFFStbn \* > | *mmffStbnParams* | ) |  |

returns the std::pair of stretch-bend force constants for an angle

|  |  |  |  |
| --- | --- | --- | --- |
| std::pair<double, double> ForceFields::MMFF::Utils::calcStretchBendEnergy | ( | const double | *deltaDist1*, |
|  |  | const double | *deltaDist2*, |
|  |  | const double | *deltaTheta*, |
|  |  | const std::pair< double, double > | *forceConstants* |  |
|  | ) |  |  |  |

calculates and returns the stretch-bending MMFF energy

|  |  |  |  |
| --- | --- | --- | --- |
| double ForceFields::MMFF::Utils::calcTorsionCosPhi | ( | const RDGeom::Point3D & | *iPoint*, |
|  |  | const RDGeom::Point3D & | *jPoint*, |
|  |  | const RDGeom::Point3D & | *kPoint*, |
|  |  | const RDGeom::Point3D & | *lPoint* |  |
|  | ) |  |  |  |

calculates and returns the cosine of a torsion angle

|  |  |  |  |
| --- | --- | --- | --- |
| double ForceFields::MMFF::Utils::calcTorsionEnergy | ( | const double | *V1*, |
|  |  | const double | *V2*, |
|  |  | const double | *V3*, |
|  |  | const double | *cosPhi* |  |
|  | ) |  |  |  |

calculates and returns the torsional MMFF energy

|  |  |  |  |  |  |
| --- | --- | --- | --- | --- | --- |
| boost::tuple<double, double, double> ForceFields::MMFF::Utils::calcTorsionForceConstant | ( | const MMFFTor \* | *mmffTorParams* | ) |  |

returns the 3-tuple of a torsion angle force constants

|  |  |  |  |
| --- | --- | --- | --- |
| void ForceFields::MMFF::Utils::calcTorsionGrad | ( | RDGeom::Point3D \* | *r*, |
|  |  | RDGeom::Point3D \* | *t*, |
|  |  | double \* | *d*, |
|  |  | double \*\* | *g*, |
|  |  | double & | *sinTerm*, |
|  |  | double & | *cosPhi* |  |
|  | ) |  |  |  |

|  |  |  |  |
| --- | --- | --- | --- |
| double ForceFields::MMFF::Utils::calcUnscaledVdWMinimum | ( | MMFFVdWCollection \* | *mmffVdW*, |
|  |  | const MMFFVdW \* | *mmffVdWParamsAtom1*, |
|  |  | const MMFFVdW \* | *mmffVdWParamsAtom2* |  |
|  | ) |  |  |  |

calculates and returns the unscaled minimum distance (R\*ij) for a MMFF VdW contact

|  |  |  |  |
| --- | --- | --- | --- |
| double ForceFields::MMFF::Utils::calcUnscaledVdWWellDepth | ( | double | *R\_star\_ij*, |
|  |  | const MMFFVdW \* | *mmffVdWParamsIAtom*, |
|  |  | const MMFFVdW \* | *mmffVdWParamsJAtom* |  |
|  | ) |  |  |  |

calculates and returns the unscaled well depth (epsilon) for a MMFF VdW contact

|  |  |  |  |
| --- | --- | --- | --- |
| double ForceFields::MMFF::Utils::calcVdWEnergy | ( | const double | *dist*, |
|  |  | const double | *R\_star\_ij*, |
|  |  | const double | *wellDepth* |  |
|  | ) |  |  |  |

calculates and returns the Van der Waals MMFF energy

|  |  |  |  |
| --- | --- | --- | --- |
| void ForceFields::MMFF::Utils::scaleVdWParams | ( | double & | *R\_star\_ij*, |
|  |  | double & | *wellDepth*, |
|  |  | MMFFVdWCollection \* | *mmffVdW*, |
|  |  | const MMFFVdW \* | *mmffVdWParamsIAtom*, |
|  |  | const MMFFVdW \* | *mmffVdWParamsJAtom* |  |
|  | ) |  |  |  |

scales the VdW parameters

---

Generated on 16 Feb 2014 for RDKit-MMFF by 
 1.6.1 
