# Supplementary material for: Bringing the MMFF force field to the RDKit: implementation and validation
Source: J Cheminform. 2014 Jul 12;6:37. doi: 10.1186/s13321-014-0037-3 (PMC4116604; doi:10.1186/s13321-014-0037-3)
Supplement: Additional file 3: — Documentation. The file docs.zip expands to an HTML tree which documents the MMFF-related C++ and Python RDKit APIs; the documentation can be browsed opening the docs.html file in any HTML browser. The full RDKit documentation can be found at http://www.rdkit.org. [file s13321-014-0037-3-S3.zip › docs/cpp/namespacemembers_func.html]

RDKit-MMFF: Class Members


- Main Page
- Namespaces
- Classes
- Files
- Directories

- Namespace List
- Namespace Members

- All
- Functions
- Variables
- Typedefs
- Enumerator

- a
- b
- c
- g
- i
- s

### - a -

- addAngles()
  : RDKit::MMFF::Tools
- addBonds()
  : RDKit::MMFF::Tools
- addEle()
  : RDKit::MMFF::Tools
- addOop()
  : RDKit::MMFF::Tools
- addStretchBend()
  : RDKit::MMFF::Tools
- addTorsions()
  : RDKit::MMFF::Tools
- addVdW()
  : RDKit::MMFF::Tools
- areAtomsInSameAromaticRing()
  : RDKit::MMFF
- areAtomsInSameRingOfSize()
  : RDKit::MMFF

### - b -

- buildNeighborMatrix()
  : RDKit::MMFF::Tools

### - c -

- calcAngleBendEnergy()
  : ForceFields::MMFF::Utils
- calcAngleBendGrad()
  : ForceFields::MMFF::Utils
- calcAngleForceConstant()
  : ForceFields::MMFF::Utils
- calcAngleRestValue()
  : ForceFields::MMFF::Utils
- calcBondForceConstant()
  : ForceFields::MMFF::Utils
- calcBondRestLength()
  : ForceFields::MMFF::Utils
- calcBondStretchEnergy()
  : ForceFields::MMFF::Utils
- calcCosTheta()
  : ForceFields::MMFF::Utils
- calcEleEnergy()
  : ForceFields::MMFF::Utils
- calcOopBendEnergy()
  : ForceFields::MMFF::Utils
- calcOopBendForceConstant()
  : ForceFields::MMFF::Utils
- calcOopChi()
  : ForceFields::MMFF::Utils
- calcStbnForceConstants()
  : ForceFields::MMFF::Utils
- calcStretchBendEnergy()
  : ForceFields::MMFF::Utils
- calcTorsionCosPhi()
  : ForceFields::MMFF::Utils
- calcTorsionEnergy()
  : ForceFields::MMFF::Utils
- calcTorsionForceConstant()
  : ForceFields::MMFF::Utils
- calcTorsionGrad()
  : ForceFields::MMFF::Utils
- calcUnscaledVdWMinimum()
  : ForceFields::MMFF::Utils
- calcUnscaledVdWWellDepth()
  : ForceFields::MMFF::Utils
- calcVdWEnergy()
  : ForceFields::MMFF::Utils
- constructForceField()
  : RDKit::MMFF

### - g -

- getMMFFAngleBendEmpiricalRuleParams()
  : RDKit::MMFF
- getMMFFStretchBendType()
  : RDKit::MMFF
- getPeriodicTableRow()
  : RDKit::MMFF
- getTwoBitCell()
  : RDKit::MMFF::Tools

### - i -

- isAngleInRingOfSize3or4()
  : RDKit::MMFF
- isAtomInAromaticRingOfSize()
  : RDKit::MMFF
- isAtomNOxide()
  : RDKit::MMFF
- isDoubleZero()
  : ForceFields::MMFF
- isTorsionInRingOfSize4or5()
  : RDKit::MMFF

### - s -

- sanitizeMMFFMol()
  : RDKit::MMFF
- scaleVdWParams()
  : ForceFields::MMFF::Utils
- setMMFFAromaticity()
  : RDKit::MMFF
- setTwoBitCell()
  : RDKit::MMFF::Tools

---

Generated on 16 Feb 2014 for RDKit-MMFF by 
 1.6.1 
