# Supplementary material for: Bringing the MMFF force field to the RDKit: implementation and validation
Source: J Cheminform. 2014 Jul 12;6:37. doi: 10.1186/s13321-014-0037-3 (PMC4116604; doi:10.1186/s13321-014-0037-3)
Supplement: Additional file 3: — Documentation. The file docs.zip expands to an HTML tree which documents the MMFF-related C++ and Python RDKit APIs; the documentation can be browsed opening the docs.html file in any HTML browser. The full RDKit documentation can be found at http://www.rdkit.org. [file s13321-014-0037-3-S3.zip › docs/cpp/namespaceRDKit_1_1MMFF.html]

RDKit-MMFF: RDKit::MMFF Namespace Reference


- Main Page
- Namespaces
- Classes
- Files
- Directories

- Namespace List
- Namespace Members

RDKit::MMFF

# RDKit::MMFF Namespace Reference

|  |  |
| --- | --- |
| Namespaces | |
| namespace | Tools |
| Classes | |
| class | MMFFAtomProperties |
| class | MMFFMolProperties |
| Typedefs | |
| typedef boost::shared\_ptr  < MMFFAtomProperties > | MMFFAtomPropertiesPtr |
| Enumerations | |
| enum | { CONSTANT = 1, DISTANCE = 2 } |
| enum | { MMFF\_VERBOSITY\_NONE = 0, MMFF\_VERBOSITY\_LOW = 1, MMFF\_VERBOSITY\_HIGH = 2 } |
| Functions | |
| unsigned int | isAngleInRingOfSize3or4 (const ROMol &mol, const unsigned int idx1, const unsigned int idx2, const unsigned int idx3) |
| unsigned int | isTorsionInRingOfSize4or5 (const ROMol &mol, const unsigned int idx1, const unsigned int idx2, const unsigned int idx3, const unsigned int idx4) |
| bool | isAtomInAromaticRingOfSize (const Atom \*atom, const unsigned int ringSize) |
| bool | isAtomNOxide (const Atom \*atom) |
| bool | areAtomsInSameAromaticRing (const ROMol &mol, const unsigned int idx1, const unsigned int idx2) |
| bool | areAtomsInSameRingOfSize (const ROMol &mol, const unsigned int ringSize, const unsigned int numAtoms,...) |
| unsigned int | sanitizeMMFFMol (RWMol &mol) |
| void | setMMFFAromaticity (RWMol &mol) |
| const unsigned int | getMMFFStretchBendType (const unsigned int angleType, const unsigned int bondType1, const unsigned int bondType2) |
| const unsigned int | getPeriodicTableRow (const int atomicNum) |
| const   ForceFields::MMFF::MMFFAngle \* | getMMFFAngleBendEmpiricalRuleParams (const ROMol &mol, const ForceFields::MMFF::MMFFAngle \*oldMMFFAngleParams, const ForceFields::MMFF::MMFFProp \*mmffPropParamsCentralAtom, const ForceFields::MMFF::MMFFBond \*mmffBondParams1, const ForceFields::MMFF::MMFFBond \*mmffBondParams2, unsigned int idx1, unsigned int idx2, unsigned int idx3) |
| ForceFields::ForceField \* | constructForceField (ROMol &mol, double nonBondedThresh=100.0, int confId=-1, bool ignoreInterfragInteractions=true) |
|  | Builds and returns a MMFF force field for a molecule. |
| ForceFields::ForceField \* | constructForceField (ROMol &mol, MMFFMolProperties \*mmffMolProperties, double nonBondedThresh=100.0, int confId=-1, bool ignoreInterfragInteractions=true) |
|  | Builds and returns a MMFF force field for a molecule. |

---

## Typedef Documentation

|  |
| --- |
| typedef boost::shared\_ptr<MMFFAtomProperties> RDKit::MMFF::MMFFAtomPropertiesPtr |

Definition at line 41 of file AtomTyper.h.

---

## Enumeration Type Documentation

|  |
| --- |
| anonymous enum |

**Enumerator:**
:   |  |  |
    | --- | --- |
    | *CONSTANT* |  |
    | *DISTANCE* |  |

Definition at line 42 of file AtomTyper.h.

|  |
| --- |
| anonymous enum |

**Enumerator:**
:   |  |  |
    | --- | --- |
    | *MMFF\_VERBOSITY\_NONE* |  |
    | *MMFF\_VERBOSITY\_LOW* |  |
    | *MMFF\_VERBOSITY\_HIGH* |  |

Definition at line 46 of file AtomTyper.h.

---

## Function Documentation

|  |  |  |  |
| --- | --- | --- | --- |
| bool RDKit::MMFF::areAtomsInSameAromaticRing | ( | const ROMol & | *mol*, |
|  |  | const unsigned int | *idx1*, |
|  |  | const unsigned int | *idx2* |  |
|  | ) |  |  |  |

|  |  |  |  |
| --- | --- | --- | --- |
| bool RDKit::MMFF::areAtomsInSameRingOfSize | ( | const ROMol & | *mol*, |
|  |  | const unsigned int | *ringSize*, |
|  |  | const unsigned int | *numAtoms*, |
|  |  |  | *...* |  |
|  | ) |  |  |  |

|  |  |  |  |
| --- | --- | --- | --- |
| ForceFields::ForceField\* RDKit::MMFF::constructForceField | ( | ROMol & | *mol*, |
|  |  | MMFFMolProperties \* | *mmffMolProperties*, |
|  |  | double | *nonBondedThresh* = `100.0`, |
|  |  | int | *confId* = `-1`, |
|  |  | bool | *ignoreInterfragInteractions* = `true` |  |
|  | ) |  |  |  |

Builds and returns a MMFF force field for a molecule.

**Parameters:**
:   |  |  |  |
    | --- | --- | --- |
    |  | *mol* | the molecule to use |
    |  | *mmffMolProperties* | pointer to a MMFFMolProperties (as obtained by a call to setupMMFFForceField()) |
    |  | *nonBondedThresh* | the threshold to be used in adding non-bonded terms to the force field. Any non-bonded contact whose current distance is greater than `nonBondedThresh` \* the minimum value for that contact will not be included. |
    |  | *confId* | the optional conformer id, if this isn't provided, the molecule's default confId will be used. |
    |  | *ignoreInterfragInteractions* | if true, nonbonded terms will not be added between fragments |

**Returns:**
:   the new force field. The client is responsible for free'ing this.

|  |  |  |  |
| --- | --- | --- | --- |
| ForceFields::ForceField\* RDKit::MMFF::constructForceField | ( | ROMol & | *mol*, |
|  |  | double | *nonBondedThresh* = `100.0`, |
|  |  | int | *confId* = `-1`, |
|  |  | bool | *ignoreInterfragInteractions* = `true` |  |
|  | ) |  |  |  |

Builds and returns a MMFF force field for a molecule.

**Parameters:**
:   |  |  |  |
    | --- | --- | --- |
    |  | *mol* | the molecule to use |
    |  | *nonBondedThresh* | the threshold to be used in adding non-bonded terms to the force field. Any non-bonded contact whose current distance is greater than `nonBondedThresh` \* the minimum value for that contact will not be included. |
    |  | *confId* | the optional conformer id, if this isn't provided, the molecule's default confId will be used. |
    |  | *ignoreInterfragInteractions* | if true, nonbonded terms will not be added between fragments |

**Returns:**
:   the new force field. The client is responsible for free'ing this.

|  |  |  |  |
| --- | --- | --- | --- |
| const ForceFields::MMFF::MMFFAngle\* RDKit::MMFF::getMMFFAngleBendEmpiricalRuleParams | ( | const ROMol & | *mol*, |
|  |  | const ForceFields::MMFF::MMFFAngle \* | *oldMMFFAngleParams*, |
|  |  | const ForceFields::MMFF::MMFFProp \* | *mmffPropParamsCentralAtom*, |
|  |  | const ForceFields::MMFF::MMFFBond \* | *mmffBondParams1*, |
|  |  | const ForceFields::MMFF::MMFFBond \* | *mmffBondParams2*, |
|  |  | unsigned int | *idx1*, |
|  |  | unsigned int | *idx2*, |
|  |  | unsigned int | *idx3* |  |
|  | ) |  |  |  |

|  |  |  |  |
| --- | --- | --- | --- |
| const unsigned int RDKit::MMFF::getMMFFStretchBendType | ( | const unsigned int | *angleType*, |
|  |  | const unsigned int | *bondType1*, |
|  |  | const unsigned int | *bondType2* |  |
|  | ) |  |  |  |

|  |  |  |  |  |  |
| --- | --- | --- | --- | --- | --- |
| const unsigned int RDKit::MMFF::getPeriodicTableRow | ( | const int | *atomicNum* | ) |  |

|  |  |  |  |
| --- | --- | --- | --- |
| unsigned int RDKit::MMFF::isAngleInRingOfSize3or4 | ( | const ROMol & | *mol*, |
|  |  | const unsigned int | *idx1*, |
|  |  | const unsigned int | *idx2*, |
|  |  | const unsigned int | *idx3* |  |
|  | ) |  |  |  |

|  |  |  |  |
| --- | --- | --- | --- |
| bool RDKit::MMFF::isAtomInAromaticRingOfSize | ( | const Atom \* | *atom*, |
|  |  | const unsigned int | *ringSize* |  |
|  | ) |  |  |  |

|  |  |  |  |  |  |
| --- | --- | --- | --- | --- | --- |
| bool RDKit::MMFF::isAtomNOxide | ( | const Atom \* | *atom* | ) |  |

|  |  |  |  |
| --- | --- | --- | --- |
| unsigned int RDKit::MMFF::isTorsionInRingOfSize4or5 | ( | const ROMol & | *mol*, |
|  |  | const unsigned int | *idx1*, |
|  |  | const unsigned int | *idx2*, |
|  |  | const unsigned int | *idx3*, |
|  |  | const unsigned int | *idx4* |  |
|  | ) |  |  |  |

|  |  |  |  |  |  |
| --- | --- | --- | --- | --- | --- |
| unsigned int RDKit::MMFF::sanitizeMMFFMol | ( | RWMol & | *mol* | ) |  |

|  |  |  |  |  |  |
| --- | --- | --- | --- | --- | --- |
| void RDKit::MMFF::setMMFFAromaticity | ( | RWMol & | *mol* | ) |  |

---

Generated on 16 Feb 2014 for RDKit-MMFF by 
 1.6.1 
