# Supplementary material for: Bringing the MMFF force field to the RDKit: implementation and validation
Source: J Cheminform. 2014 Jul 12;6:37. doi: 10.1186/s13321-014-0037-3 (PMC4116604; doi:10.1186/s13321-014-0037-3)
Supplement: Additional file 3: — Documentation. The file docs.zip expands to an HTML tree which documents the MMFF-related C++ and Python RDKit APIs; the documentation can be browsed opening the docs.html file in any HTML browser. The full RDKit documentation can be found at http://www.rdkit.org. [file s13321-014-0037-3-S3.zip › docs/cpp/namespaceRDKit_1_1MMFF_1_1Tools.html]

RDKit-MMFF: RDKit::MMFF::Tools Namespace Reference


- Main Page
- Namespaces
- Classes
- Files
- Directories

- Namespace List
- Namespace Members

RDKit::MMFF::Tools

# RDKit::MMFF::Tools Namespace Reference

|  |  |
| --- | --- |
| Enumerations | |
| enum | { RELATION\_1\_2 = 0, RELATION\_1\_3 = 1, RELATION\_1\_4 = 2, RELATION\_1\_X = 3 } |
| Functions | |
| void | setTwoBitCell (boost::shared\_array< boost::uint8\_t > &res, unsigned int pos, boost::uint8\_t value) |
| boost::uint8\_t | getTwoBitCell (boost::shared\_array< boost::uint8\_t > &res, unsigned int pos) |
| boost::shared\_array  < boost::uint8\_t > | buildNeighborMatrix (const ROMol &mol) |
| void | addBonds (const ROMol &mol, MMFFMolProperties \*mmffMolProperties, ForceFields::ForceField \*field) |
| void | addAngles (const ROMol &mol, MMFFMolProperties \*mmffMolProperties, ForceFields::ForceField \*field) |
| void | addStretchBend (const ROMol &mol, MMFFMolProperties \*mmffMolProperties, ForceFields::ForceField \*field) |
| void | addOop (const ROMol &mol, MMFFMolProperties \*mmffMolProperties, ForceFields::ForceField \*field) |
| void | addTorsions (const ROMol &mol, MMFFMolProperties \*mmffMolProperties, ForceFields::ForceField \*field, std::string torsionBondSmarts="[!$(\*#\*)&!D1]~[!$(\*#\*)&!D1]") |
| void | addVdW (const ROMol &mol, int confId, MMFFMolProperties \*mmffMolProperties, ForceFields::ForceField \*field, boost::shared\_array< boost::uint8\_t > neighborMatrix, double nonBondedThresh=100.0, bool ignoreInterfragInteractions=true) |
| void | addEle (const ROMol &mol, int confId, MMFFMolProperties \*mmffMolProperties, ForceFields::ForceField \*field, boost::shared\_array< boost::uint8\_t > neighborMatrix, double nonBondedThresh=100.0, bool ignoreInterfragInteractions=true) |

---

## Enumeration Type Documentation

|  |
| --- |
| anonymous enum |

**Enumerator:**
:   |  |  |
    | --- | --- |
    | *RELATION\_1\_2* |  |
    | *RELATION\_1\_3* |  |
    | *RELATION\_1\_4* |  |
    | *RELATION\_1\_X* |  |

Definition at line 70 of file Builder.h.

---

## Function Documentation

|  |  |  |  |
| --- | --- | --- | --- |
| void RDKit::MMFF::Tools::addAngles | ( | const ROMol & | *mol*, |
|  |  | MMFFMolProperties \* | *mmffMolProperties*, |
|  |  | ForceFields::ForceField \* | *field* |  |
|  | ) |  |  |  |

|  |  |  |  |
| --- | --- | --- | --- |
| void RDKit::MMFF::Tools::addBonds | ( | const ROMol & | *mol*, |
|  |  | MMFFMolProperties \* | *mmffMolProperties*, |
|  |  | ForceFields::ForceField \* | *field* |  |
|  | ) |  |  |  |

|  |  |  |  |
| --- | --- | --- | --- |
| void RDKit::MMFF::Tools::addEle | ( | const ROMol & | *mol*, |
|  |  | int | *confId*, |
|  |  | MMFFMolProperties \* | *mmffMolProperties*, |
|  |  | ForceFields::ForceField \* | *field*, |
|  |  | boost::shared\_array< boost::uint8\_t > | *neighborMatrix*, |
|  |  | double | *nonBondedThresh* = `100.0`, |
|  |  | bool | *ignoreInterfragInteractions* = `true` |  |
|  | ) |  |  |  |

|  |  |  |  |
| --- | --- | --- | --- |
| void RDKit::MMFF::Tools::addOop | ( | const ROMol & | *mol*, |
|  |  | MMFFMolProperties \* | *mmffMolProperties*, |
|  |  | ForceFields::ForceField \* | *field* |  |
|  | ) |  |  |  |

|  |  |  |  |
| --- | --- | --- | --- |
| void RDKit::MMFF::Tools::addStretchBend | ( | const ROMol & | *mol*, |
|  |  | MMFFMolProperties \* | *mmffMolProperties*, |
|  |  | ForceFields::ForceField \* | *field* |  |
|  | ) |  |  |  |

|  |  |  |  |
| --- | --- | --- | --- |
| void RDKit::MMFF::Tools::addTorsions | ( | const ROMol & | *mol*, |
|  |  | MMFFMolProperties \* | *mmffMolProperties*, |
|  |  | ForceFields::ForceField \* | *field*, |
|  |  | std::string | *torsionBondSmarts* = `"[!$(*#*)&!D1]~[!$(*#*)&!D1]"` |  |
|  | ) |  |  |  |

|  |  |  |  |
| --- | --- | --- | --- |
| void RDKit::MMFF::Tools::addVdW | ( | const ROMol & | *mol*, |
|  |  | int | *confId*, |
|  |  | MMFFMolProperties \* | *mmffMolProperties*, |
|  |  | ForceFields::ForceField \* | *field*, |
|  |  | boost::shared\_array< boost::uint8\_t > | *neighborMatrix*, |
|  |  | double | *nonBondedThresh* = `100.0`, |
|  |  | bool | *ignoreInterfragInteractions* = `true` |  |
|  | ) |  |  |  |

|  |  |  |  |  |  |
| --- | --- | --- | --- | --- | --- |
| boost::shared\_array<boost::uint8\_t> RDKit::MMFF::Tools::buildNeighborMatrix | ( | const ROMol & | *mol* | ) |  |

|  |  |  |  |
| --- | --- | --- | --- |
| boost::uint8\_t RDKit::MMFF::Tools::getTwoBitCell | ( | boost::shared\_array< boost::uint8\_t > & | *res*, |
|  |  | unsigned int | *pos* |  |
|  | ) |  |  |  |

|  |  |  |  |
| --- | --- | --- | --- |
| void RDKit::MMFF::Tools::setTwoBitCell | ( | boost::shared\_array< boost::uint8\_t > & | *res*, |
|  |  | unsigned int | *pos*, |
|  |  | boost::uint8\_t | *value* |  |
|  | ) |  |  |  |

---

Generated on 16 Feb 2014 for RDKit-MMFF by 
 1.6.1 
