# Supplementary material for: Bringing the MMFF force field to the RDKit: implementation and validation
Source: J Cheminform. 2014 Jul 12;6:37. doi: 10.1186/s13321-014-0037-3 (PMC4116604; doi:10.1186/s13321-014-0037-3)
Supplement: Additional file 3: — Documentation. The file docs.zip expands to an HTML tree which documents the MMFF-related C++ and Python RDKit APIs; the documentation can be browsed opening the docs.html file in any HTML browser. The full RDKit documentation can be found at http://www.rdkit.org. [file s13321-014-0037-3-S3.zip › docs/cpp/Nonbonded_8h.html]

RDKit-MMFF: Nonbonded.h File Reference


- Main Page
- Namespaces
- Classes
- Files
- Directories

- File List
- File Members

ForceField » MMFF

# Nonbonded.h File Reference

`#include <ForceField/Contrib.h>`  
`#include <GraphMol/RDKitBase.h>`  
`#include <GraphMol/ForceFieldHelpers/MMFF/AtomTyper.h>`  

Go to the source code of this file.

|  |  |
| --- | --- |
| Classes | |
| class | ForceFields::MMFF::VdWContrib |
|  | the van der Waals term for MMFF More... |
| class | ForceFields::MMFF::EleContrib |
|  | the electrostatic term for MMFF More... |
| Namespaces | |
| namespace | ForceFields |
| namespace | ForceFields::MMFF |
| namespace | ForceFields::MMFF::Utils |
| Functions | |
| double | ForceFields::MMFF::Utils::calcUnscaledVdWMinimum (MMFFVdWCollection \*mmffVdW, const MMFFVdW \*mmffVdWParamsAtom1, const MMFFVdW \*mmffVdWParamsAtom2) |
|  | calculates and returns the unscaled minimum distance (R\*ij) for a MMFF VdW contact |
| double | ForceFields::MMFF::Utils::calcUnscaledVdWWellDepth (double R\_star\_ij, const MMFFVdW \*mmffVdWParamsIAtom, const MMFFVdW \*mmffVdWParamsJAtom) |
|  | calculates and returns the unscaled well depth (epsilon) for a MMFF VdW contact |
| void | ForceFields::MMFF::Utils::scaleVdWParams (double &R\_star\_ij, double &wellDepth, MMFFVdWCollection \*mmffVdW, const MMFFVdW \*mmffVdWParamsIAtom, const MMFFVdW \*mmffVdWParamsJAtom) |
|  | scales the VdW parameters |
| double | ForceFields::MMFF::Utils::calcVdWEnergy (const double dist, const double R\_star\_ij, const double wellDepth) |
|  | calculates and returns the Van der Waals MMFF energy |
| double | ForceFields::MMFF::Utils::calcEleEnergy (unsigned int idx1, unsigned int idx2, double dist, double chargeTerm, boost::uint8\_t dielModel, bool is1\_4) |
|  | calculates and returns the electrostatic MMFF energy |

---

Generated on 16 Feb 2014 for RDKit-MMFF by 
 1.6.1 
