# Supplementary material for: Bringing the MMFF force field to the RDKit: implementation and validation
Source: J Cheminform. 2014 Jul 12;6:37. doi: 10.1186/s13321-014-0037-3 (PMC4116604; doi:10.1186/s13321-014-0037-3)
Supplement: Additional file 3: — Documentation. The file docs.zip expands to an HTML tree which documents the MMFF-related C++ and Python RDKit APIs; the documentation can be browsed opening the docs.html file in any HTML browser. The full RDKit documentation can be found at http://www.rdkit.org. [file s13321-014-0037-3-S3.zip › docs/cpp/Nonbonded_8h_source.html]

RDKit-MMFF: Nonbonded.h Source File


- Main Page
- Namespaces
- Classes
- Files
- Directories

- File List
- File Members

ForceField » MMFF

# Nonbonded.h

Go to the documentation of this file.

```
00001 //
00002 //  Copyright (C) 2013 Paolo Tosco
00003 //
00004 //  Copyright (C) 2004-2006 Rational Discovery LLC
00005 //
00006 //   @@ All Rights Reserved @@
00007 //  This file is part of the RDKit.
00008 //  The contents are covered by the terms of the BSD license
00009 //  which is included in the file license.txt, found at the root
00010 //  of the RDKit source tree.
00011 //
00012 #ifndef __RD_MMFFNONBONDED_H__
00013 #define __RD_MMFFNONBONDED_H__
00014 #include <ForceField/Contrib.h>
00015 #include <GraphMol/RDKitBase.h>
00016 #include <GraphMol/ForceFieldHelpers/MMFF/AtomTyper.h>
00017 
00018 namespace ForceFields {
00019   namespace MMFF {
00020     class MMFFVdWCollection;
00021     class MMFFVdW;
00022     //! the van der Waals term for MMFF
00023     class VdWContrib : public ForceFieldContrib {
00024     public:
00025       VdWContrib() : d_at1Idx(-1), d_at2Idx(-1) {};
00026 
00027       //! Constructor
00028       /*!
00029         \param owner       pointer to the owning ForceField
00030         \param idx1        index of end1 in the ForceField's positions
00031         \param idx2        index of end2 in the ForceField's positions
00032 
00033       */
00034       VdWContrib(ForceField *owner, unsigned int idx1, unsigned int idx2,
00035         MMFFVdWCollection *mmffVdW, const MMFFVdW *mmffVdWParamsAtom1,
00036         const MMFFVdW *mmffVdWParamsAtom2);
00037       double getEnergy(double *pos) const;
00038       void getGrad(double *pos, double *grad) const;
00039     
00040     private:
00041       int d_at1Idx, d_at2Idx;
00042       double d_R_star_ij;       //!< the preferred length of the contact
00043       double d_wellDepth; //!< the vdW well depth (strength of the interaction)
00044 
00045     };
00046 
00047     //! the electrostatic term for MMFF
00048     class EleContrib : public ForceFieldContrib {
00049     public:
00050       EleContrib() : d_at1Idx(-1), d_at2Idx(-1) {};
00051 
00052       //! Constructor
00053       /*!
00054         \param owner       pointer to the owning ForceField
00055         \param idx1        index of end1 in the ForceField's positions
00056         \param idx2        index of end2 in the ForceField's positions
00057 
00058       */
00059       EleContrib(ForceField *owner, unsigned int idx1, unsigned int idx2,
00060         double chargeTerm, boost::uint8_t dielModel, bool is1_4);
00061       double getEnergy(double *pos) const;
00062       void getGrad(double *pos, double *grad) const;
00063     
00064     private:
00065       int d_at1Idx, d_at2Idx;
00066       double d_chargeTerm;    //!< q1 * q2 / D
00067       boost::uint8_t d_dielModel;    //!< dielectric model (1: constant; 2: distance-dependent)
00068       bool d_is1_4;    //!< flag set for atoms in a 1,4 relationship
00069 
00070     };
00071 
00072     namespace Utils {
00073       //! calculates and returns the unscaled minimum distance (R*ij) for a MMFF VdW contact
00074       double calcUnscaledVdWMinimum(MMFFVdWCollection *mmffVdW,
00075         const MMFFVdW *mmffVdWParamsAtom1, const MMFFVdW *mmffVdWParamsAtom2);
00076       //! calculates and returns the unscaled well depth (epsilon) for a MMFF VdW contact
00077       double calcUnscaledVdWWellDepth(double R_star_ij,
00078         const MMFFVdW *mmffVdWParamsIAtom, const MMFFVdW *mmffVdWParamsJAtom);
00079       //! scales the VdW parameters
00080       void scaleVdWParams(double &R_star_ij, double &wellDepth,
00081         MMFFVdWCollection *mmffVdW, const MMFFVdW *mmffVdWParamsIAtom,
00082         const MMFFVdW *mmffVdWParamsJAtom);
00083       //! calculates and returns the Van der Waals MMFF energy
00084       double calcVdWEnergy(const double dist,
00085         const double R_star_ij, const double wellDepth);
00086       //! calculates and returns the electrostatic MMFF energy
00087       double calcEleEnergy(unsigned int idx1, unsigned int idx2, double dist,
00088         double chargeTerm, boost::uint8_t dielModel, bool is1_4);
00089     }
00090   }
00091 }
00092 #endif
```

---

Generated on 16 Feb 2014 for RDKit-MMFF by 
 1.6.1 
