# Supplementary material for: Bringing the MMFF force field to the RDKit: implementation and validation
Source: J Cheminform. 2014 Jul 12;6:37. doi: 10.1186/s13321-014-0037-3 (PMC4116604; doi:10.1186/s13321-014-0037-3)
Supplement: Additional file 3: — Documentation. The file docs.zip expands to an HTML tree which documents the MMFF-related C++ and Python RDKit APIs; the documentation can be browsed opening the docs.html file in any HTML browser. The full RDKit documentation can be found at http://www.rdkit.org. [file s13321-014-0037-3-S3.zip › docs/cpp/OopBend_8h_source.html]

RDKit-MMFF: OopBend.h Source File


- Main Page
- Namespaces
- Classes
- Files
- Directories

- File List
- File Members

ForceField » MMFF

# OopBend.h

Go to the documentation of this file.

```
00001 //
00002 //  Copyright (C) 2013 Paolo Tosco
00003 //
00004 //  Copyright (C) 2004-2006 Rational Discovery LLC
00005 //
00006 //   @@ All Rights Reserved @@
00007 //  This file is part of the RDKit.
00008 //  The contents are covered by the terms of the BSD license
00009 //  which is included in the file license.txt, found at the root
00010 //  of the RDKit source tree.
00011 //
00012 #ifndef __RD_MMFFOopBend_H__
00013 #define __RD_MMFFOopBend_H__
00014 
00015 #include <ForceField/Contrib.h>
00016 #include <Geometry/point.h>
00017 
00018 
00019 namespace ForceFields {
00020   namespace MMFF {
00021     class MMFFOop;
00022 
00023     //! the out-of-plane term for MMFF
00024     class OopBendContrib : public ForceFieldContrib {
00025     public:
00026       OopBendContrib() : d_at1Idx(-1), d_at2Idx(-1), d_at3Idx(-1), d_at4Idx(-1) {};
00027       //! Constructor
00028       /*!
00029         The Wilson angle is between the vector formed by atom2-atom4
00030   and the angle formed by atom1-atom2-atom3
00031         
00032         \param owner       pointer to the owning ForceField
00033         \param idx1        index of atom1 in the ForceField's positions
00034         \param idx2        index of atom2 in the ForceField's positions
00035         \param idx3        index of atom3 in the ForceField's positions
00036         \param idx4        index of atom4 in the ForceField's positions
00037       */
00038       OopBendContrib(ForceField *owner, unsigned int idx1, unsigned int idx2,
00039         unsigned int idx3, unsigned int idx4, const MMFFOop *mmffOopParams);
00040       double getEnergy(double *pos) const;
00041       void getGrad(double *pos, double *grad) const;
00042     private:
00043       int d_at1Idx, d_at2Idx, d_at3Idx, d_at4Idx;
00044       double d_koop;
00045     };
00046 
00047     namespace Utils {
00048       //! calculates and returns the Wilson angle (in degrees)
00049       double calcOopChi(const RDGeom::Point3D &iPoint, const RDGeom::Point3D &jPoint,
00050         const RDGeom::Point3D &kPoint, const RDGeom::Point3D &lPoint);
00051       //! returns the out-of-plane force constant koop
00052       double calcOopBendForceConstant(const MMFFOop *mmffOopParams);
00053       //! calculates and returns the out-of-plane MMFF energy
00054       double calcOopBendEnergy(const double chi, const double koop);
00055     }
00056   }
00057 }
00058 #endif
```

---

Generated on 16 Feb 2014 for RDKit-MMFF by 
 1.6.1 
