# Supplementary material for: Bringing the MMFF force field to the RDKit: implementation and validation
Source: J Cheminform. 2014 Jul 12;6:37. doi: 10.1186/s13321-014-0037-3 (PMC4116604; doi:10.1186/s13321-014-0037-3)
Supplement: Additional file 3: — Documentation. The file docs.zip expands to an HTML tree which documents the MMFF-related C++ and Python RDKit APIs; the documentation can be browsed opening the docs.html file in any HTML browser. The full RDKit documentation can be found at http://www.rdkit.org. [file s13321-014-0037-3-S3.zip › docs/cpp/Params_8h.html]

RDKit-MMFF: Params.h File Reference


- Main Page
- Namespaces
- Classes
- Files
- Directories

- File List
- File Members

ForceField » MMFF

# Params.h File Reference

`#include <RDGeneral/Invariant.h>`  
`#include <cmath>`  
`#include <string>`  
`#include <vector>`  
`#include <algorithm>`  
`#include <map>`  
`#include <iostream>`  
`#include <boost/cstdint.hpp>`  

Go to the source code of this file.

|  |  |
| --- | --- |
| Classes | |
| class | ForceFields::MMFF::MMFFDef |
|  | class to store MMFF atom type equivalence levels More... |
| class | ForceFields::MMFF::MMFFProp |
|  | class to store MMFF Properties More... |
| class | ForceFields::MMFF::MMFFPBCI |
|  | class to store MMFF Partial Bond Charge Increments More... |
| class | ForceFields::MMFF::MMFFChg |
| class | ForceFields::MMFF::MMFFBond |
|  | class to store MMFF parameters for bond stretching More... |
| class | ForceFields::MMFF::MMFFCovRadPauEle |
| class | ForceFields::MMFF::MMFFAngle |
|  | class to store MMFF parameters for angle bending More... |
| class | ForceFields::MMFF::MMFFStbn |
|  | class to store MMFF parameters for stretch-bending More... |
| class | ForceFields::MMFF::MMFFOop |
|  | class to store MMFF parameters for out-of-plane bending More... |
| class | ForceFields::MMFF::MMFFTor |
|  | class to store MMFF parameters for torsions More... |
| class | ForceFields::MMFF::MMFFVdW |
|  | class to store MMFF parameters for non-bonded Van der Waals More... |
| class | ForceFields::MMFF::MMFFAromCollection |
| class | ForceFields::MMFF::MMFFDefCollection |
| class | ForceFields::MMFF::MMFFPropCollection |
| class | ForceFields::MMFF::MMFFPBCICollection |
| class | ForceFields::MMFF::MMFFChgCollection |
| class | ForceFields::MMFF::MMFFBondCollection |
| class | ForceFields::MMFF::MMFFBndkCollection |
| class | ForceFields::MMFF::MMFFCovRadPauEleCollection |
| class | ForceFields::MMFF::MMFFAngleCollection |
| class | ForceFields::MMFF::MMFFStbnCollection |
| class | ForceFields::MMFF::MMFFDfsbCollection |
| class | ForceFields::MMFF::MMFFOopCollection |
| class | ForceFields::MMFF::MMFFTorCollection |
| class | ForceFields::MMFF::MMFFVdWCollection |
| Namespaces | |
| namespace | ForceFields |
| namespace | ForceFields::MMFF |
| Defines | |
| #define | M\_PI   3.14159265358979323846 |
| Functions | |
| const bool | ForceFields::MMFF::isDoubleZero (const double x) |
| Variables | |
| const double | ForceFields::MMFF::DEG2RAD = M\_PI / 180.0 |
| const double | ForceFields::MMFF::RAD2DEG = 180.0 / M\_PI |

---

## Define Documentation

|  |
| --- |
| #define M\_PI   3.14159265358979323846 |

Definition at line 25 of file Params.h.

---

Generated on 16 Feb 2014 for RDKit-MMFF by 
 1.6.1 
