# Supplementary material for: Bringing the MMFF force field to the RDKit: implementation and validation
Source: J Cheminform. 2014 Jul 12;6:37. doi: 10.1186/s13321-014-0037-3 (PMC4116604; doi:10.1186/s13321-014-0037-3)
Supplement: Additional file 3: — Documentation. The file docs.zip expands to an HTML tree which documents the MMFF-related C++ and Python RDKit APIs; the documentation can be browsed opening the docs.html file in any HTML browser. The full RDKit documentation can be found at http://www.rdkit.org. [file s13321-014-0037-3-S3.zip › docs/cpp/Params_8h_source.html]

RDKit-MMFF: Params.h Source File


- Main Page
- Namespaces
- Classes
- Files
- Directories

- File List
- File Members

ForceField » MMFF

# Params.h

Go to the documentation of this file.

```
00001 //
00002 //  Copyright (C) 2013 Paolo Tosco
00003 //
00004 //  Copyright (C) 2004-2006 Rational Discovery LLC
00005 //
00006 //   @@ All Rights Reserved @@
00007 //  This file is part of the RDKit.
00008 //  The contents are covered by the terms of the BSD license
00009 //  which is included in the file license.txt, found at the root
00010 //  of the RDKit source tree.
00011 //
00012 #ifndef __RD_MMFFPARAMS_H__
00013 #define __RD_MMFFPARAMS_H__
00014 
00015 #include <RDGeneral/Invariant.h>
00016 #include <cmath>
00017 #include <string>
00018 #include <vector>
00019 #include <algorithm>
00020 #include <map>
00021 #include <iostream>
00022 #include <boost/cstdint.hpp>
00023 
00024 #ifndef M_PI
00025 #define M_PI           3.14159265358979323846
00026 #endif
00027 
00028 // binary searches are slightly faster than std::map;
00029 // however when I moved to binary searches I had already
00030 // written the code for std::map, so the two methods
00031 // can be toggled defining RDKIT_MMFF_PARAMS_USE_STD_MAP
00032 
00033 //#define RDKIT_MMFF_PARAMS_USE_STD_MAP 1
00034 
00035 namespace ForceFields {
00036   namespace MMFF {
00037 
00038     const double DEG2RAD = M_PI / 180.0;
00039     const double RAD2DEG = 180.0 / M_PI;
00040     inline const bool isDoubleZero(const double x) {
00041       return ((x < 1.0e-10) && (x > -1.0e-10));
00042     }
00043 
00044     //! class to store MMFF atom type equivalence levels
00045     class MMFFDef {
00046     public:
00047       boost::uint8_t eqLevel[4];
00048     };
00049 
00050     //! class to store MMFF Properties
00051     class MMFFProp {
00052     public:
00053       boost::uint8_t atno;
00054       boost::uint8_t crd;
00055       boost::uint8_t val;
00056       boost::uint8_t pilp;
00057       boost::uint8_t mltb;
00058       boost::uint8_t arom;
00059       boost::uint8_t linh;
00060       boost::uint8_t sbmb;
00061     };
00062 
00063     //! class to store MMFF Partial Bond Charge Increments
00064     class MMFFPBCI {
00065     public:
00066       double pbci;
00067       double fcadj;
00068     };
00069 
00070     //! class to store MMFF bond-charge-increment parameters used to
00071     //! construct MMFF partial atomic charges
00072     class MMFFChg {
00073     public:
00074       double bci;
00075     };
00076 
00077     //! class to store MMFF parameters for bond stretching
00078     class MMFFBond {
00079     public:
00080       double kb;
00081       double r0;
00082     };
00083 
00084     //! class to store covalent radius and Pauling electronegativity
00085     //! values for MMFF bond stretching empirical rule
00086     class MMFFCovRadPauEle {
00087     public:
00088       double r0;
00089       double chi;
00090     };
00091 
00092     //! class to store MMFF parameters for angle bending
00093     class MMFFAngle {
00094     public:
00095       double ka;
00096       double theta0;
00097     };
00098 
00099     //! class to store MMFF parameters for stretch-bending
00100     class MMFFStbn {
00101     public:
00102       double kbaIJK;
00103       double kbaKJI;
00104     };
00105 
00106     //! class to store MMFF parameters for out-of-plane bending
00107     class MMFFOop {
00108     public:
00109       double koop;
00110     };
00111 
00112     //! class to store MMFF parameters for torsions
00113     class MMFFTor {
00114     public:
00115       double V1;
00116       double V2;
00117       double V3;
00118     };
00119 
00120     //! class to store MMFF parameters for non-bonded Van der Waals
00121     class MMFFVdW {
00122     public:
00123       double alpha_i;
00124       double N_i;
00125       double A_i;
00126       double G_i;
00127       double R_star;
00128       boost::uint8_t DA;
00129     };
00130 
00131     class MMFFAromCollection {
00132     public:
00133       //! gets a pointer to the singleton MMFFAromCollection
00134       /*!
00135         \param mmffArom (optional) a string with parameter data. See
00136          below for more information about this argument
00137 
00138         \return a pointer to the singleton MMFFAromCollection
00139 
00140         <b>Notes:</b>
00141           - do <b>not</b> delete the pointer returned here
00142           - if the singleton MMFFAromCollection has already been instantiated and
00143             \c mmffArom is empty, the singleton will be returned.
00144           - if \c mmffArom is empty and the singleton MMFFAromCollection has
00145             not yet been instantiated, the default MMFFArom parameters (from Params.cpp)
00146             will be used.
00147           - if \c mmffArom is supplied, a new singleton will be instantiated.
00148             The current instantiation (if there is one) will be deleted.
00149       */
00150       static MMFFAromCollection *getMMFFArom(const boost::uint8_t *aromatic_types = NULL);
00151       //! Looks up the parameters for a particular key and returns them.
00152       /*!
00153         \return a pointer to the MMFFArom object, NULL on failure.
00154       */
00155       const bool isMMFFAromatic(const unsigned int atomType) const {
00156         return ((std::find(d_params.begin(), d_params.end(),
00157           atomType) != d_params.end()) ? true : false);
00158       }
00159     private:
00160       //! to force this to be a singleton, the constructor must be private
00161       MMFFAromCollection(const boost::uint8_t mmffArom[]);
00162       static class MMFFAromCollection *ds_instance;    //!< the singleton
00163       std::vector<boost::uint8_t> d_params;  //!< the aromatic type vector
00164     };
00165 
00166     class MMFFDefCollection {
00167     public:
00168       //! gets a pointer to the singleton MMFFDefCollection
00169       /*!
00170         \param mmffDef (optional) a string with parameter data. See
00171          below for more information about this argument
00172 
00173         \return a pointer to the singleton MMFFDefCollection
00174 
00175         <b>Notes:</b>
00176           - do <b>not</b> delete the pointer returned here
00177           - if the singleton MMFFDefCollection has already been instantiated and
00178             \c mmffDef is empty, the singleton will be returned.
00179           - if \c mmffDef is empty and the singleton MMFFDefCollection has
00180             not yet been instantiated, the default MMFFDef parameters (from Params.cpp)
00181             will be used.
00182           - if \c mmffDef is supplied, a new singleton will be instantiated.
00183             The current instantiation (if there is one) will be deleted.
00184       */
00185       static MMFFDefCollection *getMMFFDef(const std::string &mmffDef="");
00186       //! Looks up the parameters for a particular key and returns them.
00187       /*!
00188         \return a pointer to the MMFFDef object, NULL on failure.
00189       */
00190       const MMFFDef *operator()(const unsigned int atomType) const {
00191         #ifdef RDKIT_MMFF_PARAMS_USE_STD_MAP
00192         std::map<const unsigned int, MMFFDef>::const_iterator res;
00193         res = d_params.find(atomType);
00194 
00195         return ((res != d_params.end()) ? &((*res).second) : NULL);
00196         #else
00197         return ((atomType && (atomType <= d_params.size()))
00198           ? &d_params[atomType - 1] : NULL);
00199         #endif
00200       }
00201     private:
00202       //! to force this to be a singleton, the constructor must be private
00203       MMFFDefCollection(std::string mmffDef);
00204       static class MMFFDefCollection *ds_instance;    //!< the singleton
00205       #ifdef RDKIT_MMFF_PARAMS_USE_STD_MAP
00206       std::map<const unsigned int, MMFFDef> d_params;  //!< the parameter map
00207       #else
00208       std::vector<MMFFDef> d_params;  //!< the parameter vector
00209       #endif
00210     };
00211 
00212     class MMFFPropCollection {
00213     public:
00214       //! gets a pointer to the singleton MMFFPropCollection
00215       /*!
00216         \param mmffProp (optional) a string with parameter data. See
00217          below for more information about this argument
00218 
00219         \return a pointer to the singleton MMFFPropCollection
00220 
00221         <b>Notes:</b>
00222           - do <b>not</b> delete the pointer returned here
00223           - if the singleton MMFFPropCollection has already been instantiated and
00224             \c mmffProp is empty, the singleton will be returned.
00225           - if \c mmffProp is empty and the singleton MMFFPropCollection has
00226             not yet been instantiated, the default parameters (from Params.cpp)
00227             will be used.
00228           - if \c mmffProp is supplied, a new singleton will be instantiated.
00229             The current instantiation (if there is one) will be deleted.
00230       */
00231       static MMFFPropCollection *getMMFFProp(const std::string &mmffProp="");
00232       //! Looks up the parameters for a particular key and returns them.
00233       /*!
00234         \return a pointer to the MMFFProp object, NULL on failure.
00235       */
00236       const MMFFProp *operator()(const unsigned int atomType) const {
00237         #ifdef RDKIT_MMFF_PARAMS_USE_STD_MAP
00238         std::map<const unsigned int, MMFFProp>::const_iterator res;
00239         res = d_params.find(atomType);
00240 
00241         return ((res != d_params.end()) ? &((*res).second) : NULL);
00242         #else
00243         std::pair<std::vector<boost::uint8_t>::const_iterator,
00244           std::vector<boost::uint8_t>::const_iterator> bounds =
00245           std::equal_range(d_iAtomType.begin(), d_iAtomType.end(), atomType);
00246 
00247         return ((bounds.first != bounds.second)
00248           ? &d_params[bounds.first - d_iAtomType.begin()] : NULL);
00249         #endif
00250       }
00251     private:
00252       //! to force this to be a singleton, the constructor must be private
00253       MMFFPropCollection(std::string mmffProp);
00254       static class MMFFPropCollection *ds_instance;    //!< the singleton
00255       #ifdef RDKIT_MMFF_PARAMS_USE_STD_MAP
00256       std::map<const unsigned int, MMFFProp> d_params;  //!< the parameter map
00257       #else
00258       std::vector<MMFFProp> d_params;
00259       std::vector<boost::uint8_t> d_iAtomType;  //!< the parameter vector
00260       #endif
00261     };
00262 
00263     class MMFFPBCICollection {
00264     public:
00265       //! gets a pointer to the singleton MMFFPBCICollection
00266       /*!
00267         \param mmffPBCI (optional) a string with parameter data. See
00268          below for more information about this argument
00269 
00270         \return a pointer to the singleton MMFFPBCICollection
00271 
00272         <b>Notes:</b>
00273           - do <b>not</b> delete the pointer returned here
00274           - if the singleton MMFFPBCICollection has already been instantiated and
00275             \c mmffPBCI is empty, the singleton will be returned.
00276           - if \c mmffPBCI is empty and the singleton MMFFPBCICollection has
00277             not yet been instantiated, the default parameters (from Params.cpp)
00278             will be used.
00279           - if \c mmffPBCI is supplied, a new singleton will be instantiated.
00280             The current instantiation (if there is one) will be deleted.
00281       */
00282       static MMFFPBCICollection *getMMFFPBCI(const std::string &mmffPBCI = "");
00283       //! Looks up the parameters for a particular key and returns them.
00284       /*!
00285         \return a pointer to the MMFFPBCI object, NULL on failure.
00286       */
00287       const MMFFPBCI *operator()(const unsigned int atomType) const {
00288         #ifdef RDKIT_MMFF_PARAMS_USE_STD_MAP
00289         std::map<const unsigned int, MMFFPBCI>::const_iterator res;
00290         res = d_params.find(atomType);
00291 
00292         return ((res != d_params.end()) ? &((*res).second) : NULL);
00293         #else
00294         return ((atomType && (atomType <= d_params.size()))
00295           ? &d_params[atomType - 1] : NULL);
00296         #endif
00297       }
00298     private:
00299       //! to force this to be a singleton, the constructor must be private
00300       MMFFPBCICollection(std::string mmffPBCI);
00301       static class MMFFPBCICollection *ds_instance;    //!< the singleton
00302       #ifdef RDKIT_MMFF_PARAMS_USE_STD_MAP
00303       std::map<const unsigned int, MMFFPBCI> d_params;  //!< the parameter map
00304       #else
00305       std::vector<MMFFPBCI> d_params;  //!< the parameter vector
00306       #endif
00307     };
00308 
00309     class MMFFChgCollection {
00310     public:
00311       //! gets a pointer to the singleton MMFFChgCollection
00312       /*!
00313         \param mmffChg (optional) a string with parameter data. See
00314          below for more information about this argument
00315 
00316         \return a pointer to the singleton MMFFChgCollection
00317 
00318         <b>Notes:</b>
00319           - do <b>not</b> delete the pointer returned here
00320           - if the singleton MMFFChgCollection has already been instantiated and
00321             \c mmffChg is empty, the singleton will be returned.
00322           - if \c mmffChg is empty and the singleton MMFFChgCollection has
00323             not yet been instantiated, the default parameters (from Params.cpp)
00324             will be used.
00325           - if \c mmffChg is supplied, a new singleton will be instantiated.
00326             The current instantiation (if there is one) will be deleted.
00327       */
00328       static MMFFChgCollection *getMMFFChg(const std::string &mmffChg = "");
00329       //! Looks up the parameters for a particular key and returns them.
00330       /*!
00331         \return a pointer to the MMFFChg object, NULL on failure.
00332       */
00333       const std::pair<int, const MMFFChg *> getMMFFChgParams(const unsigned int bondType,
00334         const unsigned int iAtomType, const unsigned int jAtomType) {
00335 
00336         int sign = -1;
00337         const MMFFChg *mmffChgParams = NULL;
00338         unsigned int canIAtomType = iAtomType;
00339         unsigned int canJAtomType = jAtomType;
00340         if (iAtomType > jAtomType) {
00341           canIAtomType = jAtomType;
00342           canJAtomType = iAtomType;
00343           sign = 1;
00344         }
00345         #ifdef RDKIT_MMFF_PARAMS_USE_STD_MAP
00346         std::map<const unsigned int, std::map<const unsigned int, MMFFChg> >::const_iterator res1;
00347         std::map<const unsigned int, MMFFChg>::const_iterator res2;
00348         res1 = d_params[bondType].find(canIAtomType);
00349         if (res1 != d_params[bondType].end()) {
00350           res2 = ((*res1).second).find(canJAtomType);
00351           if (res2 != ((*res1).second).end()) {
00352             mmffChgParams = &((*res2).second);
00353           }
00354         }
00355         #else
00356         std::pair<std::vector<boost::uint8_t>::const_iterator,
00357           std::vector<boost::uint8_t>::const_iterator> bounds;
00358         
00359         bounds = std::equal_range(d_iAtomType.begin(), d_iAtomType.end(), canIAtomType);
00360         if (bounds.first != bounds.second) {
00361           bounds = std::equal_range(d_jAtomType.begin() + (bounds.first - d_iAtomType.begin()),
00362             d_jAtomType.begin() + (bounds.second - d_iAtomType.begin()), canJAtomType);
00363           if (bounds.first != bounds.second) {
00364             bounds = std::equal_range
00365               (d_bondType.begin() + (bounds.first - d_jAtomType.begin()),
00366               d_bondType.begin() + (bounds.second - d_jAtomType.begin()), bondType);
00367             if (bounds.first != bounds.second) {
00368               mmffChgParams = &d_params[bounds.first - d_bondType.begin()];
00369             }
00370           }
00371         }
00372         #endif
00373 
00374         return std::make_pair(sign, mmffChgParams);
00375       }
00376     private:
00377       //! to force this to be a singleton, the constructor must be private
00378       MMFFChgCollection(std::string mmffChg);
00379       static class MMFFChgCollection *ds_instance;    //!< the singleton
00380       //!< the parameter 3D-map
00381       #ifdef RDKIT_MMFF_PARAMS_USE_STD_MAP
00382       std::map<const unsigned int, std::map<const unsigned int,
00383         std::map<const unsigned int, MMFFChg> > > d_params;  //!< the parameter 3D-map
00384       #else
00385       std::vector<MMFFChg> d_params;  //! the parameter vector
00386       std::vector<boost::uint8_t> d_iAtomType;  //! atom type vector for atom i
00387       std::vector<boost::uint8_t> d_jAtomType;  //! atom type vector for atom j
00388       std::vector<boost::uint8_t> d_bondType;  //! bond type vector for bond i-j
00389       #endif
00390     };
00391 
00392     class MMFFBondCollection {
00393     public:
00394       //! gets a pointer to the singleton MMFFBondCollection
00395       /*!
00396         \param mmffBond (optional) a string with parameter data. See
00397          below for more information about this argument
00398 
00399         \return a pointer to the singleton MMFFBondCollection
00400 
00401         <b>Notes:</b>
00402           - do <b>not</b> delete the pointer returned here
00403           - if the singleton MMFFBondCollection has already been instantiated and
00404             \c mmffBond is empty, the singleton will be returned.
00405           - if \c mmffBond is empty and the singleton MMFFBondCollection has
00406             not yet been instantiated, the default parameters (from Params.cpp)
00407             will be used.
00408           - if \c mmffBond is supplied, a new singleton will be instantiated.
00409             The current instantiation (if there is one) will be deleted.
00410       */
00411       static MMFFBondCollection *getMMFFBond(const std::string &mmffBond = "");
00412       //! Looks up the parameters for a particular key and returns them.
00413       /*!
00414         \return a pointer to the MMFFBond object, NULL on failure.
00415       */
00416       const MMFFBond *operator()(const unsigned int bondType,
00417         const unsigned int atomType, const unsigned int nbrAtomType) {
00418         
00419         const MMFFBond *mmffBondParams = NULL;
00420         unsigned int canAtomType = atomType;
00421         unsigned int canNbrAtomType = nbrAtomType;
00422         if (atomType > nbrAtomType) {
00423           canAtomType = nbrAtomType;
00424           canNbrAtomType = atomType;
00425         }
00426         #ifdef RDKIT_MMFF_PARAMS_USE_STD_MAP
00427         std::map<const unsigned int, std::map<const unsigned int,
00428             std::map<const unsigned int, MMFFBond> > >::const_iterator res1;
00429         std::map<const unsigned int,
00430             std::map<const unsigned int, MMFFBond> >::const_iterator res2;
00431         std::map<const unsigned int, MMFFBond>::const_iterator res3;
00432         res1 = d_params.find(bondType);
00433         if (res1 != d_params.end()) {
00434           res2 = ((*res1).second).find(canAtomType);
00435           if (res2 != ((*res1).second).end()) {
00436             res3 = ((*res2).second).find(canNbrAtomType);
00437             if (res3 != ((*res2).second).end()) {
00438               mmffBondParams = &((*res3).second);
00439             }
00440           }
00441         }
00442         #else
00443         std::pair<std::vector<boost::uint8_t>::const_iterator,
00444           std::vector<boost::uint8_t>::const_iterator> bounds;
00445         bounds = std::equal_range(d_iAtomType.begin(), d_iAtomType.end(), canAtomType);
00446         if (bounds.first != bounds.second) {
00447           bounds = std::equal_range(d_jAtomType.begin() + (bounds.first - d_iAtomType.begin()),
00448             d_jAtomType.begin() + (bounds.second - d_iAtomType.begin()), canNbrAtomType);
00449           if (bounds.first != bounds.second) {
00450               bounds = std::equal_range
00451                 (d_bondType.begin() + (bounds.first - d_jAtomType.begin()),
00452                 d_bondType.begin() + (bounds.second - d_jAtomType.begin()), bondType);
00453             if (bounds.first != bounds.second) {
00454               mmffBondParams = &d_params[bounds.first - d_bondType.begin()];
00455             }
00456           }
00457         }
00458         #endif
00459 
00460         return mmffBondParams;
00461       }
00462     private:
00463       //! to force this to be a singleton, the constructor must be private
00464       MMFFBondCollection(std::string mmffBond);
00465       static class MMFFBondCollection *ds_instance;    //!< the singleton
00466       #ifdef RDKIT_MMFF_PARAMS_USE_STD_MAP
00467       std::map<const unsigned int, std::map<const unsigned int,
00468         std::map<const unsigned int, MMFFBond> > > d_params;  //!< the parameter 3D-map
00469       #else
00470       std::vector<MMFFBond> d_params;  //!< the parameter vector
00471       std::vector<boost::uint8_t> d_iAtomType;  //! atom type vector for atom i
00472       std::vector<boost::uint8_t> d_jAtomType;  //! atom type vector for atom j
00473       std::vector<boost::uint8_t> d_bondType;  //! bond type vector for bond i-j
00474       #endif
00475     };
00476 
00477     class MMFFBndkCollection {
00478     public:
00479       //! gets a pointer to the singleton MMFFBndkCollection
00480       /*!
00481         \param mmffBndk (optional) a string with parameter data. See
00482          below for more information about this argument
00483 
00484         \return a pointer to the singleton MMFFBndkCollection
00485 
00486         <b>Notes:</b>
00487           - do <b>not</b> delete the pointer returned here
00488           - if the singleton MMFFBndkCollection has already been instantiated and
00489             \c mmffBndk is empty, the singleton will be returned.
00490           - if \c mmffBndk is empty and the singleton MMFFBndkCollection has
00491             not yet been instantiated, the default parameters (from Params.cpp)
00492             will be used.
00493           - if \c mmffBndk is supplied, a new singleton will be instantiated.
00494             The current instantiation (if there is one) will be deleted.
00495       */
00496       static MMFFBndkCollection *getMMFFBndk(const std::string &mmffBndk = "");
00497       //! Looks up the parameters for a particular key and returns them.
00498       /*!
00499         \return a pointer to the MMFFBndk object, NULL on failure.
00500       */
00501       const MMFFBond *operator()(const int atomicNum, const int nbrAtomicNum) {
00502         
00503         const MMFFBond *mmffBndkParams = NULL;
00504         unsigned int canAtomicNum = atomicNum;
00505         unsigned int canNbrAtomicNum = nbrAtomicNum;
00506         if (atomicNum > nbrAtomicNum) {
00507           canAtomicNum = nbrAtomicNum;
00508           canNbrAtomicNum = atomicNum;
00509         }
00510         #ifdef RDKIT_MMFF_PARAMS_USE_STD_MAP
00511         std::map<const unsigned int,
00512           std::map<const unsigned int, MMFFBond> >::const_iterator res1;
00513         std::map<const unsigned int, MMFFBond>::const_iterator res2;
00514         res1 = d_params.find(canAtomicNum);
00515         if (res1 != d_params.end()) {
00516           res2 = ((*res1).second).find(canNbrAtomicNum);
00517           if (res2 != ((*res1).second).end()) {
00518             mmffBndkParams = &((*res2).second);
00519           }
00520         }
00521         #else
00522         std::pair<std::vector<boost::uint8_t>::const_iterator,
00523           std::vector<boost::uint8_t>::const_iterator> bounds;
00524         bounds = std::equal_range
00525           (d_iAtomicNum.begin(), d_iAtomicNum.end(), canAtomicNum);
00526         if (bounds.first != bounds.second) {
00527           bounds = std::equal_range
00528             (d_jAtomicNum.begin() + (bounds.first - d_iAtomicNum.begin()),
00529             d_jAtomicNum.begin() + (bounds.second - d_iAtomicNum.begin()),
00530             canNbrAtomicNum);
00531           if (bounds.first != bounds.second) {
00532             mmffBndkParams = &d_params[bounds.first - d_jAtomicNum.begin()];
00533           }
00534         }
00535         #endif
00536 
00537         return mmffBndkParams;
00538       }
00539     private:
00540       //! to force this to be a singleton, the constructor must be private
00541       MMFFBndkCollection(std::string mmffBndk);
00542       static class MMFFBndkCollection *ds_instance;    //!< the singleton
00543       #ifdef RDKIT_MMFF_PARAMS_USE_STD_MAP
00544       std::map<const unsigned int, std::map<const unsigned int, MMFFBond> > d_params;  //!< the parameter 2D-map
00545       #else
00546       std::vector<MMFFBond> d_params;  //!< the parameter vector
00547       std::vector<boost::uint8_t> d_iAtomicNum;  //! atomic number vector for atom i
00548       std::vector<boost::uint8_t> d_jAtomicNum;  //! atomic number vector for atom j
00549       #endif
00550     };
00551 
00552     class MMFFCovRadPauEleCollection {
00553     public:
00554       //! gets a pointer to the singleton MMFFCovRadPauEleCollection
00555       /*!
00556         \param mmffCovRadPauEle (optional) a string with parameter data. See
00557          below for more information about this argument
00558 
00559         \return a pointer to the singleton MMFFCovRadPauEleCollection
00560 
00561         <b>Notes:</b>
00562           - do <b>not</b> delete the pointer returned here
00563           - if the singleton MMFFCovRadPauEleCollection has already been instantiated and
00564             \c mmffCovRadPauEle is empty, the singleton will be returned.
00565           - if \c mmffCovRadPauEle is empty and the singleton MMFFCovRadPauEleCollection has
00566             not yet been instantiated, the default parameters (from Params.cpp)
00567             will be used.
00568           - if \c mmffCovRadPauEle is supplied, a new singleton will be instantiated.
00569             The current instantiation (if there is one) will be deleted.
00570       */
00571       static MMFFCovRadPauEleCollection *getMMFFCovRadPauEle(const std::string &mmffCovRadPauEle = "");
00572       //! Looks up the parameters for a particular key and returns them.
00573       /*!
00574         \return a pointer to the MMFFCovRadPauEle object, NULL on failure.
00575       */
00576       const MMFFCovRadPauEle *operator()(const unsigned int atomicNum) const {
00577         #ifdef RDKIT_MMFF_PARAMS_USE_STD_MAP
00578         std::map<const unsigned int, MMFFCovRadPauEle>::const_iterator res;
00579         res = d_params.find(atomicNum);
00580 
00581         return ((res != d_params.end()) ? &((*res).second) : NULL);
00582         #else
00583         std::pair<std::vector<boost::uint8_t>::const_iterator,
00584           std::vector<boost::uint8_t>::const_iterator> bounds =
00585           std::equal_range(d_atomicNum.begin(), d_atomicNum.end(), atomicNum);
00586 
00587         return ((bounds.first != bounds.second)
00588           ? &d_params[bounds.first - d_atomicNum.begin()] : NULL);
00589         #endif
00590       }
00591     private:
00592       //! to force this to be a singleton, the constructor must be private
00593       MMFFCovRadPauEleCollection(std::string mmffCovRadPauEle);
00594       static class MMFFCovRadPauEleCollection *ds_instance;    //!< the singleton
00595       #ifdef RDKIT_MMFF_PARAMS_USE_STD_MAP
00596       std::map<const unsigned int, MMFFCovRadPauEle> d_params;  //!< the parameter map
00597       #else
00598       std::vector<MMFFCovRadPauEle> d_params;  //!< the parameter vector
00599       std::vector<boost::uint8_t> d_atomicNum;  //!< the atomic number vector
00600       #endif
00601     };
00602 
00603     class MMFFAngleCollection {
00604     public:
00605       //! gets a pointer to the singleton MMFFAngleCollection
00606       /*!
00607         \param mmffAngle (optional) a string with parameter data. See
00608          below for more information about this argument
00609 
00610         \return a pointer to the singleton MMFFAngleCollection
00611 
00612         <b>Notes:</b>
00613           - do <b>not</b> delete the pointer returned here
00614           - if the singleton MMFFAngleCollection has already been instantiated and
00615             \c mmffAngle is empty, the singleton will be returned.
00616           - if \c mmffAngle is empty and the singleton MMFFAngleCollection has
00617             not yet been instantiated, the default parameters (from Params.cpp)
00618             will be used.
00619           - if \c mmffAngle is supplied, a new singleton will be instantiated.
00620             The current instantiation (if there is one) will be deleted.
00621       */
00622       static MMFFAngleCollection *getMMFFAngle(const std::string &mmffAngle = "");
00623       //! Looks up the parameters for a particular key and returns them.
00624       /*!
00625         \return a pointer to the MMFFAngle object, NULL on failure.
00626       */
00627       const MMFFAngle *operator()(const unsigned int angleType,
00628         const unsigned int iAtomType, const unsigned int jAtomType,
00629         const unsigned int kAtomType ) {
00630 
00631         MMFFDefCollection *mmffDef = MMFFDefCollection::getMMFFDef();
00632         const MMFFAngle *mmffAngleParams = NULL;
00633         unsigned int iter = 0;
00634 
00635         // For bending of the i-j-k angle, a five-stage process based
00636         // in the level combinations 1-1-1,2-2-2,3-2-3,4-2-4, and
00637         // 5-2-5 is used. (MMFF.I, note 68, page 519)
00638         // We skip 1-1-1 since Level 2 === Level 1
00639         #ifdef RDKIT_MMFF_PARAMS_USE_STD_MAP
00640         std::map<const unsigned int, std::map<const unsigned int, std::map<const unsigned int,
00641             std::map<const unsigned int, MMFFAngle> > > >::const_iterator res1;
00642         std::map<const unsigned int, std::map<const unsigned int, std::map<const unsigned int,
00643             MMFFAngle> > >::const_iterator res2;
00644         std::map<const unsigned int, std::map<const unsigned int, MMFFAngle> >::const_iterator res3;
00645         std::map<const unsigned int, MMFFAngle>::const_iterator res4;
00646         while ((iter < 4) && (!mmffAngleParams)) {
00647           unsigned int canIAtomType = (*mmffDef)(iAtomType)->eqLevel[iter];
00648           unsigned int canKAtomType = (*mmffDef)(kAtomType)->eqLevel[iter];
00649           if (canIAtomType > canKAtomType) {
00650             unsigned int temp = canKAtomType;
00651             canKAtomType = canIAtomType;
00652             canIAtomType = temp;
00653           }
00654           res1 = d_params.find(angleType);
00655           if (res1 != d_params.end()) {
00656             res2 = ((*res1).second).find(canIAtomType);
00657             if (res2 != ((*res1).second).end()) {
00658               res3 = ((*res2).second).find(jAtomType);
00659               if (res3 != ((*res2).second).end()) {
00660                 res4 = ((*res3).second).find(canKAtomType);
00661                 if (res4 != ((*res3).second).end()) {
00662                   mmffAngleParams = &((*res4).second);
00663                 }
00664               }
00665             }
00666           }
00667           ++iter;
00668         }
00669         #else
00670         std::pair<std::vector<boost::uint8_t>::const_iterator,
00671             std::vector<boost::uint8_t>::const_iterator> jBounds =
00672           std::equal_range(d_jAtomType.begin(), d_jAtomType.end(), jAtomType);
00673         std::pair<std::vector<boost::uint8_t>::const_iterator,
00674           std::vector<boost::uint8_t>::const_iterator> bounds;
00675         if (jBounds.first != jBounds.second) {
00676           while ((iter < 4) && (!mmffAngleParams)) {
00677             unsigned int canIAtomType = (*mmffDef)(iAtomType)->eqLevel[iter];
00678             unsigned int canKAtomType = (*mmffDef)(kAtomType)->eqLevel[iter];
00679             if (canIAtomType > canKAtomType) {
00680               unsigned int temp = canKAtomType;
00681               canKAtomType = canIAtomType;
00682               canIAtomType = temp;
00683             }
00684             bounds = std::equal_range
00685               (d_iAtomType.begin() + (jBounds.first - d_jAtomType.begin()),
00686               d_iAtomType.begin() + (jBounds.second - d_jAtomType.begin()),
00687               canIAtomType);
00688             if (bounds.first != bounds.second) {
00689               bounds = std::equal_range
00690                 (d_kAtomType.begin() + (bounds.first - d_iAtomType.begin()),
00691                 d_kAtomType.begin() + (bounds.second - d_iAtomType.begin()),
00692                 canKAtomType);
00693               if (bounds.first != bounds.second) {
00694                 bounds = std::equal_range
00695                   (d_angleType.begin() + (bounds.first - d_kAtomType.begin()),
00696                   d_angleType.begin() + (bounds.second - d_kAtomType.begin()), angleType);
00697                 if (bounds.first != bounds.second) {
00698                   mmffAngleParams = &d_params[bounds.first - d_angleType.begin()];
00699                 }
00700               }
00701             }
00702             ++iter;
00703           }
00704         }
00705         #endif
00706 
00707         return mmffAngleParams;
00708       }
00709     private:
00710       //! to force this to be a singleton, the constructor must be private
00711       MMFFAngleCollection(std::string mmffAngle);
00712       static class MMFFAngleCollection *ds_instance;    //!< the singleton
00713       #ifdef RDKIT_MMFF_PARAMS_USE_STD_MAP
00714       std::map<const unsigned int, std::map<const unsigned int,
00715         std::map<const unsigned int, std::map<const unsigned int, MMFFAngle> > > > d_params;  //!< the parameter 4D-map
00716       #else
00717       std::vector<MMFFAngle> d_params;  //!< the parameter vector
00718       std::vector<boost::uint8_t> d_iAtomType;  //! atom type vector for atom i
00719       std::vector<boost::uint8_t> d_jAtomType;  //! atom type vector for atom j
00720       std::vector<boost::uint8_t> d_kAtomType;  //! atom type vector for atom k
00721       std::vector<boost::uint8_t> d_angleType;  //! angle type vector for angle i-j-k
00722       #endif
00723     };
00724 
00725     class MMFFStbnCollection {
00726     public:
00727       //! gets a pointer to the singleton MMFFStbnCollection
00728       /*!
00729         \param mmffStbn (optional) a string with parameter data. See
00730          below for more information about this argument
00731 
00732         \return a pointer to the singleton MMFFStbnCollection
00733 
00734         <b>Notes:</b>
00735           - do <b>not</b> delete the pointer returned here
00736           - if the singleton MMFFStbnCollection has already been instantiated and
00737             \c mmffStbn is empty, the singleton will be returned.
00738           - if \c mmffStbn is empty and the singleton MMFFStbnCollection has
00739             not yet been instantiated, the default parameters (from Params.cpp)
00740             will be used.
00741           - if \c mmffStbn is supplied, a new singleton will be instantiated.
00742             The current instantiation (if there is one) will be deleted.
00743       */
00744       static MMFFStbnCollection *getMMFFStbn(const std::string &mmffStbn = "");
00745       //! Looks up the parameters for a particular key and returns them.
00746       /*!
00747         \return a pointer to the MMFFStbn object, NULL on failure.
00748       */
00749       const std::pair<bool, const MMFFStbn *> getMMFFStbnParams
00750         (const unsigned int stretchBendType, const unsigned int bondType1,
00751         const unsigned int bondType2, const unsigned int iAtomType,
00752         const unsigned int jAtomType, const unsigned int kAtomType) {
00753 
00754         const MMFFStbn *mmffStbnParams = NULL;
00755         bool swap = false;
00756         unsigned int canIAtomType = iAtomType;
00757         unsigned int canKAtomType = kAtomType;
00758         unsigned int canStretchBendType = stretchBendType;
00759         if (iAtomType > kAtomType) {
00760           canIAtomType = kAtomType;
00761           canKAtomType = iAtomType;
00762           swap = true;
00763         }
00764         else if (iAtomType == kAtomType) {
00765           swap = (bondType1 < bondType2);
00766         }
00767         #ifdef RDKIT_MMFF_PARAMS_USE_STD_MAP
00768         std::map<const unsigned int, std::map<const unsigned int, std::map<const unsigned int,
00769             std::map<const unsigned int, MMFFStbn> > > >::const_iterator res1;
00770         std::map<const unsigned int, std::map<const unsigned int, std::map<const unsigned int,
00771             MMFFStbn> > >::const_iterator res2;
00772         std::map<const unsigned int, std::map<const unsigned int, MMFFStbn> >::const_iterator res3;
00773         std::map<const unsigned int, MMFFStbn>::const_iterator res4;
00774         res1 = d_params.find(canStretchBendType);
00775         if (res1 != d_params.end()) {
00776           res2 = ((*res1).second).find(canIAtomType);
00777           if (res2 != ((*res1).second).end()) {
00778             res3 = ((*res2).second).find(jAtomType);
00779             if (res3 != ((*res2).second).end()) {
00780               res4 = ((*res3).second).find(canKAtomType);
00781               if (res4 != ((*res3).second).end()) {
00782                 mmffStbnParams = &((*res4).second);
00783               }
00784             }
00785           }
00786         }
00787         #else
00788         std::pair<std::vector<boost::uint8_t>::const_iterator,
00789             std::vector<boost::uint8_t>::const_iterator> jBounds =
00790           std::equal_range(d_jAtomType.begin(), d_jAtomType.end(), jAtomType);
00791         std::pair<std::vector<boost::uint8_t>::const_iterator,
00792           std::vector<boost::uint8_t>::const_iterator> bounds;
00793         if (jBounds.first != jBounds.second) {
00794           bounds = std::equal_range
00795             (d_iAtomType.begin() + (jBounds.first - d_jAtomType.begin()),
00796             d_iAtomType.begin() + (jBounds.second - d_jAtomType.begin()),
00797             canIAtomType);
00798           if (bounds.first != bounds.second) {
00799             bounds = std::equal_range
00800               (d_kAtomType.begin() + (bounds.first - d_iAtomType.begin()),
00801               d_kAtomType.begin() + (bounds.second - d_iAtomType.begin()),
00802               canKAtomType);
00803             if (bounds.first != bounds.second) {
00804               bounds = std::equal_range
00805                 (d_stretchBendType.begin() + (bounds.first - d_kAtomType.begin()),
00806                 d_stretchBendType.begin() + (bounds.second - d_kAtomType.begin()),
00807                 canStretchBendType);
00808               if (bounds.first != bounds.second) {
00809                 mmffStbnParams = &d_params[bounds.first - d_stretchBendType.begin()];
00810               }
00811             }
00812           }
00813         }
00814         #endif
00815         
00816         return std::make_pair(swap, mmffStbnParams);
00817       }
00818     private:
00819       //! to force this to be a singleton, the constructor must be private
00820       MMFFStbnCollection(std::string mmffStbn);
00821       static class MMFFStbnCollection *ds_instance;    //!< the singleton
00822       #ifdef RDKIT_MMFF_PARAMS_USE_STD_MAP
00823       std::map<const unsigned int, std::map<const unsigned int,
00824         std::map<const unsigned int, std::map<const unsigned int, MMFFStbn> > > > d_params;  //!< the parameter 4D-map
00825       #else
00826       std::vector<MMFFStbn> d_params;  //!< the parameter vector
00827       std::vector<boost::uint8_t> d_iAtomType;  //! atom type vector for atom i
00828       std::vector<boost::uint8_t> d_jAtomType;  //! atom type vector for atom j
00829       std::vector<boost::uint8_t> d_kAtomType;  //! atom type vector for atom k
00830       std::vector<boost::uint8_t> d_stretchBendType;  //! stretch-bend type vector for angle i-j-k
00831       #endif
00832     };
00833 
00834     class MMFFDfsbCollection {
00835     public:
00836       //! gets a pointer to the singleton MMFFDfsbCollection
00837       /*!
00838         \param mmffDfsb (optional) a string with parameter data. See
00839          below for more information about this argument
00840 
00841         \return a pointer to the singleton MMFFDfsbCollection
00842 
00843         <b>Notes:</b>
00844           - do <b>not</b> delete the pointer returned here
00845           - if the singleton MMFFDfsbCollection has already been instantiated and
00846             \c mmffDfsb is empty, the singleton will be returned.
00847           - if \c mmffDfsb is empty and the singleton MMFFDfsbCollection has
00848             not yet been instantiated, the default parameters (from Params.cpp)
00849             will be used.
00850           - if \c mmffDfsb is supplied, a new singleton will be instantiated.
00851             The current instantiation (if there is one) will be deleted.
00852       */
00853       static MMFFDfsbCollection *getMMFFDfsb(const std::string &mmffDfsb = "");
00854       //! Looks up the parameters for a particular key and returns them.
00855       /*!
00856         \return a pointer to the MMFFStbn object, NULL on failure.
00857       */
00858       const std::pair<bool, const MMFFStbn *> getMMFFDfsbParams(const unsigned int periodicTableRow1,
00859         const unsigned int periodicTableRow2, const unsigned int periodicTableRow3) {
00860 
00861         std::map<const unsigned int, std::map<const unsigned int, std::map<const unsigned int,
00862             MMFFStbn> > >::const_iterator res1;
00863         std::map<const unsigned int, std::map<const unsigned int, MMFFStbn> >::const_iterator res2;
00864         std::map<const unsigned int, MMFFStbn>::const_iterator res3;
00865         const MMFFStbn *mmffDfsbParams = NULL;
00866         bool swap = false;
00867         unsigned int canPeriodicTableRow1 = periodicTableRow1;
00868         unsigned int canPeriodicTableRow3 = periodicTableRow3;
00869         if (periodicTableRow1 > periodicTableRow3) {
00870           canPeriodicTableRow1 = periodicTableRow3;
00871           canPeriodicTableRow3 = periodicTableRow1;
00872           swap = true;
00873         }
00874         res1 = d_params.find(canPeriodicTableRow1);
00875         if (res1 != d_params.end()) {
00876           res2 = ((*res1).second).find(periodicTableRow2);
00877           if (res2 != ((*res1).second).end()) {
00878             res3 = ((*res2).second).find(canPeriodicTableRow3);
00879             if (res3 != ((*res2).second).end()) {
00880               mmffDfsbParams = &((*res3).second);
00881             }
00882           }
00883         }
00884         
00885         return std::make_pair(swap, mmffDfsbParams);
00886       }
00887     private:
00888       //! to force this to be a singleton, the constructor must be private
00889       MMFFDfsbCollection(std::string mmffDfsb);
00890       static class MMFFDfsbCollection *ds_instance;    //!< the singleton
00891       std::map<const unsigned int, std::map<const unsigned int,
00892         std::map<const unsigned int, MMFFStbn> > > d_params;  //!< the parameter 3D-map
00893     };
00894 
00895     class MMFFOopCollection {
00896     public:
00897       //! gets a pointer to the singleton MMFFOopCollection
00898       /*!
00899         \param mmffOop (optional) a string with parameter data. See
00900          below for more information about this argument
00901 
00902         \return a pointer to the singleton MMFFOopCollection
00903 
00904         <b>Notes:</b>
00905           - do <b>not</b> delete the pointer returned here
00906           - if the singleton MMFFOopCollection has already been instantiated and
00907             \c mmffOop is empty, the singleton will be returned.
00908           - if \c mmffOop is empty and the singleton MMFFOopCollection has
00909             not yet been instantiated, the default parameters (from Params.cpp)
00910             will be used.
00911           - if \c mmffOop is supplied, a new singleton will be instantiated.
00912             The current instantiation (if there is one) will be deleted.
00913       */
00914       static MMFFOopCollection *getMMFFOop
00915         (const bool isMMFFs = false, const std::string &mmffOop = "");
00916       //! Looks up the parameters for a particular key and returns them.
00917       /*!
00918         \return a pointer to the MMFFOop object, NULL on failure.
00919       */
00920       const MMFFOop *operator()(const unsigned int iAtomType, const unsigned int jAtomType,
00921         const unsigned int kAtomType, const unsigned int lAtomType) {
00922 
00923         MMFFDefCollection *mmffDef = MMFFDefCollection::getMMFFDef();
00924         const MMFFOop *mmffOopParams = NULL;
00925         unsigned int iter = 0;
00926         std::vector<unsigned int> canIKLAtomType(3);
00927         // For out-of-plane bending ijk; I , where j is the central
00928         // atom [cf. eq. (511, the five-stage protocol 1-1-1; 1, 2-2-2; 2,
00929         // 3-2-3;3, 4-2-4;4, 5-2-5;5 is used. The final stage provides
00930         // wild-card defaults for all except the central atom.
00931         #ifdef RDKIT_MMFF_PARAMS_USE_STD_MAP
00932         std::map<const unsigned int, std::map<const unsigned int, std::map<const unsigned int,
00933             std::map<const unsigned int, MMFFOop> > > >::const_iterator res1;
00934         std::map<const unsigned int, std::map<const unsigned int, std::map<const unsigned int,
00935             MMFFOop> > >::const_iterator res2;
00936         std::map<const unsigned int, std::map<const unsigned int, MMFFOop> >::const_iterator res3;
00937         std::map<const unsigned int, MMFFOop>::const_iterator res4;
00938         while ((iter < 4) && (!mmffOopParams)) {
00939           canIKLAtomType[0] = (*mmffDef)(iAtomType)->eqLevel[iter];
00940           unsigned int canJAtomType = jAtomType;
00941           canIKLAtomType[1] = (*mmffDef)(kAtomType)->eqLevel[iter];
00942           canIKLAtomType[2] = (*mmffDef)(lAtomType)->eqLevel[iter];
00943           std::sort(canIKLAtomType.begin(), canIKLAtomType.end());
00944           res1 = d_params.find(canIKLAtomType[0]);
00945           if (res1 != d_params.end()) {
00946             res2 = ((*res1).second).find(canJAtomType);
00947             if (res2 != ((*res1).second).end()) {
00948               res3 = ((*res2).second).find(canIKLAtomType[1]);
00949               if (res3 != ((*res2).second).end()) {
00950                 res4 = ((*res3).second).find(canIKLAtomType[2]);
00951                 if (res4 != ((*res3).second).end()) {
00952                   mmffOopParams = &((*res4).second);
00953                 }
00954               }
00955             }
00956           }
00957           ++iter;
00958         }
00959         #else
00960         std::pair<std::vector<boost::uint8_t>::const_iterator,
00961           std::vector<boost::uint8_t>::const_iterator> jBounds =
00962           std::equal_range(d_jAtomType.begin(), d_jAtomType.end(), jAtomType);
00963         std::pair<std::vector<boost::uint8_t>::const_iterator,
00964           std::vector<boost::uint8_t>::const_iterator> bounds;
00965         if (jBounds.first != jBounds.second) {
00966           while ((iter < 4) && (!mmffOopParams)) {
00967             canIKLAtomType[0] = (*mmffDef)(iAtomType)->eqLevel[iter];
00968             canIKLAtomType[1] = (*mmffDef)(kAtomType)->eqLevel[iter];
00969             canIKLAtomType[2] = (*mmffDef)(lAtomType)->eqLevel[iter];
00970             std::sort(canIKLAtomType.begin(), canIKLAtomType.end());
00971             bounds = std::equal_range
00972               (d_iAtomType.begin() + (jBounds.first - d_jAtomType.begin()),
00973               d_iAtomType.begin() + (jBounds.second - d_jAtomType.begin()),
00974               canIKLAtomType[0]);
00975             if (bounds.first != bounds.second) {
00976               bounds = std::equal_range
00977                 (d_kAtomType.begin() + (bounds.first - d_iAtomType.begin()),
00978                 d_kAtomType.begin() + (bounds.second - d_iAtomType.begin()),
00979                 canIKLAtomType[1]);
00980               if (bounds.first != bounds.second) {
00981                 bounds = std::equal_range
00982                   (d_lAtomType.begin() + (bounds.first - d_kAtomType.begin()),
00983                   d_lAtomType.begin() + (bounds.second - d_kAtomType.begin()),
00984                   canIKLAtomType[2]);
00985                 if (bounds.first != bounds.second) {
00986                   mmffOopParams = &d_params[bounds.first - d_lAtomType.begin()];
00987                 }
00988               }
00989             }
00990             ++iter;
00991           }
00992         }
00993         #endif
00994         
00995         return mmffOopParams;
00996       }
00997     private:
00998       //! to force this to be a singleton, the constructor must be private
00999       MMFFOopCollection(const bool isMMFFs, std::string mmffOop);
01000       static class MMFFOopCollection *ds_instance[2];    //!< the singleton
01001       #ifdef RDKIT_MMFF_PARAMS_USE_STD_MAP
01002       std::map<const unsigned int, std::map<const unsigned int,
01003         std::map<const unsigned int, std::map<const unsigned int, MMFFOop> > > > d_params;  //!< the parameter 4D-map
01004       #else
01005       std::vector<MMFFOop> d_params;  //!< the parameter vector
01006       std::vector<boost::uint8_t> d_iAtomType;  //! atom type vector for atom i
01007       std::vector<boost::uint8_t> d_jAtomType;  //! atom type vector for atom j
01008       std::vector<boost::uint8_t> d_kAtomType;  //! atom type vector for atom k
01009       std::vector<boost::uint8_t> d_lAtomType;  //! atom type vector for atom l
01010       #endif
01011     };
01012 
01013     class MMFFTorCollection {
01014     public:
01015       //! gets a pointer to the singleton MMFFTorCollection
01016       /*!
01017         \param mmffTor (optional) a string with parameter data. See
01018          below for more information about this argument
01019 
01020         \return a pointer to the singleton MMFFTorCollection
01021 
01022         <b>Notes:</b>
01023           - do <b>not</b> delete the pointer returned here
01024           - if the singleton MMFFTorCollection has already been instantiated and
01025             \c mmffTor is empty, the singleton will be returned.
01026           - if \c mmffTor is empty and the singleton MMFFTorCollection has
01027             not yet been instantiated, the default parameters (from Params.cpp)
01028             will be used.
01029           - if \c mmffTor is supplied, a new singleton will be instantiated.
01030             The current instantiation (if there is one) will be deleted.
01031       */
01032       static MMFFTorCollection *getMMFFTor(const bool isMMFFs, const std::string &mmffTor = "");
01033       //! Looks up the parameters for a particular key and returns them.
01034       /*!
01035         \return a pointer to the MMFFTor object, NULL on failure.
01036       */
01037       const std::pair<const unsigned int, const MMFFTor *> getMMFFTorParams
01038         (const std::pair<unsigned int, unsigned int> torType,
01039         const unsigned int iAtomType, const unsigned int jAtomType,
01040         const unsigned int kAtomType, const unsigned int lAtomType)
01041       {
01042         MMFFDefCollection *mmffDef = MMFFDefCollection::getMMFFDef();
01043         const MMFFTor *mmffTorParams = NULL;
01044         unsigned int iter = 0;
01045         unsigned int iWildCard = 0;
01046         unsigned int lWildCard = 0;
01047         unsigned int canTorType = torType.first;
01048         unsigned int maxIter = 5;
01049         // For i-j-k-2 torsion interactions, a five-stage
01050         // process based on level combinations 1-1-1-1, 2-2-2-2,
01051         // 3-2-2-5, 5-2-2-3, and 5-2-2-5 is used, where stages 3
01052         // and 4 correspond to "half-default" or "half-wild-card" entries.
01053         #ifdef RDKIT_MMFF_PARAMS_USE_STD_MAP
01054         std::map<const unsigned int,
01055           std::map<const unsigned int, std::map<const unsigned int,
01056           std::map<const unsigned int, std::map<const unsigned int,
01057           MMFFTor> > > > >::const_iterator res1;
01058         std::map<const unsigned int,
01059           std::map<const unsigned int, std::map<const unsigned int,
01060           std::map<const unsigned int, MMFFTor> > > >::const_iterator res2;
01061         std::map<const unsigned int,
01062           std::map<const unsigned int, std::map<const unsigned int,
01063           MMFFTor> > >::const_iterator res3;
01064         std::map<const unsigned int,
01065           std::map<const unsigned int, MMFFTor> >::const_iterator res4;
01066         std::map<const unsigned int, MMFFTor>::const_iterator res5;
01067         #else
01068         std::pair<std::vector<boost::uint8_t>::const_iterator,
01069             std::vector<boost::uint8_t>::const_iterator> jBounds;
01070         std::pair<std::vector<boost::uint8_t>::const_iterator,
01071           std::vector<boost::uint8_t>::const_iterator> bounds;
01072         #endif
01073         
01074         while (((iter < maxIter) && ((!mmffTorParams) || (maxIter == 4)))
01075           || ((iter == 4) && (torType.first == 5) && torType.second)) {
01076           // The rule of setting the torsion type to the value it had
01077           // before being set to 5 as a last resort in case parameters
01078           // could not be found is not mentioned in MMFF.IV; it was 
01079           // empirically discovered due to a number of tests in the
01080           // MMFF validation suite otherwise failing
01081           if ((maxIter == 5) && (iter == 4)) {
01082             maxIter = 4;
01083             iter = 0;
01084             canTorType = torType.second;
01085           }
01086           iWildCard = iter;
01087           lWildCard = iter;
01088           if (iter == 1) {
01089             iWildCard = 1;
01090             lWildCard = 3;
01091           }
01092           else if (iter == 2) {
01093             iWildCard = 3;
01094             lWildCard = 1;
01095           }
01096           unsigned int canIAtomType = (*mmffDef)(iAtomType)->eqLevel[iWildCard];
01097           unsigned int canJAtomType = jAtomType;
01098           unsigned int canKAtomType = kAtomType;
01099           unsigned int canLAtomType = (*mmffDef)(lAtomType)->eqLevel[lWildCard];
01100           if (canJAtomType > canKAtomType) {
01101             unsigned int temp = canKAtomType;
01102             canKAtomType = canJAtomType;
01103             canJAtomType = temp;
01104             temp = canLAtomType;
01105             canLAtomType = canIAtomType;
01106             canIAtomType = temp;
01107           }
01108           else if ((canJAtomType == canKAtomType)
01109             && (canIAtomType > canLAtomType)) {
01110             unsigned int temp = canLAtomType;
01111             canLAtomType = canIAtomType;
01112             canIAtomType = temp;
01113           }
01114           #ifdef RDKIT_MMFF_PARAMS_USE_STD_MAP
01115           res1 = d_params.find(canTorType);
01116           if (res1 != d_params.end()) {
01117             res2 = ((*res1).second).find(canIAtomType);
01118             if (res2 != ((*res1).second).end()) {
01119               res3 = ((*res2).second).find(canJAtomType);
01120               if (res3 != ((*res2).second).end()) {
01121                 res4 = ((*res3).second).find(canKAtomType);
01122                 if (res4 != ((*res3).second).end()) {
01123                   res5 = ((*res4).second).find(canLAtomType);
01124                   if (res5 != ((*res4).second).end()) {
01125                     mmffTorParams = &((*res5).second);
01126                     if (maxIter == 4) {
01127                       break;
01128                     }
01129                   }
01130                 }
01131               }
01132             }
01133           }
01134           #else
01135           jBounds = std::equal_range(d_jAtomType.begin(), d_jAtomType.end(), canJAtomType);
01136           if (jBounds.first != jBounds.second) {
01137             bounds = std::equal_range
01138               (d_kAtomType.begin() + (jBounds.first - d_jAtomType.begin()),
01139               d_kAtomType.begin() + (jBounds.second - d_jAtomType.begin()),
01140               canKAtomType);
01141             if (bounds.first != bounds.second) {
01142               bounds = std::equal_range
01143                 (d_iAtomType.begin() + (bounds.first - d_kAtomType.begin()),
01144                 d_iAtomType.begin() + (bounds.second - d_kAtomType.begin()),
01145                 canIAtomType);
01146               if (bounds.first != bounds.second) {
01147                 bounds = std::equal_range
01148                   (d_lAtomType.begin() + (bounds.first - d_iAtomType.begin()),
01149                   d_lAtomType.begin() + (bounds.second - d_iAtomType.begin()),
01150                   canLAtomType);
01151                 if (bounds.first != bounds.second) {
01152                   bounds = std::equal_range
01153                     (d_torType.begin() + (bounds.first - d_lAtomType.begin()),
01154                     d_torType.begin() + (bounds.second - d_lAtomType.begin()),
01155                     canTorType);
01156                   if (bounds.first != bounds.second) {
01157                     mmffTorParams = &d_params[bounds.first - d_torType.begin()];
01158                     if (maxIter == 4) {
01159                       break;
01160                     }
01161                   }
01162                 }
01163               }
01164             }
01165           }
01166           #endif
01167           ++iter;
01168         }
01169         
01170         return std::make_pair(canTorType, mmffTorParams);
01171       }
01172     private:
01173       //! to force this to be a singleton, the constructor must be private
01174       MMFFTorCollection(const bool isMMFFs, std::string mmffTor);
01175       static class MMFFTorCollection *ds_instance[2];    //!< the singleton
01176       #ifdef RDKIT_MMFF_PARAMS_USE_STD_MAP
01177       std::map<const unsigned int,
01178         std::map<const unsigned int, std::map<const unsigned int,
01179         std::map<const unsigned int, std::map<const unsigned int,
01180         MMFFTor> > > > > d_params;  //!< the parameter 5D-map
01181       #else
01182       std::vector<MMFFTor> d_params;  //!< the parameter vector
01183       std::vector<boost::uint8_t> d_iAtomType;  //! atom type vector for atom i
01184       std::vector<boost::uint8_t> d_jAtomType;  //! atom type vector for atom j
01185       std::vector<boost::uint8_t> d_kAtomType;  //! atom type vector for atom k
01186       std::vector<boost::uint8_t> d_lAtomType;  //! atom type vector for atom l
01187       std::vector<boost::uint8_t> d_torType;  //! torsion type vector for angle i-j-k-l
01188       #endif
01189     };
01190 
01191     class MMFFVdWCollection {
01192     public:
01193       //! gets a pointer to the singleton MMFFVdWCollection
01194       /*!
01195         \param mmffVdW (optional) a string with parameter data. See
01196          below for more information about this argument
01197 
01198         \return a pointer to the singleton MMFFVdWCollection
01199 
01200         <b>Notes:</b>
01201           - do <b>not</b> delete the pointer returned here
01202           - if the singleton MMFFVdWCollection has already been instantiated and
01203             \c mmffVdW is empty, the singleton will be returned.
01204           - if \c mmffVdW is empty and the singleton MMFFVdWCollection has
01205             not yet been instantiated, the default parameters (from Params.cpp)
01206             will be used.
01207           - if \c mmffVdW is supplied, a new singleton will be instantiated.
01208             The current instantiation (if there is one) will be deleted.
01209       */
01210       double power;
01211       double B;
01212       double Beta;
01213       double DARAD;
01214       double DAEPS;
01215       static MMFFVdWCollection *getMMFFVdW(const std::string &mmffVdW = "");
01216       //! Looks up the parameters for a particular key and returns them.
01217       /*!
01218         \return a pointer to the MMFFVdW object, NULL on failure.
01219       */
01220       const MMFFVdW *operator()(const unsigned int atomType) const {
01221         #ifdef RDKIT_MMFF_PARAMS_USE_STD_MAP
01222         std::map<const unsigned int, MMFFVdW>::const_iterator res;
01223         res = d_params.find(atomType);
01224 
01225         return (res != d_params.end() ? &((*res).second) : NULL);
01226         #else
01227         std::pair<std::vector<boost::uint8_t>::const_iterator,
01228           std::vector<boost::uint8_t>::const_iterator> bounds =
01229           std::equal_range(d_atomType.begin(), d_atomType.end(), atomType);
01230 
01231         return ((bounds.first != bounds.second)
01232           ? &d_params[bounds.first - d_atomType.begin()] : NULL);
01233         #endif
01234       }
01235     private:
01236       //! to force this to be a singleton, the constructor must be private
01237       MMFFVdWCollection(std::string mmffVdW);
01238       static class MMFFVdWCollection *ds_instance;    //!< the singleton
01239       #ifdef RDKIT_MMFF_PARAMS_USE_STD_MAP
01240       std::map<const unsigned int, MMFFVdW> d_params;  //!< the parameter map
01241       #else
01242       std::vector<MMFFVdW> d_params;  //!< the parameter vector
01243       std::vector<boost::uint8_t> d_atomType;  //! atom type vector
01244       #endif
01245     };
01246   }
01247 }
01248 
01249 #endif
```

---

Generated on 16 Feb 2014 for RDKit-MMFF by 
 1.6.1 
