# Supplementary material for: Bringing the MMFF force field to the RDKit: implementation and validation
Source: J Cheminform. 2014 Jul 12;6:37. doi: 10.1186/s13321-014-0037-3 (PMC4116604; doi:10.1186/s13321-014-0037-3)
Supplement: Additional file 3: — Documentation. The file docs.zip expands to an HTML tree which documents the MMFF-related C++ and Python RDKit APIs; the documentation can be browsed opening the docs.html file in any HTML browser. The full RDKit documentation can be found at http://www.rdkit.org. [file s13321-014-0037-3-S3.zip › docs/cpp/PositionConstraint_8h_source.html]

RDKit-MMFF: PositionConstraint.h Source File


- Main Page
- Namespaces
- Classes
- Files
- Directories

- File List
- File Members

ForceField » MMFF

# PositionConstraint.h

Go to the documentation of this file.

```
00001 //
00002 //  Copyright (C) 2013 Paolo Tosco
00003 //
00004 //  Copyright (C) 2004-2006 Rational Discovery LLC
00005 //
00006 //   @@ All Rights Reserved @@
00007 //  This file is part of the RDKit.
00008 //  The contents are covered by the terms of the BSD license
00009 //  which is included in the file license.txt, found at the root
00010 //  of the RDKit source tree.
00011 //
00012 #ifndef __RD_MMFFPOSITIONCONSTRAINT_H__
00013 #define __RD_MMFFPOSITIONCONSTRAINT_H__
00014 #include <iostream>
00015 #include <ForceField/Contrib.h>
00016 #include <Geometry/point.h>
00017 
00018 namespace ForceFields {
00019   namespace MMFF {
00020 
00021     //! A position constraint of the type 0.5k * deltaX^2
00022     class PositionConstraintContrib : public ForceFieldContrib {
00023     public:
00024       PositionConstraintContrib() : d_atIdx(-1) {};
00025       //! Constructor
00026       /*!
00027         \param owner       pointer to the owning ForceField
00028         \param idx         index of the atom in the ForceField's positions
00029         \param minDispl    minimum displacement
00030         \param maxDispl    maximum displacement
00031         \param forceConst  force constant
00032         
00033       */
00034       PositionConstraintContrib(ForceField *owner,
00035         unsigned int idx, double maxDispl, double forceConst);
00036 
00037       ~PositionConstraintContrib() {
00038       }
00039       double getEnergy(double *pos) const;
00040 
00041       void getGrad(double *pos, double *grad) const;
00042     private:
00043       int d_atIdx; //!< index of the restrained atom
00044       double d_maxDispl;  //!< maximum allowed displacement
00045       RDGeom::Point3D d_pos0;  //!< reference position
00046       double d_forceConstant;  //!< force constant of the bond
00047 
00048     };
00049   }
00050 }
00051 #endif
```

---

Generated on 16 Feb 2014 for RDKit-MMFF by 
 1.6.1 
