# Supplementary material for: Bringing the MMFF force field to the RDKit: implementation and validation
Source: J Cheminform. 2014 Jul 12;6:37. doi: 10.1186/s13321-014-0037-3 (PMC4116604; doi:10.1186/s13321-014-0037-3)
Supplement: Additional file 3: — Documentation. The file docs.zip expands to an HTML tree which documents the MMFF-related C++ and Python RDKit APIs; the documentation can be browsed opening the docs.html file in any HTML browser. The full RDKit documentation can be found at http://www.rdkit.org. [file s13321-014-0037-3-S3.zip › docs/cpp/search/all_61.html]

Loading...

A\_i
ForceFields::MMFF::MMFFVdW

addAngles
RDKit::MMFF::Tools

addBonds
RDKit::MMFF::Tools

addEle
RDKit::MMFF::Tools

addOop
RDKit::MMFF::Tools

addStretchBend
RDKit::MMFF::Tools

addTorsions
RDKit::MMFF::Tools

addVdW
RDKit::MMFF::Tools

alpha\_i
ForceFields::MMFF::MMFFVdW

AngleBend.h

AngleBendContrib

ForceFields::MMFF::AngleBendContrib::AngleBendContrib()
ForceFields::MMFF::AngleBendContrib::AngleBendContrib(ForceField \*owner, unsigned int idx1, unsigned int idx2, unsigned int idx3, const MMFFAngle \*mmffAngleParams, const MMFFProp \*mmffPropParamsCentralAtom)

AngleBendContrib
ForceFields::MMFF

AngleConstraint.h

AngleConstraintContrib

ForceFields::MMFF::AngleConstraintContrib::AngleConstraintContrib()
ForceFields::MMFF::AngleConstraintContrib::AngleConstraintContrib(ForceField \*owner, unsigned int idx1, unsigned int idx2, unsigned int idx3, double minAngleDeg, double maxAngleDeg, double forceConst)
ForceFields::MMFF::AngleConstraintContrib::AngleConstraintContrib(ForceField \*owner, unsigned int idx1, unsigned int idx2, unsigned int idx3, bool relative, double minAngleDeg, double maxAngleDeg, double forceConst)

AngleConstraintContrib
ForceFields::MMFF

areAtomsInSameAromaticRing
RDKit::MMFF

areAtomsInSameRingOfSize
RDKit::MMFF

arom
ForceFields::MMFF::MMFFProp

atno
ForceFields::MMFF::MMFFProp

AtomTyper.h

Searching...

No Matches
