# Supplementary material for: Bringing the MMFF force field to the RDKit: implementation and validation
Source: J Cheminform. 2014 Jul 12;6:37. doi: 10.1186/s13321-014-0037-3 (PMC4116604; doi:10.1186/s13321-014-0037-3)
Supplement: Additional file 3: — Documentation. The file docs.zip expands to an HTML tree which documents the MMFF-related C++ and Python RDKit APIs; the documentation can be browsed opening the docs.html file in any HTML browser. The full RDKit documentation can be found at http://www.rdkit.org. [file s13321-014-0037-3-S3.zip › docs/cpp/search/all_63.html]

Loading...

calcAngleBendEnergy
ForceFields::MMFF::Utils

calcAngleBendGrad
ForceFields::MMFF::Utils

calcAngleForceConstant
ForceFields::MMFF::Utils

calcAngleRestValue
ForceFields::MMFF::Utils

calcBondForceConstant
ForceFields::MMFF::Utils

calcBondRestLength
ForceFields::MMFF::Utils

calcBondStretchEnergy
ForceFields::MMFF::Utils

calcCosTheta
ForceFields::MMFF::Utils

calcEleEnergy
ForceFields::MMFF::Utils

calcOopBendEnergy
ForceFields::MMFF::Utils

calcOopBendForceConstant
ForceFields::MMFF::Utils

calcOopChi
ForceFields::MMFF::Utils

calcStbnForceConstants
ForceFields::MMFF::Utils

calcStretchBendEnergy
ForceFields::MMFF::Utils

calcTorsionCosPhi
ForceFields::MMFF::Utils

calcTorsionEnergy
ForceFields::MMFF::Utils

calcTorsionForceConstant
ForceFields::MMFF::Utils

calcTorsionGrad
ForceFields::MMFF::Utils

calcUnscaledVdWMinimum
ForceFields::MMFF::Utils

calcUnscaledVdWWellDepth
ForceFields::MMFF::Utils

calcVdWEnergy
ForceFields::MMFF::Utils

chi
ForceFields::MMFF::MMFFCovRadPauEle

computeMMFFCharges
RDKit::MMFF::MMFFMolProperties

CONSTANT
RDKit::MMFF

constructForceField

RDKit::MMFF::constructForceField(ROMol &mol, double nonBondedThresh=100.0, int confId=-1, bool ignoreInterfragInteractions=true)
RDKit::MMFF::constructForceField(ROMol &mol, MMFFMolProperties \*mmffMolProperties, double nonBondedThresh=100.0, int confId=-1, bool ignoreInterfragInteractions=true)

Contribs.h

crd
ForceFields::MMFF::MMFFProp

Searching...

No Matches
