# Supplementary material for: Bringing the MMFF force field to the RDKit: implementation and validation
Source: J Cheminform. 2014 Jul 12;6:37. doi: 10.1186/s13321-014-0037-3 (PMC4116604; doi:10.1186/s13321-014-0037-3)
Supplement: Additional file 3: — Documentation. The file docs.zip expands to an HTML tree which documents the MMFF-related C++ and Python RDKit APIs; the documentation can be browsed opening the docs.html file in any HTML browser. The full RDKit documentation can be found at http://www.rdkit.org. [file s13321-014-0037-3-S3.zip › docs/cpp/search/all_67.html]

Loading...

G\_i
ForceFields::MMFF::MMFFVdW

getEnergy

ForceFields::MMFF::AngleBendContrib::getEnergy()
ForceFields::MMFF::AngleConstraintContrib::getEnergy()
ForceFields::MMFF::BondStretchContrib::getEnergy()
ForceFields::MMFF::DistanceConstraintContrib::getEnergy()
ForceFields::MMFF::VdWContrib::getEnergy()
ForceFields::MMFF::EleContrib::getEnergy()
ForceFields::MMFF::OopBendContrib::getEnergy()
ForceFields::MMFF::PositionConstraintContrib::getEnergy()
ForceFields::MMFF::StretchBendContrib::getEnergy()
ForceFields::MMFF::TorsionAngleContrib::getEnergy()
ForceFields::MMFF::TorsionConstraintContrib::getEnergy()

getGrad

ForceFields::MMFF::AngleBendContrib::getGrad()
ForceFields::MMFF::AngleConstraintContrib::getGrad()
ForceFields::MMFF::BondStretchContrib::getGrad()
ForceFields::MMFF::DistanceConstraintContrib::getGrad()
ForceFields::MMFF::VdWContrib::getGrad()
ForceFields::MMFF::EleContrib::getGrad()
ForceFields::MMFF::OopBendContrib::getGrad()
ForceFields::MMFF::PositionConstraintContrib::getGrad()
ForceFields::MMFF::StretchBendContrib::getGrad()
ForceFields::MMFF::TorsionAngleContrib::getGrad()
ForceFields::MMFF::TorsionConstraintContrib::getGrad()

getMMFFAngle
ForceFields::MMFF::MMFFAngleCollection

getMMFFAngleBendEmpiricalRuleParams
RDKit::MMFF

getMMFFAngleTerm
RDKit::MMFF::MMFFMolProperties

getMMFFAngleType
RDKit::MMFF::MMFFMolProperties

getMMFFArom
ForceFields::MMFF::MMFFAromCollection

getMMFFAtomType
RDKit::MMFF::MMFFMolProperties

getMMFFBndk
ForceFields::MMFF::MMFFBndkCollection

getMMFFBond
ForceFields::MMFF::MMFFBondCollection

getMMFFBondStretchEmpiricalRuleParams
RDKit::MMFF::MMFFMolProperties

getMMFFBondTerm
RDKit::MMFF::MMFFMolProperties

getMMFFBondType
RDKit::MMFF::MMFFMolProperties

getMMFFChg
ForceFields::MMFF::MMFFChgCollection

getMMFFChgParams
ForceFields::MMFF::MMFFChgCollection

getMMFFCovRadPauEle
ForceFields::MMFF::MMFFCovRadPauEleCollection

getMMFFDef
ForceFields::MMFF::MMFFDefCollection

getMMFFDfsb
ForceFields::MMFF::MMFFDfsbCollection

getMMFFDfsbParams
ForceFields::MMFF::MMFFDfsbCollection

getMMFFDielectricConstant
RDKit::MMFF::MMFFMolProperties

getMMFFDielectricModel
RDKit::MMFF::MMFFMolProperties

getMMFFEleTerm
RDKit::MMFF::MMFFMolProperties

getMMFFFormalCharge
RDKit::MMFF::MMFFMolProperties

getMMFFOop
ForceFields::MMFF::MMFFOopCollection

getMMFFOopTerm
RDKit::MMFF::MMFFMolProperties

getMMFFOStream
RDKit::MMFF::MMFFMolProperties

getMMFFPartialCharge
RDKit::MMFF::MMFFMolProperties

getMMFFPBCI
ForceFields::MMFF::MMFFPBCICollection

getMMFFProp
ForceFields::MMFF::MMFFPropCollection

getMMFFStbn
ForceFields::MMFF::MMFFStbnCollection

getMMFFStbnParams
ForceFields::MMFF::MMFFStbnCollection

getMMFFStretchBendTerm
RDKit::MMFF::MMFFMolProperties

getMMFFStretchBendType
RDKit::MMFF

getMMFFTor
ForceFields::MMFF::MMFFTorCollection

getMMFFTorParams
ForceFields::MMFF::MMFFTorCollection

getMMFFTorsionEmpiricalRuleParams
RDKit::MMFF::MMFFMolProperties

getMMFFTorsionTerm
RDKit::MMFF::MMFFMolProperties

getMMFFTorsionType
RDKit::MMFF::MMFFMolProperties

getMMFFVariant
RDKit::MMFF::MMFFMolProperties

getMMFFVdW
ForceFields::MMFF::MMFFVdWCollection

getMMFFVdWTerm
RDKit::MMFF::MMFFMolProperties

getMMFFVerbosity
RDKit::MMFF::MMFFMolProperties

getPeriodicTableRow
RDKit::MMFF

getTwoBitCell
RDKit::MMFF::Tools

Searching...

No Matches
