# Supplementary material for: Bringing the MMFF force field to the RDKit: implementation and validation
Source: J Cheminform. 2014 Jul 12;6:37. doi: 10.1186/s13321-014-0037-3 (PMC4116604; doi:10.1186/s13321-014-0037-3)
Supplement: Additional file 3: — Documentation. The file docs.zip expands to an HTML tree which documents the MMFF-related C++ and Python RDKit APIs; the documentation can be browsed opening the docs.html file in any HTML browser. The full RDKit documentation can be found at http://www.rdkit.org. [file s13321-014-0037-3-S3.zip › docs/cpp/search/all_6b.html]

Loading...

ka
ForceFields::MMFF::MMFFAngle

kb
ForceFields::MMFF::MMFFBond

kbaIJK
ForceFields::MMFF::MMFFStbn

kbaKJI
ForceFields::MMFF::MMFFStbn

koop
ForceFields::MMFF::MMFFOop

Searching...

No Matches
