# Supplementary material for: Bringing the MMFF force field to the RDKit: implementation and validation
Source: J Cheminform. 2014 Jul 12;6:37. doi: 10.1186/s13321-014-0037-3 (PMC4116604; doi:10.1186/s13321-014-0037-3)
Supplement: Additional file 3: — Documentation. The file docs.zip expands to an HTML tree which documents the MMFF-related C++ and Python RDKit APIs; the documentation can be browsed opening the docs.html file in any HTML browser. The full RDKit documentation can be found at http://www.rdkit.org. [file s13321-014-0037-3-S3.zip › docs/cpp/search/all_6d.html]

Loading...

M\_PI
Params.h

mltb
ForceFields::MMFF::MMFFProp

MMFF\_VERBOSITY\_HIGH
RDKit::MMFF

MMFF\_VERBOSITY\_LOW
RDKit::MMFF

MMFF\_VERBOSITY\_NONE
RDKit::MMFF

MMFFAngle
ForceFields::MMFF

MMFFAngleCollection
ForceFields::MMFF

MMFFAromCollection
ForceFields::MMFF

MMFFAtomProperties
RDKit::MMFF

MMFFAtomProperties
RDKit::MMFF::MMFFAtomProperties

MMFFAtomPropertiesPtr
RDKit::MMFF

mmffAtomType
RDKit::MMFF::MMFFAtomProperties

MMFFBndkCollection
ForceFields::MMFF

MMFFBond
ForceFields::MMFF

MMFFBondCollection
ForceFields::MMFF

MMFFChg
ForceFields::MMFF

MMFFChgCollection
ForceFields::MMFF

MMFFCovRadPauEle
ForceFields::MMFF

MMFFCovRadPauEleCollection
ForceFields::MMFF

MMFFDef
ForceFields::MMFF

MMFFDefCollection
ForceFields::MMFF

MMFFDfsbCollection
ForceFields::MMFF

mmffFormalCharge
RDKit::MMFF::MMFFAtomProperties

MMFFMolProperties
RDKit::MMFF::MMFFMolProperties

MMFFMolProperties
RDKit::MMFF

MMFFOop
ForceFields::MMFF

MMFFOopCollection
ForceFields::MMFF

mmffPartialCharge
RDKit::MMFF::MMFFAtomProperties

MMFFPBCI
ForceFields::MMFF

MMFFPBCICollection
ForceFields::MMFF

MMFFProp
ForceFields::MMFF

MMFFPropCollection
ForceFields::MMFF

MMFFStbn
ForceFields::MMFF

MMFFStbnCollection
ForceFields::MMFF

MMFFTor
ForceFields::MMFF

MMFFTorCollection
ForceFields::MMFF

MMFFVdW
ForceFields::MMFF

MMFFVdWCollection
ForceFields::MMFF

Searching...

No Matches
