# Supplementary material for: Bringing the MMFF force field to the RDKit: implementation and validation
Source: J Cheminform. 2014 Jul 12;6:37. doi: 10.1186/s13321-014-0037-3 (PMC4116604; doi:10.1186/s13321-014-0037-3)
Supplement: Additional file 3: — Documentation. The file docs.zip expands to an HTML tree which documents the MMFF-related C++ and Python RDKit APIs; the documentation can be browsed opening the docs.html file in any HTML browser. The full RDKit documentation can be found at http://www.rdkit.org. [file s13321-014-0037-3-S3.zip › docs/cpp/search/functions_61.html]

Loading...

addAngles
RDKit::MMFF::Tools

addBonds
RDKit::MMFF::Tools

addEle
RDKit::MMFF::Tools

addOop
RDKit::MMFF::Tools

addStretchBend
RDKit::MMFF::Tools

addTorsions
RDKit::MMFF::Tools

addVdW
RDKit::MMFF::Tools

AngleBendContrib

ForceFields::MMFF::AngleBendContrib::AngleBendContrib()
ForceFields::MMFF::AngleBendContrib::AngleBendContrib(ForceField \*owner, unsigned int idx1, unsigned int idx2, unsigned int idx3, const MMFFAngle \*mmffAngleParams, const MMFFProp \*mmffPropParamsCentralAtom)

AngleConstraintContrib

ForceFields::MMFF::AngleConstraintContrib::AngleConstraintContrib()
ForceFields::MMFF::AngleConstraintContrib::AngleConstraintContrib(ForceField \*owner, unsigned int idx1, unsigned int idx2, unsigned int idx3, double minAngleDeg, double maxAngleDeg, double forceConst)
ForceFields::MMFF::AngleConstraintContrib::AngleConstraintContrib(ForceField \*owner, unsigned int idx1, unsigned int idx2, unsigned int idx3, bool relative, double minAngleDeg, double maxAngleDeg, double forceConst)

areAtomsInSameAromaticRing
RDKit::MMFF

areAtomsInSameRingOfSize
RDKit::MMFF

Searching...

No Matches
