# Supplementary material for: Bringing the MMFF force field to the RDKit: implementation and validation
Source: J Cheminform. 2014 Jul 12;6:37. doi: 10.1186/s13321-014-0037-3 (PMC4116604; doi:10.1186/s13321-014-0037-3)
Supplement: Additional file 3: — Documentation. The file docs.zip expands to an HTML tree which documents the MMFF-related C++ and Python RDKit APIs; the documentation can be browsed opening the docs.html file in any HTML browser. The full RDKit documentation can be found at http://www.rdkit.org. [file s13321-014-0037-3-S3.zip › docs/cpp/search/functions_73.html]

Loading...

sanitizeMMFFMol
RDKit::MMFF

scaleVdWParams
ForceFields::MMFF::Utils

setMMFFAngleTerm
RDKit::MMFF::MMFFMolProperties

setMMFFAromaticity
RDKit::MMFF

setMMFFBondTerm
RDKit::MMFF::MMFFMolProperties

setMMFFDielectricConstant
RDKit::MMFF::MMFFMolProperties

setMMFFDielectricModel
RDKit::MMFF::MMFFMolProperties

setMMFFEleTerm
RDKit::MMFF::MMFFMolProperties

setMMFFOopTerm
RDKit::MMFF::MMFFMolProperties

setMMFFOStream
RDKit::MMFF::MMFFMolProperties

setMMFFStretchBendTerm
RDKit::MMFF::MMFFMolProperties

setMMFFTorsionTerm
RDKit::MMFF::MMFFMolProperties

setMMFFVariant
RDKit::MMFF::MMFFMolProperties

setMMFFVdWTerm
RDKit::MMFF::MMFFMolProperties

setMMFFVerbosity
RDKit::MMFF::MMFFMolProperties

setTwoBitCell
RDKit::MMFF::Tools

StretchBendContrib

ForceFields::MMFF::StretchBendContrib::StretchBendContrib()
ForceFields::MMFF::StretchBendContrib::StretchBendContrib(ForceField \*owner, const unsigned int idx1, const unsigned int idx2, const unsigned int idx3, const std::pair< bool, const MMFFStbn \* > mmffStbnParams, const MMFFAngle \*mmffAngleParams, const MMFFBond \*mmffBondParams1, const MMFFBond \*mmffBondParams2)

Searching...

No Matches
