# Supplementary material for: Bringing the MMFF force field to the RDKit: implementation and validation
Source: J Cheminform. 2014 Jul 12;6:37. doi: 10.1186/s13321-014-0037-3 (PMC4116604; doi:10.1186/s13321-014-0037-3)
Supplement: Additional file 3: — Documentation. The file docs.zip expands to an HTML tree which documents the MMFF-related C++ and Python RDKit APIs; the documentation can be browsed opening the docs.html file in any HTML browser. The full RDKit documentation can be found at http://www.rdkit.org. [file s13321-014-0037-3-S3.zip › docs/cpp/StretchBend_8h.html]

RDKit-MMFF: StretchBend.h File Reference


- Main Page
- Namespaces
- Classes
- Files
- Directories

- File List
- File Members

ForceField » MMFF

# StretchBend.h File Reference

`#include <utility>`  
`#include <ForceField/Contrib.h>`  

Go to the source code of this file.

|  |  |
| --- | --- |
| Classes | |
| class | ForceFields::MMFF::StretchBendContrib |
|  | The angle-bend term for MMFF. More... |
| Namespaces | |
| namespace | ForceFields |
| namespace | ForceFields::MMFF |
| namespace | ForceFields::MMFF::Utils |
| Functions | |
| std::pair< double, double > | ForceFields::MMFF::Utils::calcStbnForceConstants (const std::pair< bool, const MMFFStbn \* > mmffStbnParams) |
|  | returns the std::pair of stretch-bend force constants for an angle |
| std::pair< double, double > | ForceFields::MMFF::Utils::calcStretchBendEnergy (const double deltaDist1, const double deltaDist2, const double deltaTheta, const std::pair< double, double > forceConstants) |
|  | calculates and returns the stretch-bending MMFF energy |

---

Generated on 16 Feb 2014 for RDKit-MMFF by 
 1.6.1
