# Supplementary material for: Bringing the MMFF force field to the RDKit: implementation and validation
Source: J Cheminform. 2014 Jul 12;6:37. doi: 10.1186/s13321-014-0037-3 (PMC4116604; doi:10.1186/s13321-014-0037-3)
Supplement: Additional file 3: — Documentation. The file docs.zip expands to an HTML tree which documents the MMFF-related C++ and Python RDKit APIs; the documentation can be browsed opening the docs.html file in any HTML browser. The full RDKit documentation can be found at http://www.rdkit.org. [file s13321-014-0037-3-S3.zip › docs/cpp/StretchBend_8h_source.html]

RDKit-MMFF: StretchBend.h Source File


- Main Page
- Namespaces
- Classes
- Files
- Directories

- File List
- File Members

ForceField » MMFF

# StretchBend.h

Go to the documentation of this file.

```
00001 //
00002 //  Copyright (C) 2013 Paolo Tosco
00003 //
00004 //  Copyright (C) 2004-2006 Rational Discovery LLC
00005 //
00006 //   @@ All Rights Reserved @@
00007 //  This file is part of the RDKit.
00008 //  The contents are covered by the terms of the BSD license
00009 //  which is included in the file license.txt, found at the root
00010 //  of the RDKit source tree.
00011 //
00012 #ifndef __RD_MMFFSTRETCHBEND_H__
00013 #define __RD_MMFFSTRETCHBEND_H__
00014 
00015 #include <utility>
00016 #include <ForceField/Contrib.h>
00017 
00018 namespace ForceFields {
00019   namespace MMFF {
00020     class MMFFBond;
00021     class MMFFAngle;
00022     class MMFFStbn;
00023     class MMFFProp;
00024 
00025     //! The angle-bend term for MMFF
00026     class StretchBendContrib : public ForceFieldContrib {
00027     public:
00028       StretchBendContrib() : d_at1Idx(-1), d_at2Idx(-1), d_at3Idx(-1) {};
00029       //! Constructor
00030       /*!
00031         The angle is between atom1 - atom2 - atom3
00032         
00033         \param owner       pointer to the owning ForceField
00034         \param idx1        index of atom1 in the ForceField's positions
00035         \param idx2        index of atom2 in the ForceField's positions
00036         \param idx3        index of atom3 in the ForceField's positions
00037         \param angleType   MMFF type of the angle (as an unsigned int)
00038         
00039       */
00040     StretchBendContrib(ForceField *owner,
00041       const unsigned int idx1, const unsigned int idx2, const unsigned int idx3,
00042       const std::pair<bool, const MMFFStbn *> mmffStbnParams,
00043       const MMFFAngle *mmffAngleParams, const MMFFBond *mmffBondParams1,
00044       const MMFFBond *mmffBondParams2);
00045       
00046       double getEnergy(double *pos) const;
00047       void getGrad(double *pos,double *grad) const;
00048     
00049     private:
00050       int d_at1Idx, d_at2Idx, d_at3Idx;
00051       double d_restLen1, d_restLen2, d_theta0;
00052       std::pair<double, double> d_forceConstants;
00053     };
00054     namespace Utils {
00055       //! returns the std::pair of stretch-bend force constants for an angle 
00056       std::pair<double, double> calcStbnForceConstants
00057         (const std::pair<bool, const MMFFStbn *> mmffStbnParams);
00058       //! calculates and returns the stretch-bending MMFF energy
00059       std::pair<double, double> calcStretchBendEnergy
00060         (const double deltaDist1, const double deltaDist2,
00061         const double deltaTheta, const std::pair<double, double> forceConstants);
00062     }  
00063   }
00064 }
00065 #endif
```

---

Generated on 16 Feb 2014 for RDKit-MMFF by 
 1.6.1
