# Supplementary material for: Bringing the MMFF force field to the RDKit: implementation and validation
Source: J Cheminform. 2014 Jul 12;6:37. doi: 10.1186/s13321-014-0037-3 (PMC4116604; doi:10.1186/s13321-014-0037-3)
Supplement: Additional file 3: — Documentation. The file docs.zip expands to an HTML tree which documents the MMFF-related C++ and Python RDKit APIs; the documentation can be browsed opening the docs.html file in any HTML browser. The full RDKit documentation can be found at http://www.rdkit.org. [file s13321-014-0037-3-S3.zip › docs/cpp/TorsionAngle_8h.html]

RDKit-MMFF: TorsionAngle.h File Reference


- Main Page
- Namespaces
- Classes
- Files
- Directories

- File List
- File Members

ForceField » MMFF

# TorsionAngle.h File Reference

`#include <ForceField/Contrib.h>`  
`#include <boost/tuple/tuple.hpp>`  

Go to the source code of this file.

|  |  |
| --- | --- |
| Classes | |
| class | ForceFields::MMFF::TorsionAngleContrib |
|  | the torsion term for MMFF More... |
| Namespaces | |
| namespace | RDGeom |
| namespace | ForceFields |
| namespace | ForceFields::MMFF |
| namespace | ForceFields::MMFF::Utils |
| Functions | |
| double | ForceFields::MMFF::Utils::calcTorsionCosPhi (const RDGeom::Point3D &iPoint, const RDGeom::Point3D &jPoint, const RDGeom::Point3D &kPoint, const RDGeom::Point3D &lPoint) |
|  | calculates and returns the cosine of a torsion angle |
| boost::tuple< double, double,   double > | ForceFields::MMFF::Utils::calcTorsionForceConstant (const MMFFTor \*mmffTorParams) |
|  | returns the 3-tuple of a torsion angle force constants |
| double | ForceFields::MMFF::Utils::calcTorsionEnergy (const double V1, const double V2, const double V3, const double cosPhi) |
|  | calculates and returns the torsional MMFF energy |
| void | ForceFields::MMFF::Utils::calcTorsionGrad (RDGeom::Point3D \*r, RDGeom::Point3D \*t, double \*d, double \*\*g, double &sinTerm, double &cosPhi) |

---

Generated on 16 Feb 2014 for RDKit-MMFF by 
 1.6.1
