# Supplementary material for: Bringing the MMFF force field to the RDKit: implementation and validation
Source: J Cheminform. 2014 Jul 12;6:37. doi: 10.1186/s13321-014-0037-3 (PMC4116604; doi:10.1186/s13321-014-0037-3)
Supplement: Additional file 3: — Documentation. The file docs.zip expands to an HTML tree which documents the MMFF-related C++ and Python RDKit APIs; the documentation can be browsed opening the docs.html file in any HTML browser. The full RDKit documentation can be found at http://www.rdkit.org. [file s13321-014-0037-3-S3.zip › docs/cpp/TorsionAngle_8h_source.html]

RDKit-MMFF: TorsionAngle.h Source File


- Main Page
- Namespaces
- Classes
- Files
- Directories

- File List
- File Members

ForceField » MMFF

# TorsionAngle.h

Go to the documentation of this file.

```
00001 //
00002 //  Copyright (C) 2013 Paolo Tosco
00003 //
00004 //  Copyright (C) 2004-2006 Rational Discovery LLC
00005 //
00006 //   @@ All Rights Reserved @@
00007 //  This file is part of the RDKit.
00008 //  The contents are covered by the terms of the BSD license
00009 //  which is included in the file license.txt, found at the root
00010 //  of the RDKit source tree.
00011 //
00012 #ifndef __RD_MMFFTORSIONANGLE_H__
00013 #define __RD_MMFFTORSIONANGLE_H__
00014 
00015 #include <ForceField/Contrib.h>
00016 #include <boost/tuple/tuple.hpp>
00017 
00018 namespace RDGeom {
00019   class Point3D;
00020 }
00021 
00022 namespace ForceFields {
00023   namespace MMFF {
00024     class MMFFTor;
00025 
00026     //! the torsion term for MMFF
00027     class TorsionAngleContrib : public ForceFieldContrib {
00028     public:
00029       TorsionAngleContrib() : d_at1Idx(-1), d_at2Idx(-1), d_at3Idx(-1), d_at4Idx(-1) {};
00030       //! Constructor
00031       /*!
00032         The torsion is between atom1 - atom2 - atom3 - atom4
00033         (i.e the angle between bond atom1-atom2 and bond atom3-atom4
00034         while looking down bond atom2-atom3)
00035         
00036         \param owner       pointer to the owning ForceField
00037         \param idx1        index of atom1 in the ForceField's positions
00038         \param idx2        index of atom2 in the ForceField's positions
00039         \param idx3        index of atom3 in the ForceField's positions
00040         \param idx4        index of atom4 in the ForceField's positions
00041         \param torsionType MMFF type of the torsional bond between atoms 2 and 3
00042       */
00043       TorsionAngleContrib(ForceField *owner, unsigned int idx1, unsigned int idx2,
00044         unsigned int idx3, unsigned int idx4, const MMFFTor *mmffTorParams);
00045       double getEnergy(double *pos) const;
00046       void getGrad(double *pos, double *grad) const;
00047     private:
00048       int d_at1Idx, d_at2Idx, d_at3Idx, d_at4Idx;
00049       double d_V1, d_V2, d_V3;
00050     };
00051 
00052     namespace Utils {
00053       //! calculates and returns the cosine of a torsion angle
00054       double calcTorsionCosPhi(const RDGeom::Point3D &iPoint,
00055         const RDGeom::Point3D &jPoint, const RDGeom::Point3D &kPoint,
00056         const RDGeom::Point3D &lPoint);
00057       //! returns the 3-tuple of a torsion angle force constants
00058       boost::tuple<double, double, double>
00059         calcTorsionForceConstant(const MMFFTor *mmffTorParams);
00060       //! calculates and returns the torsional MMFF energy
00061       double calcTorsionEnergy(const double V1,
00062         const double V2, const double V3, const double cosPhi);
00063       void calcTorsionGrad(RDGeom::Point3D *r, RDGeom::Point3D *t,
00064         double *d, double **g, double &sinTerm, double &cosPhi);
00065     }
00066   }
00067 }
00068 #endif
```

---

Generated on 16 Feb 2014 for RDKit-MMFF by 
 1.6.1
