# Supplementary material for: Bringing the MMFF force field to the RDKit: implementation and validation
Source: J Cheminform. 2014 Jul 12;6:37. doi: 10.1186/s13321-014-0037-3 (PMC4116604; doi:10.1186/s13321-014-0037-3)
Supplement: Additional file 3: — Documentation. The file docs.zip expands to an HTML tree which documents the MMFF-related C++ and Python RDKit APIs; the documentation can be browsed opening the docs.html file in any HTML browser. The full RDKit documentation can be found at http://www.rdkit.org. [file s13321-014-0037-3-S3.zip › docs/cpp/TorsionConstraint_8h_source.html]

RDKit-MMFF: TorsionConstraint.h Source File


- Main Page
- Namespaces
- Classes
- Files
- Directories

- File List
- File Members

ForceField » MMFF

# TorsionConstraint.h

Go to the documentation of this file.

```
00001 //
00002 //  Copyright (C) 2013 Paolo Tosco
00003 //
00004 //  Copyright (C) 2004-2006 Rational Discovery LLC
00005 //
00006 //   @@ All Rights Reserved @@
00007 //  This file is part of the RDKit.
00008 //  The contents are covered by the terms of the BSD license
00009 //  which is included in the file license.txt, found at the root
00010 //  of the RDKit source tree.
00011 //
00012 #ifndef __RD_MMFFTORSIONCONSTRAINT_H__
00013 #define __RD_MMFFTORSIONCONSTRAINT_H__
00014 #include <iostream>
00015 #include <ForceField/Contrib.h>
00016 
00017 namespace ForceFields {
00018   namespace MMFF {
00019 
00020     //! A dihedral angle range constraint modelled after a TorsionContrib
00021     class TorsionConstraintContrib : public ForceFieldContrib {
00022     public:
00023       TorsionConstraintContrib() : d_at1Idx(-1), d_at2Idx(-1), d_at3Idx(-1), d_at4Idx(-1) {};
00024       //! Constructor
00025       /*!
00026       \param owner          pointer to the owning ForceField
00027       \param idx1           index of atom1 in the ForceField's positions
00028       \param idx2           index of atom2 in the ForceField's positions
00029       \param idx3           index of atom3 in the ForceField's positions
00030       \param idx4           index of atom4 in the ForceField's positions
00031       \param minDihedralDeg minimum dihedral angle
00032       \param maxDihedralDeg maximum dihedral angle
00033       \param forceConst     force Constant
00034         
00035       */
00036       TorsionConstraintContrib(ForceField *owner, unsigned int idx1,
00037         unsigned int idx2, unsigned int idx3, unsigned int idx4,
00038         double minDihedralDeg, double maxDihedralDeg, double forceConst);
00039       TorsionConstraintContrib(ForceField *owner, unsigned int idx1,
00040         unsigned int idx2, unsigned int idx3, unsigned int idx4,
00041         bool relative, double minDihedralDeg, double maxDihedralDeg,
00042         double forceConst);
00043 
00044       ~TorsionConstraintContrib() {
00045       }
00046       double getEnergy(double *pos) const;
00047 
00048       void getGrad(double *pos, double *grad) const;
00049     private:
00050       int d_at1Idx, d_at2Idx, d_at3Idx, d_at4Idx; //!< indices of atoms forming the dihedral angle
00051       double d_minDihedralDeg, d_maxDihedralDeg;        //!< rest amplitudes of the dihedral angle
00052       double d_forceConstant;  //!< force constant of the angle constraint
00053 
00054     };
00055   }
00056 }
00057 #endif
```

---

Generated on 16 Feb 2014 for RDKit-MMFF by 
 1.6.1
