# Supplementary material for: Bringing the MMFF force field to the RDKit: implementation and validation
Source: J Cheminform. 2014 Jul 12;6:37. doi: 10.1186/s13321-014-0037-3 (PMC4116604; doi:10.1186/s13321-014-0037-3)
Supplement: Additional file 3: — Documentation. The file docs.zip expands to an HTML tree which documents the MMFF-related C++ and Python RDKit APIs; the documentation can be browsed opening the docs.html file in any HTML browser. The full RDKit documentation can be found at http://www.rdkit.org. [file s13321-014-0037-3-S3.zip › docs/cpp/tree.html]

TreeView


All Classes Namespaces Files Functions Variables Typedefs Enumerator Defines

### RDKit-MMFF

  

Class List

ForceFields::MMFF::AngleBendContrib

ForceFields::MMFF::AngleConstraintContrib

ForceFields::MMFF::BondStretchContrib

ForceFields::MMFF::DistanceConstraintContrib

ForceFields::MMFF::EleContrib

ForceFields::MMFF::MMFFAngle

ForceFields::MMFF::MMFFAngleCollection

ForceFields::MMFF::MMFFAromCollection

RDKit::MMFF::MMFFAtomProperties

ForceFields::MMFF::MMFFBndkCollection

ForceFields::MMFF::MMFFBond

ForceFields::MMFF::MMFFBondCollection

ForceFields::MMFF::MMFFChg

ForceFields::MMFF::MMFFChgCollection

ForceFields::MMFF::MMFFCovRadPauEle

ForceFields::MMFF::MMFFCovRadPauEleCollection

ForceFields::MMFF::MMFFDef

ForceFields::MMFF::MMFFDefCollection

ForceFields::MMFF::MMFFDfsbCollection

RDKit::MMFF::MMFFMolProperties

ForceFields::MMFF::MMFFOop

ForceFields::MMFF::MMFFOopCollection

ForceFields::MMFF::MMFFPBCI

ForceFields::MMFF::MMFFPBCICollection

ForceFields::MMFF::MMFFProp

ForceFields::MMFF::MMFFPropCollection

ForceFields::MMFF::MMFFStbn

ForceFields::MMFF::MMFFStbnCollection

ForceFields::MMFF::MMFFTor

ForceFields::MMFF::MMFFTorCollection

ForceFields::MMFF::MMFFVdW

ForceFields::MMFF::MMFFVdWCollection

ForceFields::MMFF::OopBendContrib

ForceFields::MMFF::PositionConstraintContrib

ForceFields::MMFF::StretchBendContrib

ForceFields::MMFF::TorsionAngleContrib

ForceFields::MMFF::TorsionConstraintContrib

ForceFields::MMFF::VdWContrib

Class Members

Namespace List

ForceFields

ForceFields::MMFF

ForceFields::MMFF::Utils

RDGeom

RDKit

RDKit::MMFF

RDKit::MMFF::Tools

Namespace Members

File List

AngleBend.h

AngleConstraint.h

AtomTyper.h

BondStretch.h

Builder.h

Contribs.h

DistanceConstraint.h

Nonbonded.h

OopBend.h

Params.h

PositionConstraint.h

StretchBend.h

TorsionAngle.h

TorsionConstraint.h

Directory Hierarchy

ForceField

MMFF

GraphMol

ForceFieldHelpers

MMFF

File Members
