# Supplementary material for: Bringing the MMFF force field to the RDKit: implementation and validation
Source: J Cheminform. 2014 Jul 12;6:37. doi: 10.1186/s13321-014-0037-3 (PMC4116604; doi:10.1186/s13321-014-0037-3)
Supplement: Additional file 3: — Documentation. The file docs.zip expands to an HTML tree which documents the MMFF-related C++ and Python RDKit APIs; the documentation can be browsed opening the docs.html file in any HTML browser. The full RDKit documentation can be found at http://www.rdkit.org. [file s13321-014-0037-3-S3.zip › docs/docs.html]

Supplementary material

**Bringing the MMFF force field to the RDKit:  
implementation and validation**

Paolo Tosco,^†^ Nikolaus Stiefl^‡^ and Gregory Landrum^\*‡^  
  
^†^ *Department of Drug Science and Technology, University of Turin,  
Via Pietro Giuria 9, 10125 Torino, Italy*  
^‡^ *Novartis Institutes for Biomedical Research,
Basel, CH-4002, Switzerland*
  
^\*^ *Correspondence: gregory.landrum@novartis.com*

  

**Abstract**

A general purpose force field such as MMFF94/MMFF94s, which
can properly deal with a wide range of diverse structures,
is very valuable in the context of a cheminformatics toolkit.
Herein we present an open-source implementation of this force
field within the RDKit. The new MMFF functionality can be
accessed through a C++/C#/Python/Java application programming
interface (API) developed along the lines of the one already
available for UFF in the RDKit. Our implementation was fully
validated against the official validation suite provided by
the MMFF authors. All energies and gradients were correctly
computed; moreover, atom type and force constants were
correctly assigned for 3D molecules built from SMILES
strings. To provide full flexibility, the available API
provides direct access to include/exclude individual terms
from the MMFF energy expression and to carry out constrained
geometry optimizations. The availability of a MMFF-capable
molecular mechanics engine coupled with the rest of the
RDKit functionality and covered by the BSD license is
appealing to researchers operating in both academia and
industry.

  
  

**API Documentation**

C++ API
  
  
Python API
