# Supplementary material for: Bringing the MMFF force field to the RDKit: implementation and validation
Source: J Cheminform. 2014 Jul 12;6:37. doi: 10.1186/s13321-014-0037-3 (PMC4116604; doi:10.1186/s13321-014-0037-3)
Supplement: Additional file 3: — Documentation. The file docs.zip expands to an HTML tree which documents the MMFF-related C++ and Python RDKit APIs; the documentation can be browsed opening the docs.html file in any HTML browser. The full RDKit documentation can be found at http://www.rdkit.org. [file s13321-014-0037-3-S3.zip › docs/python/Chem.rdForceFieldHelpers-module.html]

xml version="1.0" encoding="ascii"?


Chem.rdForceFieldHelpers


| Trees | Indices | Help | RDKit |
| --- | --- | --- | --- |

Package Chem ::
Module rdForceFieldHelpers

[hide private]

[frames] | no frames]

# Module rdForceFieldHelpers

```
Module containing functions to handle MMFF force field
```


|  |  |
| --- | --- |
| Functions | [hide private] |

|  |  |
| --- | --- |
| MMFFGetMoleculeForceField(...)  MMFFGetMoleculeForceField( (Mol)mol, (object)pyMMFFMolProperties [, (float)nonBondedThresh=100.0 [, (int)confId=-1 [, (bool)ignoreInterfragInteractions=True]]]) -> object : returns a MMFF force field for a molecule |  |

|  |  |
| --- | --- |
| MMFFGetMoleculeProperties(...)  MMFFGetMoleculeProperties( (Mol)mol [, (str)mmffVariant='MMFF94' [, (int)mmffVerbosity=0]]) -> object : returns a PyMMFFMolProperties object for a molecule, which is required by MMFFGetMoleculeForceField() and can be used to get/set MMFF properties |  |

|  |  |
| --- | --- |
| MMFFHasAllMoleculeParams(...)  MMFFHasAllMoleculeParams( (Mol)mol) -> bool : checks if MMFF parameters are available for all of a molecule's atoms |  |

|  |  |
| --- | --- |
| MMFFOptimizeMolecule(...)  MMFFOptimizeMolecule( (Mol)mol [, (str)mmffVariant='MMFF94' [, (int)maxIters=200 [, (float)nonBondedThresh=100.0 [, (int)confId=-1 [, (bool)ignoreInterfragInteractions=True]]]]]) -> int : uses MMFF to optimize a molecule's structure |  |

|  |  |
| --- | --- |
| MMFFSanitizeMolecule(...)  MMFFSanitizeMolecule( (Mol)mol) -> int : sanitizes a molecule according to MMFF requirements. |  |


|  |  |
| --- | --- |
| Variables | [hide private] |

\_\_package\_\_ = `None`


|  |  |
| --- | --- |
| Function Details | [hide private] |

|  |  |
| --- | --- |
| MMFFGetMoleculeForceField(...) |  |

```
MMFFGetMoleculeForceField( (Mol)mol, (object)pyMMFFMolProperties [, (float)nonBondedThresh=100.0 [, (int)confId=-1 [, (bool)ignoreInterfragInteractions=True]]]) -> object :
    returns a MMFF force field for a molecule
    
     
     ARGUMENTS:
    
        - mol : the molecule of interest
        - pyMMFFMolProperties : PyMMFFMolProperties object as returned
                      by MMFFGetMoleculeProperties()
        - nonBondedThresh : used to exclude long-range non-bonded
                      interactions (defaults to 100.0)
        - confId : indicates which conformer to optimize
        - ignoreInterfragInteractions : if true, nonbonded terms between
                      fragments will not be added to the forcefield
    
    

    C++ signature :
        ForceFields::PyForceField* MMFFGetMoleculeForceField(RDKit::ROMol {lvalue},ForceFields::PyMMFFMolProperties* [,double=100.0 [,int=-1 [,bool=True]]])
```

|  |  |
| --- | --- |
| MMFFGetMoleculeProperties(...) |  |

```
MMFFGetMoleculeProperties( (Mol)mol [, (str)mmffVariant='MMFF94' [, (int)mmffVerbosity=0]]) -> object :
    returns a PyMMFFMolProperties object for a
      molecule, which is required by MMFFGetMoleculeForceField()
      and can be used to get/set MMFF properties
    
      
      ARGUMENTS:
    
        - mol : the molecule of interest
        - mmffVariant : "MMFF94" or "MMFF94s"
                      (defaults to "MMFF94")
        - mmffVerbosity : 0: none; 1: low; 2: high (defaults to 0).
    
    

    C++ signature :
        ForceFields::PyMMFFMolProperties* MMFFGetMoleculeProperties(RDKit::ROMol {lvalue} [,std::string='MMFF94' [,unsigned int=0]])
```

|  |  |
| --- | --- |
| MMFFHasAllMoleculeParams(...) |  |

```
MMFFHasAllMoleculeParams( (Mol)mol) -> bool :
    checks if MMFF parameters are available for all of a molecule's atoms
    
     
     ARGUMENTS:
    
        - mol : the molecule of interest
    
    

    C++ signature :
        bool MMFFHasAllMoleculeParams(RDKit::ROMol {lvalue})
```

|  |  |
| --- | --- |
| MMFFOptimizeMolecule(...) |  |

```
MMFFOptimizeMolecule( (Mol)mol [, (str)mmffVariant='MMFF94' [, (int)maxIters=200 [, (float)nonBondedThresh=100.0 [, (int)confId=-1 [, (bool)ignoreInterfragInteractions=True]]]]]) -> int :
    uses MMFF to optimize a molecule's structure
    
     
     ARGUMENTS:
    
        - mol : the molecule of interest
        - mmffVariant : "MMFF94" or "MMFF94s"
        - maxIters : the maximum number of iterations (defaults to 200)
        - nonBondedThresh : used to exclude long-range non-bonded
                     interactions (defaults to 100.0)
        - confId : indicates which conformer to optimize
        - ignoreInterfragInteractions : if true, nonbonded terms between
                     fragments will not be added to the forcefield
    
     RETURNS: 0 if the optimization converged, -1 if the forcefield could
              not be set up, 1 if more iterations are required.
    
    

    C++ signature :
        int MMFFOptimizeMolecule(RDKit::ROMol {lvalue} [,std::string='MMFF94' [,int=200 [,double=100.0 [,int=-1 [,bool=True]]]]])
```

|  |  |
| --- | --- |
| MMFFSanitizeMolecule(...) |  |

```
MMFFSanitizeMolecule( (Mol)mol) -> int :
    sanitizes a molecule according to MMFF requirements.
    
        - mol : the molecule of interest.
    
    

    C++ signature :
        unsigned int MMFFSanitizeMolecule(RDKit::ROMol {lvalue})
```

  


| Trees | Indices | Help | RDKit |
| --- | --- | --- | --- |

|  |  |
| --- | --- |
| Generated by Epydoc 3.0.1 on Sun Feb 16 23:58:12 2014 | http://epydoc.sourceforge.net |
