# Supplementary material for: Bringing the MMFF force field to the RDKit: implementation and validation
Source: J Cheminform. 2014 Jul 12;6:37. doi: 10.1186/s13321-014-0037-3 (PMC4116604; doi:10.1186/s13321-014-0037-3)
Supplement: Additional file 3: — Documentation. The file docs.zip expands to an HTML tree which documents the MMFF-related C++ and Python RDKit APIs; the documentation can be browsed opening the docs.html file in any HTML browser. The full RDKit documentation can be found at http://www.rdkit.org. [file s13321-014-0037-3-S3.zip › docs/python/help.html]

xml version="1.0" encoding="ascii"?


Help


| Trees | Indices | Help | RDKit |
| --- | --- | --- | --- |

[hide private]

[frames] | no frames]

# Bringing the MMFF force field to the RDKit: implementation and validation

Paolo Tosco,^†^ Nikolaus Stiefl^‡^ and Gregory Landrum^\*‡^  
  
^†^ *Department of Drug Science and Technology, University of Turin,  
Via Pietro Giuria 9, 10125 Torino, Italy*  
^‡^ *Novartis Institutes for Biomedical Research,
Basel, CH-4002, Switzerland*
  
^\*^ *Correspondence: gregory.landrum@novartis.com*

## Abstract

A general purpose force field such as MMFF94/MMFF94s, which
can properly deal with a wide range of diverse structures,
is very valuable in the context of a cheminformatics toolkit.
Herein we present an open-source implementation of this force
field within the RDKit. The new MMFF functionality can be
accessed through a C++/C#/Python/Java application programming
interface (API) developed along the lines of the one already
available for UFF in the RDKit. Our implementation was fully
validated against the official validation suite provided by
the MMFF authors. All energies and gradients were correctly
computed; moreover, atom type and force constants were
correctly assigned for 3D molecules built from SMILES
strings. To provide full flexibility, the available API
provides direct access to include/exclude individual terms
from the MMFF energy expression and to carry out constrained
geometry optimizations. The availability of a MMFF-capable
molecular mechanics engine coupled with the rest of the
RDKit functionality and covered by the BSD license is
appealing to researchers operating in both academia and
industry.

  
  

# API Documentation

This document contains the API (Application Programming Interface)
documentation for RDPython-MMFF. Documentation for the Python
objects defined by the project is divided into separate pages for each
package, module, and class. The API documentation also includes two
pages containing information about the project as a whole: a trees
page, and an index page.

## Object Documentation

Each **Package Documentation** page contains:

- A description of the package.
- A list of the modules and sub-packages contained by the
  package.
- A summary of the classes defined by the package.
- A summary of the functions defined by the package.
- A summary of the variables defined by the package.
- A detailed description of each function defined by the
  package.
- A detailed description of each variable defined by the
  package.

Each **Module Documentation** page contains:

- A description of the module.
- A summary of the classes defined by the module.
- A summary of the functions defined by the module.
- A summary of the variables defined by the module.
- A detailed description of each function defined by the
  module.
- A detailed description of each variable defined by the
  module.

Each **Class Documentation** page contains:

- A class inheritance diagram.
- A list of known subclasses.
- A description of the class.
- A summary of the methods defined by the class.
- A summary of the instance variables defined by the class.
- A summary of the class (static) variables defined by the
  class.
- A detailed description of each method defined by the
  class.
- A detailed description of each instance variable defined by the
  class.
- A detailed description of each class (static) variable defined
  by the class.

## Project Documentation

The **Trees** page contains the module and class hierarchies:

- The *module hierarchy* lists every package and module, with
  modules grouped into packages. At the top level, and within each
  package, modules and sub-packages are listed alphabetically.
- The *class hierarchy* lists every class, grouped by base
  class. If a class has more than one base class, then it will be
  listed under each base class. At the top level, and under each base
  class, classes are listed alphabetically.

The **Index** page contains indices of terms and
identifiers:

- The *term index* lists every term indexed by any object's
  documentation. For each term, the index provides links to each
  place where the term is indexed.
- The *identifier index* lists the (short) name of every package,
  module, class, method, function, variable, and parameter. For each
  identifier, the index provides a short description, and a link to
  its documentation.

## The Table of Contents

The table of contents occupies the two frames on the left side of
the window. The upper-left frame displays the *project
contents*, and the lower-left frame displays the *module
contents*:

|  |  |
| --- | --- |
| Project | API |
| Module |  |
