# Supplementary material for: Bringing the MMFF force field to the RDKit: implementation and validation
Source: J Cheminform. 2014 Jul 12;6:37. doi: 10.1186/s13321-014-0037-3 (PMC4116604; doi:10.1186/s13321-014-0037-3)
Supplement: Additional file 3: — Documentation. The file docs.zip expands to an HTML tree which documents the MMFF-related C++ and Python RDKit APIs; the documentation can be browsed opening the docs.html file in any HTML browser. The full RDKit documentation can be found at http://www.rdkit.org. [file s13321-014-0037-3-S3.zip › docs/python/identifier-index.html]

xml version="1.0" encoding="ascii"?


Identifier Index


| Trees | Indices | Help | RDKit |
| --- | --- | --- | --- |

[hide private]

[frames] | no frames]

|  |  |
| --- | --- |
| Identifier Index | [ A B C D E F G H I J K L M N O P Q R S T U V W X Y Z \_ ] |

|  |  |
| --- | --- |
| A | AddDistanceConstraint() (in ForceField) \| AddExtraPoint() (in ForceField) \| AddFixedPoint() (in ForceField) |
| C | CalcEnergy() (in ForceField) \| \| |
| F | ForceField (in rdkit) \| ForceField (in rdkit.ForceField.rdForceField) \| |
| G | GetExtraPointPos() (in ForceField) \| GetMMFFFormalCharge() (in MMFFMolProperties) \|; GetMMFFAtomType() (in MMFFMolProperties) \| GetMMFFPartialCharge() (in MMFFMolProperties) \| |
| I | Initialize() (in ForceField) \| \| |
| M | Minimize() (in ForceField) \| MMFFAddTorsionConstraint() (in ForceField) \| MMFFMolProperties (in rdkit.ForceField.rdForceField); MMFFAddAngleConstraint() (in ForceField) \| MMFFGetMoleculeForceField() (in Chem.rdForceFieldHelpers) \| MMFFOptimizeMolecule() (in Chem.rdForceFieldHelpers); MMFFAddDistanceConstraint() (in ForceField) \| MMFFGetMoleculeProperties() (in Chem.rdForceFieldHelpers) \| MMFFSanitizeMolecule() (in Chem.rdForceFieldHelpers); MMFFAddPositionConstraint() (in ForceField) \| MMFFHasAllMoleculeParams() (in Chem.rdForceFieldHelpers) \| |
| R | rdForceField (in rdkit.ForceField) \| rdForceFieldHelpers (in Chem) \| |
| S | SetMMFFAngleTerm() (in MMFFMolProperties) \| SetMMFFEleTerm() (in MMFFMolProperties) \| SetMMFFVariant() (in MMFFMolProperties); SetMMFFBondTerm() (in MMFFMolProperties) \| SetMMFFOopTerm() (in MMFFMolProperties) \| SetMMFFVdWTerm() (in MMFFMolProperties); SetMMFFDielectricConstant() (in MMFFMolProperties) \| SetMMFFStretchBendTerm() (in MMFFMolProperties) \| SetMMFFVerbosity() (in MMFFMolProperties); SetMMFFDielectricModel() (in MMFFMolProperties) \| SetMMFFTorsionTerm() (in MMFFMolProperties) \| |
| \_ | \_\_init\_\_() (in ForceField) \| \_\_package\_\_ (in Chem.rdForceFieldHelpers) \| \_\_package\_\_ (in rdkit.ForceField.rdForceField); \_\_init\_\_() (in MMFFMolProperties) \| \_\_package\_\_ (in rdkit.ForceField) \| \_\_reduce\_\_() (in MMFFMolProperties) |

  
  

| Trees | Indices | Help | RDKit |
| --- | --- | --- | --- |

|  |  |
| --- | --- |
| Generated by Epydoc 3.0.1 on Sun Feb 16 23:58:12 2014 | http://epydoc.sourceforge.net |
