# Supplementary material for: Bringing the MMFF force field to the RDKit: implementation and validation
Source: J Cheminform. 2014 Jul 12;6:37. doi: 10.1186/s13321-014-0037-3 (PMC4116604; doi:10.1186/s13321-014-0037-3)
Supplement: Additional file 3: — Documentation. The file docs.zip expands to an HTML tree which documents the MMFF-related C++ and Python RDKit APIs; the documentation can be browsed opening the docs.html file in any HTML browser. The full RDKit documentation can be found at http://www.rdkit.org. [file s13321-014-0037-3-S3.zip › docs/python/rdkit.ForceField.rdForceField.ForceField-class.html]

xml version="1.0" encoding="ascii"?


rdkit.ForceField.rdForceField.ForceField


| Home | Trees | Indices | Help | RDKit |
| --- | --- | --- | --- | --- |

Package rdkit ::
Package ForceField ::
Module rdForceField ::
Class ForceField

[hide private]

[frames] | no frames]

# Class ForceField

```
 object --+    
          |    
??.instance --+
              |
             ForceField
```

---

```
A force field
```


|  |  |
| --- | --- |
| Instance Methods | [hide private] |

|  |  |
| --- | --- |
| AddExtraPoint(...)  AddExtraPoint( (ForceField)self, (float)x, (float)y, (float)z [, (bool)fixed=True]) -> int : Adds an extra point, this can be useful for adding constraints. |  |

|  |  |
| --- | --- |
| AddFixedPoint(...)  AddFixedPoint( (ForceField)self, (int)idx) -> None : Adds a fixed point to the force field. |  |

|  |  |
| --- | --- |
| CalcEnergy(...)  CalcEnergy( (ForceField)arg1) -> float : Returns the energy of the current arrangement |  |

|  |  |
| --- | --- |
| GetExtraPointPos(...)  GetExtraPointPos( (ForceField)self, (int)idx) -> object : returns the location of an extra point as a tuple |  |

|  |  |
| --- | --- |
| Initialize(...)  Initialize( (ForceField)arg1) -> None : initializes the force field (call this before minimizing) |  |

|  |  |
| --- | --- |
| MMFFAddAngleConstraint(...)  MMFFAddAngleConstraint( (ForceField)self, (int)idx1, (int)idx2, (int)idx3, (bool)relative, (float)minAngleDeg, (float)maxAngleDeg, (float)forceConstant) -> None : Adds an angle constraint to the MMFF force field; if relative == True, then minAngleDeg and maxAngleDeg are intended as relative to the current angle. |  |

|  |  |
| --- | --- |
| MMFFAddDistanceConstraint(...)  MMFFAddDistanceConstraint( (ForceField)self, (int)idx1, (int)idx2, (bool)relative, (float)minLen, (float)maxLen, (float)forceConstant) -> None : Adds a distance constraint to the MMFF force field; if relative == True, then minLen and maxLen are intended as relative to the current distance. |  |

|  |  |
| --- | --- |
| MMFFAddPositionConstraint(...)  MMFFAddPositionConstraint( (ForceField)self, (int)idx, (float)maxDispl, (float)forceConstant) -> None : Adds a position constraint to the MMFF force field. |  |

|  |  |
| --- | --- |
| MMFFAddTorsionConstraint(...)  MMFFAddTorsionConstraint( (ForceField)self, (int)idx1, (int)idx2, (int)idx3, (int)idx4, (bool)relative, (float)minDihedralDeg, (float)maxDihedralDeg, (float)forceConstant) -> None : Adds a dihedral angle constraint to the MMFF force field; if relative == True, then minDihedralDeg and maxDihedralDeg are intended as relative to the current dihedral angle. |  |

|  |  |
| --- | --- |
| Minimize(...)  Minimize( (ForceField)arg1 [, (int)maxIts=200 [, (float)forceTol=0.0001 [, (float)energyTol=9.9999999999999995e-07]]]) -> int : Runs some minimization iterations. |  |

|  |  |
| --- | --- |
| \_\_init\_\_(...)  Raises an exception... |  |

|  |  |
| --- | --- |
| \_\_reduce\_\_(...)  helper for pickle |  |

**Inherited from `unreachable.instance`**:
`__new__`

**Inherited from `object`**:
`__delattr__`,
`__format__`,
`__getattribute__`,
`__hash__`,
`__reduce_ex__`,
`__repr__`,
`__setattr__`,
`__sizeof__`,
`__str__`,
`__subclasshook__`


|  |  |
| --- | --- |
| Properties | [hide private] |

**Inherited from `object`**:
`__class__`


|  |  |
| --- | --- |
| Method Details | [hide private] |

|  |  |
| --- | --- |
| AddExtraPoint(...) |  |

```
AddExtraPoint( (ForceField)self, (float)x, (float)y, (float)z [, (bool)fixed=True]) -> int :
    Adds an extra point, this can be useful for adding constraints.

    C++ signature :
        int AddExtraPoint(ForceFields::PyForceField {lvalue},double,double,double [,bool=True])
```

|  |  |
| --- | --- |
| AddFixedPoint(...) |  |

```
AddFixedPoint( (ForceField)self, (int)idx) -> None :
    Adds a fixed point to the force field.

    C++ signature :
        void AddFixedPoint(ForceFields::PyForceField*,unsigned int)
```

|  |  |
| --- | --- |
| CalcEnergy(...) |  |

```
CalcEnergy( (ForceField)arg1) -> float :
    Returns the energy of the current arrangement

    C++ signature :
        double CalcEnergy(ForceFields::PyForceField {lvalue})
```

|  |  |
| --- | --- |
| GetExtraPointPos(...) |  |

```
GetExtraPointPos( (ForceField)self, (int)idx) -> object :
    returns the location of an extra point as a tuple

    C++ signature :
        _object* GetExtraPointPos(ForceFields::PyForceField*,unsigned int)
```

|  |  |
| --- | --- |
| Initialize(...) |  |

```
Initialize( (ForceField)arg1) -> None :
    initializes the force field (call this before minimizing)

    C++ signature :
        void Initialize(ForceFields::PyForceField {lvalue})
```

|  |  |
| --- | --- |
| MMFFAddAngleConstraint(...) |  |

```
MMFFAddAngleConstraint( (ForceField)self, (int)idx1, (int)idx2, (int)idx3, (bool)relative, (float)minAngleDeg, (float)maxAngleDeg, (float)forceConstant) -> None :
    Adds an angle constraint to the MMFF force field; if relative == True, then minAngleDeg and maxAngleDeg are intended as relative to the current angle.

    C++ signature :
        void MMFFAddAngleConstraint(ForceFields::PyForceField*,unsigned int,unsigned int,unsigned int,bool,double,double,double)
```

|  |  |
| --- | --- |
| MMFFAddDistanceConstraint(...) |  |

```
MMFFAddDistanceConstraint( (ForceField)self, (int)idx1, (int)idx2, (bool)relative, (float)minLen, (float)maxLen, (float)forceConstant) -> None :
    Adds a distance constraint to the MMFF force field; if relative == True, then minLen and maxLen are intended as relative to the current distance.

    C++ signature :
        void MMFFAddDistanceConstraint(ForceFields::PyForceField*,unsigned int,unsigned int,bool,double,double,double)
```

|  |  |
| --- | --- |
| MMFFAddPositionConstraint(...) |  |

```
MMFFAddPositionConstraint( (ForceField)self, (int)idx, (float)maxDispl, (float)forceConstant) -> None :
    Adds a position constraint to the MMFF force field.

    C++ signature :
        void MMFFAddPositionConstraint(ForceFields::PyForceField*,unsigned int,double,double)
```

|  |  |
| --- | --- |
| MMFFAddTorsionConstraint(...) |  |

```
MMFFAddTorsionConstraint( (ForceField)self, (int)idx1, (int)idx2, (int)idx3, (int)idx4, (bool)relative, (float)minDihedralDeg, (float)maxDihedralDeg, (float)forceConstant) -> None :
    Adds a dihedral angle constraint to the MMFF force field; if relative == True, then minDihedralDeg and maxDihedralDeg are intended as relative to the current dihedral angle.

    C++ signature :
        void MMFFAddTorsionConstraint(ForceFields::PyForceField*,unsigned int,unsigned int,unsigned int,unsigned int,bool,double,double,double)
```

|  |  |
| --- | --- |
| Minimize(...) |  |

```
Minimize( (ForceField)arg1 [, (int)maxIts=200 [, (float)forceTol=0.0001 [, (float)energyTol=9.9999999999999995e-07]]]) -> int :
    Runs some minimization iterations.
    
      Returns 0 if the minimization succeeded.

    C++ signature :
        int Minimize(ForceFields::PyForceField {lvalue} [,int=200 [,double=0.0001 [,double=9.9999999999999995e-07]]])
```

|  |  |
| --- | --- |
| \_\_init\_\_(...)  *(Constructor)* |  |

```
Raises an exception
This class cannot be instantiated from Python
```

Overrides: object.\_\_init\_\_

|  |  |
| --- | --- |
| \_\_reduce\_\_(...) |  |

```
helper for pickle
```

Overrides: object.\_\_reduce\_\_: *(inherited documentation)*

  


| Home | Trees | Indices | Help | RDKit |
| --- | --- | --- | --- | --- |

|  |  |
| --- | --- |
| Generated by Epydoc 3.0.1 on Sun Feb 16 23:48:59 2014 | http://epydoc.sourceforge.net |
