# Supplementary material for: Bringing the MMFF force field to the RDKit: implementation and validation
Source: J Cheminform. 2014 Jul 12;6:37. doi: 10.1186/s13321-014-0037-3 (PMC4116604; doi:10.1186/s13321-014-0037-3)
Supplement: Additional file 3: — Documentation. The file docs.zip expands to an HTML tree which documents the MMFF-related C++ and Python RDKit APIs; the documentation can be browsed opening the docs.html file in any HTML browser. The full RDKit documentation can be found at http://www.rdkit.org. [file s13321-014-0037-3-S3.zip › docs/python/rdkit.ForceField.rdForceField.MMFFMolProperties-class.html]

xml version="1.0" encoding="ascii"?


rdkit.ForceField.rdForceField.MMFFMolProperties


| Trees | Indices | Help | RDKit |
| --- | --- | --- | --- |

Package rdkit ::
Package ForceField ::
Module rdForceField ::
Class MMFFMolProperties

[hide private]

[frames] | no frames]

# Class MMFFMolProperties

```
 object --+    
          |    
??.instance --+
              |
             MMFFMolProperties
```

---

```
MMFF molecular properties
```


|  |  |
| --- | --- |
| Instance Methods | [hide private] |

|  |  |
| --- | --- |
| GetMMFFAtomType(...)  GetMMFFAtomType( (MMFFMolProperties)self, (int)idx) -> int : Retrieves MMFF atom type for atom with index idx |  |

|  |  |
| --- | --- |
| GetMMFFFormalCharge(...)  GetMMFFFormalCharge( (MMFFMolProperties)self, (int)idx) -> float : Retrieves MMFF formal charge for atom with index idx |  |

|  |  |
| --- | --- |
| GetMMFFPartialCharge(...)  GetMMFFPartialCharge( (MMFFMolProperties)self, (int)idx) -> float : Retrieves MMFF partial charge for atom with index idx |  |

|  |  |
| --- | --- |
| SetMMFFAngleTerm(...)  SetMMFFAngleTerm( (MMFFMolProperties)self [, (bool)state=True]) -> None : Sets the angle term to be included in the MMFF equation (defaults to True) |  |

|  |  |
| --- | --- |
| SetMMFFBondTerm(...)  SetMMFFBondTerm( (MMFFMolProperties)self [, (bool)state=True]) -> None : Sets the bond term to be included in the MMFF equation (defaults to True) |  |

|  |  |
| --- | --- |
| SetMMFFDielectricConstant(...)  SetMMFFDielectricConstant( (MMFFMolProperties)self [, (float)dielConst=1.0]) -> None : Sets the DielConst MMFF property (defaults to 1.0) |  |

|  |  |
| --- | --- |
| SetMMFFDielectricModel(...)  SetMMFFDielectricModel( (MMFFMolProperties)self [, (int)dielModel=1]) -> None : Sets the DielModel MMFF property (1: constant; 2: distance-dependent; defaults to constant) |  |

|  |  |
| --- | --- |
| SetMMFFEleTerm(...)  SetMMFFEleTerm( (MMFFMolProperties)self [, (bool)state=True]) -> None : Sets the electrostatic term to be included in the MMFF equation (defaults to True) |  |

|  |  |
| --- | --- |
| SetMMFFOopTerm(...)  SetMMFFOopTerm( (MMFFMolProperties)self [, (bool)state=True]) -> None : Sets the out-of-plane bend term to be included in the MMFF equation (defaults to True) |  |

|  |  |
| --- | --- |
| SetMMFFStretchBendTerm(...)  SetMMFFStretchBendTerm( (MMFFMolProperties)self [, (bool)state=True]) -> None : Sets the stretch-bend term to be included in the MMFF equation (defaults to True) |  |

|  |  |
| --- | --- |
| SetMMFFTorsionTerm(...)  SetMMFFTorsionTerm( (MMFFMolProperties)self [, (bool)state=True]) -> None : Sets the torsional term to be included in the MMFF equation (defaults to True) |  |

|  |  |
| --- | --- |
| SetMMFFVariant(...)  SetMMFFVariant( (MMFFMolProperties)self [, (str)mmffVariant='MMFF94']) -> None : Sets the MMFF variant to be used ("MMFF94" or "MMFF94s"; defaults to "MMFF94") |  |

|  |  |
| --- | --- |
| SetMMFFVdWTerm(...)  SetMMFFVdWTerm( (MMFFMolProperties)self [, (bool)state=True]) -> None : Sets the Van der Waals term to be included in the MMFF equation (defaults to True) |  |

|  |  |
| --- | --- |
| SetMMFFVerbosity(...)  SetMMFFVerbosity( (MMFFMolProperties)self [, (int)verbosity=0]) -> None : Sets the MMFF verbosity (0: none; 1: low; 2: high; defaults to 0) |  |

|  |  |
| --- | --- |
| \_\_init\_\_(...)  Raises an exception... |  |

|  |  |
| --- | --- |
| \_\_reduce\_\_(...)  helper for pickle |  |

**Inherited from `unreachable.instance`**:
`__new__`

**Inherited from `object`**:
`__delattr__`,
`__format__`,
`__getattribute__`,
`__hash__`,
`__reduce_ex__`,
`__repr__`,
`__setattr__`,
`__sizeof__`,
`__str__`,
`__subclasshook__`


|  |  |
| --- | --- |
| Properties | [hide private] |

**Inherited from `object`**:
`__class__`


|  |  |
| --- | --- |
| Method Details | [hide private] |

|  |  |
| --- | --- |
| GetMMFFAtomType(...) |  |

```
GetMMFFAtomType( (MMFFMolProperties)self, (int)idx) -> int :
    Retrieves MMFF atom type for atom with index idx

    C++ signature :
        unsigned int GetMMFFAtomType(ForceFields::PyMMFFMolProperties {lvalue},unsigned int)
```

|  |  |
| --- | --- |
| GetMMFFFormalCharge(...) |  |

```
GetMMFFFormalCharge( (MMFFMolProperties)self, (int)idx) -> float :
    Retrieves MMFF formal charge for atom with index idx

    C++ signature :
        double GetMMFFFormalCharge(ForceFields::PyMMFFMolProperties {lvalue},unsigned int)
```

|  |  |
| --- | --- |
| GetMMFFPartialCharge(...) |  |

```
GetMMFFPartialCharge( (MMFFMolProperties)self, (int)idx) -> float :
    Retrieves MMFF partial charge for atom with index idx

    C++ signature :
        double GetMMFFPartialCharge(ForceFields::PyMMFFMolProperties {lvalue},unsigned int)
```

|  |  |
| --- | --- |
| SetMMFFAngleTerm(...) |  |

```
SetMMFFAngleTerm( (MMFFMolProperties)self [, (bool)state=True]) -> None :
    Sets the angle term to be included in the MMFF equation (defaults to True)

    C++ signature :
        void SetMMFFAngleTerm(ForceFields::PyMMFFMolProperties {lvalue} [,bool=True])
```

|  |  |
| --- | --- |
| SetMMFFBondTerm(...) |  |

```
SetMMFFBondTerm( (MMFFMolProperties)self [, (bool)state=True]) -> None :
    Sets the bond term to be included in the MMFF equation (defaults to True)

    C++ signature :
        void SetMMFFBondTerm(ForceFields::PyMMFFMolProperties {lvalue} [,bool=True])
```

|  |  |
| --- | --- |
| SetMMFFDielectricConstant(...) |  |

```
SetMMFFDielectricConstant( (MMFFMolProperties)self [, (float)dielConst=1.0]) -> None :
    Sets the DielConst MMFF property (defaults to 1.0)

    C++ signature :
        void SetMMFFDielectricConstant(ForceFields::PyMMFFMolProperties {lvalue} [,double=1.0])
```

|  |  |
| --- | --- |
| SetMMFFDielectricModel(...) |  |

```
SetMMFFDielectricModel( (MMFFMolProperties)self [, (int)dielModel=1]) -> None :
    Sets the DielModel MMFF property (1: constant; 2: distance-dependent; defaults to constant)

    C++ signature :
        void SetMMFFDielectricModel(ForceFields::PyMMFFMolProperties {lvalue} [,unsigned char=1])
```

|  |  |
| --- | --- |
| SetMMFFEleTerm(...) |  |

```
SetMMFFEleTerm( (MMFFMolProperties)self [, (bool)state=True]) -> None :
    Sets the electrostatic term to be included in the MMFF equation (defaults to True)

    C++ signature :
        void SetMMFFEleTerm(ForceFields::PyMMFFMolProperties {lvalue} [,bool=True])
```

|  |  |
| --- | --- |
| SetMMFFOopTerm(...) |  |

```
SetMMFFOopTerm( (MMFFMolProperties)self [, (bool)state=True]) -> None :
    Sets the out-of-plane bend term to be included in the MMFF equation (defaults to True)

    C++ signature :
        void SetMMFFOopTerm(ForceFields::PyMMFFMolProperties {lvalue} [,bool=True])
```

|  |  |
| --- | --- |
| SetMMFFStretchBendTerm(...) |  |

```
SetMMFFStretchBendTerm( (MMFFMolProperties)self [, (bool)state=True]) -> None :
    Sets the stretch-bend term to be included in the MMFF equation (defaults to True)

    C++ signature :
        void SetMMFFStretchBendTerm(ForceFields::PyMMFFMolProperties {lvalue} [,bool=True])
```

|  |  |
| --- | --- |
| SetMMFFTorsionTerm(...) |  |

```
SetMMFFTorsionTerm( (MMFFMolProperties)self [, (bool)state=True]) -> None :
    Sets the torsional term to be included in the MMFF equation (defaults to True)

    C++ signature :
        void SetMMFFTorsionTerm(ForceFields::PyMMFFMolProperties {lvalue} [,bool=True])
```

|  |  |
| --- | --- |
| SetMMFFVariant(...) |  |

```
SetMMFFVariant( (MMFFMolProperties)self [, (str)mmffVariant='MMFF94']) -> None :
    Sets the MMFF variant to be used ("MMFF94" or "MMFF94s"; defaults to "MMFF94")

    C++ signature :
        void SetMMFFVariant(ForceFields::PyMMFFMolProperties {lvalue} [,std::string='MMFF94'])
```

|  |  |
| --- | --- |
| SetMMFFVdWTerm(...) |  |

```
SetMMFFVdWTerm( (MMFFMolProperties)self [, (bool)state=True]) -> None :
    Sets the Van der Waals term to be included in the MMFF equation (defaults to True)

    C++ signature :
        void SetMMFFVdWTerm(ForceFields::PyMMFFMolProperties {lvalue} [,bool=True])
```

|  |  |
| --- | --- |
| SetMMFFVerbosity(...) |  |

```
SetMMFFVerbosity( (MMFFMolProperties)self [, (int)verbosity=0]) -> None :
    Sets the MMFF verbosity (0: none; 1: low; 2: high; defaults to 0)

    C++ signature :
        void SetMMFFVerbosity(ForceFields::PyMMFFMolProperties {lvalue} [,unsigned int=0])
```

|  |  |
| --- | --- |
| \_\_init\_\_(...)  *(Constructor)* |  |

```
Raises an exception
This class cannot be instantiated from Python
```

Overrides: object.\_\_init\_\_

|  |  |
| --- | --- |
| \_\_reduce\_\_(...) |  |

```
helper for pickle
```

Overrides: object.\_\_reduce\_\_: *(inherited documentation)*

  


| Trees | Indices | Help | RDKit |
| --- | --- | --- | --- |

|  |  |
| --- | --- |
| Generated by Epydoc 3.0.1 on Sun Feb 16 23:58:12 2014 | http://epydoc.sourceforge.net |
