# Supplementary material for: Bringing the MMFF force field to the RDKit: implementation and validation
Source: J Cheminform. 2014 Jul 12;6:37. doi: 10.1186/s13321-014-0037-3 (PMC4116604; doi:10.1186/s13321-014-0037-3)
Supplement: Additional file 3: — Documentation. The file docs.zip expands to an HTML tree which documents the MMFF-related C++ and Python RDKit APIs; the documentation can be browsed opening the docs.html file in any HTML browser. The full RDKit documentation can be found at http://www.rdkit.org. [file s13321-014-0037-3-S3.zip › docs/python/rdkit.ForceField-module.html]

xml version="1.0" encoding="ascii"?


rdkit.ForceField


| Trees | Indices | Help | RDKit |
| --- | --- | --- | --- |

Package rdkit ::
Package ForceField

[hide private]

[frames] | no frames]

# Package ForceField

source code


|  |  |
| --- | --- |
| Submodules | [hide private] |

- **rdkit.ForceField.rdForceField**: *Exposes the ForceField class*

  


|  |  |
| --- | --- |
| Variables | [hide private] |

\_\_package\_\_ = `'rdkit.ForceField'`

**Imports:**
ForceField,
MMFFMolProperties,
rdForceField

  


| Trees | Indices | Help | RDKit |
| --- | --- | --- | --- |

|  |  |
| --- | --- |
| Generated by Epydoc 3.0.1 on Sun Feb 16 23:58:12 2014 | http://epydoc.sourceforge.net |
