# Supplementary material for: Bringing the MMFF force field to the RDKit: implementation and validation
Source: J Cheminform. 2014 Jul 12;6:37. doi: 10.1186/s13321-014-0037-3 (PMC4116604; doi:10.1186/s13321-014-0037-3)
Supplement: Additional file 3: — Documentation. The file docs.zip expands to an HTML tree which documents the MMFF-related C++ and Python RDKit APIs; the documentation can be browsed opening the docs.html file in any HTML browser. The full RDKit documentation can be found at http://www.rdkit.org. [file s13321-014-0037-3-S3.zip › docs/python/toc-everything.html]

xml version="1.0" encoding="ascii"?


Everything


# Everything

---

## All Classes

rdkit.ForceField.rdForceField.ForceField  
 rdkit.ForceField.rdForceField.MMFFMolProperties  

## All Functions

Chem.rdForceFieldHelpers.MMFFGetMoleculeForceField  
 Chem.rdForceFieldHelpers.MMFFGetMoleculeProperties  
 Chem.rdForceFieldHelpers.MMFFHasAllMoleculeParams  
 Chem.rdForceFieldHelpers.MMFFOptimizeMolecule  
 Chem.rdForceFieldHelpers.MMFFSanitizeMolecule  

## All Variables

Chem.rdForceFieldHelpers.\_\_package\_\_  
 rdkit.ForceField.\_\_package\_\_  
 rdkit.ForceField.rdForceField.\_\_package\_\_  

---

[hide private]
